# Multilocus sequence typing of *Cryptococcus neoformans* var. *grubii* from Laos in a regional and global context

**DOI:** 10.1093/mmy/myy105

**Published:** 2018-10-19

**Authors:** Lam Tuan Thanh, Trieu Hai Phan, Sayaphet Rattanavong, Trinh Mai Nguyen, Anh Van Duong, Cherrelle Dacon, Thu Nha Hoang, Lan Phu Huong Nguyen, Chau Thi Hong Tran, Viengmon Davong, Chau Van Vinh Nguyen, Guy E Thwaites, Maciej F Boni, David Dance, Philip M Ashton, Jeremy N Day

**Affiliations:** 1 Oxford University Clinical Research Unit, Vietnam; 2 Laos-Oxford-Mahosot Hospital Wellcome Trust Research Unit, Lao People's Democratic Republic; 3 Sir William Dunn School of Pathology, University of Oxford, UK; 4 Hospital for Tropical Diseases, Ho Chi Minh City, Vietnam; 5 Nuffield Department of Medicine, Oxford University, UK; 6 Department of Biology, Pennsylvania State University, USA; 7 Faculty of Infectious and Tropical Diseases, London School of Hygiene and Tropical Medicine, London, UK

**Keywords:** *Cryptococcus neoformans*, multilocus sequence typing, Laos, Vietnam, population structure

## Abstract

Cryptococcosis causes approximately 180 000 deaths each year in patients with human immunodeficiency virus (HIV). Patients with other forms of immunosuppression are also at risk, and disease is increasingly recognized in apparently immunocompetent individuals. *Cryptococcus neoformans* var. *grubii*, responsible for the majority of cases, is distributed globally. We used the consensus ISHAM Multilocus sequence typing (MLST) scheme to define the population structure of clinical *C. neoformans* var. *grubii* isolates from Laos (*n* = 81), which we placed into the global context using published MLST data from other countries (total N = 1047), including a reanalysis of 136 Vietnamese isolates previously reported. We observed a phylogeographical relationship in which the Laotian population was similar to its neighbor Thailand, being dominated (83%) by Sequence Types (ST) 4 and 6. This phylogeographical structure changed moving eastwards, with Vietnam's population consisting of an admixture of isolates dominated by the ST4/ST6 (35%) and ST5 (48%) lineages. The ST5 lineage is the predominant ST reported from China and East Asia, where it accounts for >90% of isolates. Analysis of genetic distance (*Fst*) between different populations of *C. neoformans* var. *grubii* supports this intermediate structure of the Vietnamese population. The pathogen and host diversity reported from Vietnam provide the strongest epidemiological evidence of the association between ST5 and HIV-uninfected patients. Regional anthropological genetic distances suggest diversity in the *C. neoformans* var. *grubii* population across Southeast Asia is driven by ecological rather than human host factors. Where the ST5 lineage is present, disease in HIV-uninfected patients is to be expected.

## Introduction

*Cryptococcus neoformans* is the main etiological agent of cryptococcosis. It is of major significance in patients infected with human immunodeficiency virus (HIV), accounting for an estimated 223 100 cases of meningitis per year globally, and in other individuals with immunosuppression.^1^*C. neoformans* is an environmental saprophyte associated with bird guano and trees.^2–5^ There is no human to human spread, and human infection is believed to result from the inhalation of either desiccated yeasts or spores.^6^ In Asia, the majority of cases of cryptococcal meningitis are due to *C. neoformans* var. *grubii.*^7–10^ Here, in addition to the strong association with HIV infection, *C. neoformans* var. *grubii* is also reported to cause disease in apparently healthy individuals.^11,12^ Such cases have been reported from China, Japan, Korea, and Vietnam and are typically associated with a clonal group known as VNI-gamma or ST5.^7,13–16^

The clinical and environmental *C. neoformans* var. *grubii* isolates from South, East and Southeast Asia exhibit a clonal population structure.^17,18^ Multilocus sequence typing (MLST) epidemiological studies from East Asia have shown limited genetic diversity among the majority of environmental and clinical isolates.^13,19,20^ Different geographical areas have distinct population structures, particularly in relation to the predominant sequence types.^17^ Previous studies suggested that though in relatively close geographical proximity, the population structure of *C. neoformans* var. *grubii* from Thailand and China are significantly different.^21,22^ We recently reported the population structure of *C. neoformans* var. *grubii* from Vietnam,^23^ but analyses focused on associations between clinical features and population structure. Here we present novel data from Laos and novel analyses of our previously published Vietnamese data set.

Our specific goals were (i) to describe the genetic structure of the Laotian *C. neoformans* var. *grubii* population using MLST, and (ii) to set the Vietnamese and Laotian populations within the global context.

## Methods

### Ethics statement

All studies from which samples were derived were approved by the relevant institutional review board: the Hospital for Tropical Diseases Ethical Review Board, Ho Chi Minh City, the National Ethics Committee for Health Research Vietnam, the Ministry of Health, Lao People's Democratic Republic, and either the Oxford Tropical Ethics Committee, University of Oxford, or the Ethics Committee of the Liverpool School of Tropical Medicine. All patients were adults and gave written informed consent to enter the clinical studies. Where the patient lacked capacity to consent through illness, the written consent of the responsible next of kin was obtained. All clinical data and samples were anonymized.

### Patients and isolates

The Vietnamese isolates (total *n* = 136) included in this study were clinical isolates from the cerebrospinal fluid (CSF) of patients enrolled in a randomized controlled trial of antifungal therapy in HIV-infected patients (*n* = 98) between 2004 and 2011, and a prospective, descriptive study of HIV-uninfected patients with central nervous system (CNS) infections (*n* = 38) enrolled between 1998 and 2009.^7,18^ The Laotian isolates were from 81 patients with cryptococcal meningitis (67 HIV-infected, 8 HIV-uninfected, and 6 with unknown HIV status) consecutively admitted to Mahosot and associated hospitals in Vientiane, between 2003 and 2015.

### DNA extraction and restriction fragment length polymorphism (RFLP) analysis

Total genomic DNA was extracted from all organisms using the Masterpure Yeast DNA kit (Epicentre Biotechnologies, Madison, WI, USA) according to the manufacturer's instructions. Restriction fragment length polymorphism analysis (RFLP) of the orotidine monophosphate pyrophosphorylase (URA5) gene was used to confirm species/varietal status of all *C. neoformans* var. *grubii* isolates.^24^ Polymerase chain reaction (PCR) of the URA5 gene was conducted in a final volume of 50 μl. Each reaction contained 50 ng of DNA, 1X HotStart PCR buffer (NEB, USA), 0.2 mM each of dATP, dCTP, dGTP, and dTTP (Roche Diagnostics GmbH), 2 mM magnesium acetate, 0.5 U HotStart Taq DNA polymerase (NEB, USA), and 0.15 μM of each primer URA5 (5′- ATG TCC TCC CAA GCC CTC GAC TCC G - 3′) and S01 (5′- TTA AGA CCT CTG AAC ACC GTA CTC - 3′). PCR was performed in a Nexus SX1 thermal cycler (Eppendorf, USA) at 95°C for 15 min, followed by 35 cycles of 20 s denaturation at 94°C, 40 s annealing at 61°C, and 1 min extension at 72°C, followed by a final extension cycle for 3 min at 72°C. In sum, 10 μl of PCR products were double digested with Sau96I (0.2 U/μl) and HhaI (0.8 U/μl) for 3 h at 37^o^C, followed by a final 10 min incubation at 60^o^C. Restriction products are separated by 3% agarose gel electrophoresis at 100 V for 5 h. RFLP patterns were assigned visually by comparing them with the patterns obtained from the control strains (VNI-VNIV and VGI-VGIV; see Supplementary Table S1) kindly provided by Prof. Wieland Meyer, University of Sydney.

### Multilocus sequence typing (MLST)

Seven MLST loci (CAP59, GPD1, IGS1, LAC1, PLB1, SOD1, and URA5) were amplified and sequenced following the procedures of the International Society for Human and Animal Mycology (ISHAM) MLST consensus typing scheme for *C. neoformans* (http://mlst.mycologylab.org).^25^ Sequencing was performed using BigDye v3.1 Chemistry (Applied Biosystems, CA, USA) on an ABI 3130 Genetic Analyzer (Applied Biosystems, CA, USA). Both forward and reverse amplicons of each locus were sequenced. Consensus sequences were manually edited using ContigExpress and aligned in AlignX, implemented in VectorNTI Suite 7.0.^26^ A single Allele Type (AT) number was assigned to each of the seven loci by comparing consensus DNA sequences with the ISHAM database, resulting in a seven-number allelic profile for each isolate. The allelic profiles defined the corresponding STs.

MLST profiles and DNA sequences at each MLST loci for isolates from regions other than Laos and Vietnam were obtained from NCBI. For the global analysis, we used data reported by Simwami et al.,^21^ Mihara et al.,^14^ and Cogliati et al.^27^ included in Khayhan et al.,^17^ and from Chen et al.,^28^ Ferreira-Paim et al.,^29^ Beale et al.,^30^ and Dou et al.^31^

### Phylogenetic analyses

For global comparison, information on MLST genotypes, patient HIV status, and source of isolation for 1047 isolates from other countries were obtained from NCBI.^14,17,21,27,28^ These consisted of clinical and environmental *C. neoformans* var*. grubii* isolates from Southeast Asia (Indonesia, Thailand, Vietnam); East Asia (Japan, China, South Korea); South Asia (India), Middle East (Qatar, Kuwait); North America (USA); South America (Brazil, Argentina); Europe (Germany, Italy, France, Belgium); Australasia (Australia), and Africa (Botswana, Malawi, Tanzania, Uganda, Zaire, South Africa). Further details of the isolates and corresponding patient information are presented in supplementary Table S1.

First, we determined the best DNA substitution model for the concatenated dataset using MEGA v6.0.6.^32^ The Kimura two-parameters model with gamma distribution was selected as the best-fit model using the Bayesian information criteria (BIC) calculated with MEGA v6.0.6.^32^ To evaluate patterns of evolutionary descent among genotypes according to their source and geographic region, the allelic profile of the Vietnamese, Laos, and global data set were applied to the goeBURST algorithm in PHYLOVIZ 2.0 software available at http://www.phyloviz.net.^33,34^ A group founder was defined as the sequence type with the most number of linked single locus variants (SLV). The concept of a clonal complex (CC) was adopted when a SLV linkage with the founder ST was observed.

### Multidimensional (MDS) clustering

Genetic distance (Fst) and geographical distance were visualized by the nonmetric multidimensional scaling method. Fst were calculated from concatenated sequences of the seven MLST housekeeping genes using DnaSP v5.^35^ MDS calculation was performed using RStudio Version 0.98.1103 built with R-3.4.0 (https://www.rstudio.com and https://www.r-project.org). Network analysis and visualization was conducted using the R package igraph (version 1.1.2).

## Results

### MLST reveals dominance of ST4 and ST6 sequence types in Laos

**Figure 1.**
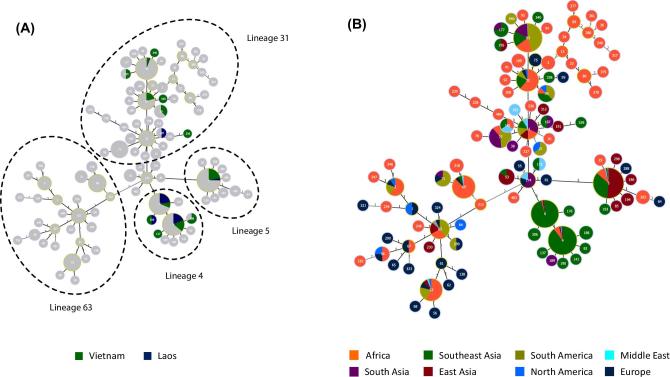
goeBurst analysis integrating the Laotian and Vietnamese *C. neoformans* var. *grubii* isolates into the major Asian clonal complex. Minimum spanning tree showing the major Asian clonal complexes (lineages). (A) Laos and Vietnam as part of the global population. The main subgroups of the major clonal complex are indicated by circles. (B) The major clonal complex broken down into geographical regions: East Asia (China and Japan), Southeast Asia (Vietnam, Laos, Thailand and Indonesia), South Asia (India), plus the reference groups Middle East (Qatar and Kuwait), Europe, North/South America and Africa. The number in the center of each circle indicates the ST of those isolates.

All 81 Laotian isolates belonged to the RFLP-defined VNI group. Among them, ST4 and ST6 accounted for over 40% each of the total number of isolates, and were 4-fold more prevalent than ST5; (ST4, *n* = 35 (43%); ST6, *n* = 33 (41%); ST5, *n* = 9 (11%), Fig. 1A, Table 1). Less than 3% of isolates from Laos (*n* = 2) were ST93. This contrasted with its adjacent neighbor, Vietnam (N = 136), where ST5 predominated (*n* = 66; 48%), followed by ST4 (*n* = 32; 24%), ST93 ( = 8; 6%), and ST6 (*n* = 12; 9%).

**Table 1.** Distribution of major Sequence Types (ST) in the Asia/Middle East region according to country of origin and HIV status.

	Sequence Type (ST)*															
Country HIV status	4	5	6	31	32	39	53	69	93	174	177	187	188	194	195	306
**China**		**104**		**1**			**5**		**1**					**2**	**1**	
HIV(−)		80		1			4		1					1		
HIV(+)		24					1							1	1	
**India**	**1**		**2**	**7**					**28**	**3**	**1**	**1**				
HIV(−)	1			4					6	2	1	1				
HIV(+)			2	3					22	1						
**Indonesia**	**8**		**10**					**2**	**16**		**3**					
HIV(−)											1					
HIV(+)	8		10					2	16		2					
**Japan**		**20**														
HIV(−)		20														
HIV(+)																
**Kuwait**	**1**	**2**						**1**	**1**	**1**						
HIV(−)		2						1		1						
HIV(+)	1								1							
**Laos**	**32**	**8**	**32**						**1**			**1**				**1**
HIV(−)	3	2	2						1							
HIV(+)	29	6	30									1				1
**Qatar**	**1**	**2**		**2**												
HIV(−)		1		2												
HIV(+)	1	1														
**Thailand**	**64**	**20**	**57**						**3**							
HIV(−)	2								1							
HIV(+)	62	20	57						2							
**Vietnam**	**32**	**66**	**12**		**7**	**3**			**8**				**2**		**1**	**1**
HIV(−)	3	31	1		2											1
HIV(+)	29	35	11		5	3			8				2		1	
**Total**	**139**	**222**	**113**	**10**	**7**	**3**	**5**	**3**	**58**	**4**	**4**	**2**	**2**	**2**	**2**	**2**

Numbers within cells are the number of isolates of that ST from that country. HIV, human immunodeficiency virus.

*Any minor sequence type with only one isolate was excluded from this table

### Correlation between infection with ST5 and HIV status

Isolates of ST5 have previously been reported to be associated with HIV-uninfected patients in East Asia and Vietnam.^7,14,18,19,36^ Most cases of cryptococcal meningitis in Laos were associated with HIV infection. The ST5 lineage accounted for just 9% (6 of 67) of infections in HIV-infected patients, suggesting a relatively low prevalence in the environment. Of eight cases of meningitis in Laos in HIV-uninfected patients, two were due to ST5 isolates (odds ratio 3.39 [95% confidence interval {CI}: 0.56 to 20.64], P *=* .18, Fisher exact test). HIV serostatus was not known for the remaining six patients.

To increase the power to test an association between ST5 isolates and HIV infection, we gathered previously described data from Asia, Europe, South America, and Africa on lineage and patient HIV serostatus.^17,23,28,30^ Clinical isolates with unknown HIV status were excluded from the analysis. Of 194 HIV uninfected patients, 136 (70%) were infected with ST5 isolates; of 791 HIV infected patients, 117 (15%) were infected with ST5 isolates (odds ratio 13.51 [95% CI: 9.38 to 19.45], *P* < .0001, Fisher exact test). There were 296 clinical or environmental ST5 isolates in the dataset; East and Southeast Asia accounted for the majority of these (*n* = 153 [51%] and *n* = 104 [35%], respectively). Here, the relative proportion of ST5 isolates from each country decreased along a clockwise arc from Northeast to Southwest: Japan (*n* = 36; 86%, 95% CI: [72–94]), China (*n* = 116; 86%, 95% CI: [80–92]), Vietnam (*n* = 66; 48%, 95% CI: [40–57]), Thailand (*n* = 29; 13%, 95% CI: [9–18]) and Laos (*n* = 9; 11%, 95% CI: [5–20]). ST5 accounted for 20% (*n* = 2) and 40% (*n* = 2) of the total number of isolates from Kuwait and Qatar in the Middle East, respectively.

The majority of cases of disease in apparently immunocompetent, HIV-uninfected patients were from East and Southeast Asia. ST5 has been reported from elsewhere, including Brazil (n = 3; 2%, 95% CI: [5–7]), South Africa (*n* = 27; 15%, 95% CI: [10–22]), Uganda (*n* = 1; 6%, 95% CI: [0–30]) and Botswana (*n* = 1; 0.7%, 95% CI: [0.0–3.7]), but in these regions disease in HIV-uninfected patients due to the type has rarely been reported.^28–30^

### GoeBurst analysis and geographical distribution

We used the previously published data to elucidate how the Laotian and Vietnamese populations fit in the wider global context.^17,20,28–30^ GoeBurst analysis placed *C. neoformans* var. *grubii* isolates from Vietnam and Laos, along with those from all other Asian, North/South American, Middle Eastern, and African countries in a clonal complex (Fig. 1A and B). The main clonal complex included three main goeBurst subgroups derived from ST174; subgroup 63 (ST63), subgroup 4 (ST4/ST6), subgroup 5 (ST5), and subgroup 31 (ST31/ST32/ST93) (Fig. 1A). Subgroup 63 was a double locus variant (DLV) from ST174 and was more distant to the other three subgroups. While most South African and Ugandan isolates were included in the main clonal complex, the bulk of isolates from Botswana were separated from the major clonal complex by at least 5 SLVs. ST174 was placed at the center of the clonal complex and was identified as a potential group founder of the clonal complex when analyzed by both parsimony (Fig. 1B) and maximum likelihood analyses (Fig. S1).

We observed an association between geographical location and STs (Fig. 1B). Most Asian isolates were included in subgroup 4 and subgroup 5, while isolates from other regions were widely distributed across the clonal complex. Subgroup 63 included European, North/South American, African, as well as a few East Asian isolates, but none from Southeast Asia. While subgroup 4 was reported from other continents, it was highly prevalent in Southeast Asia (*n* = 317, 66% of all Southeast Asian isolates (total N = 479)). Isolates from sub-group 5 had a wide distribution but in different proportions depending on the geographical regions: the Middle East (*n* = 4; 33% [regional total N = 15]), Africa (*n* = 33; 10% [regional total N = 346]), Europe (*n* = 5; 13% [regional total N = 39]), North America (*n* = 1; 11% [regional total N = 9]), South America (*n* = 3; 2% [regional total N = 133]), Southeast Asia (n = 105; 22% [regional total N = 479]) and most notably from East Asia (*n* = 158; 88% [regional total N = 180]). Similarly, subgroup 31 was also widely distributed but was most prevalent in South America (*n* = 110; 83% [regional total N = 133]) and South Asia (*n* = 53; 85% [regional total N = 62]). Other regions with subgroup 31 populations included Africa (*n* = 86; 25% [regional total N = 346]), the Middle East (*n* = 4; 27% [regional total N = 15]), North America (*n* = 3; 33% [regional total N = 9]), Europe (*n* = 6; 15% [regional total N = 39]). Subgroup 31 was much less common in East Asia (*n* = 6; 3% [regional total N = 180]) and Southeast Asia (*n* = 52; 11% [regional total N = 479]).

### Genetic distance between *C. neoformans* populations from different geographical regions

**Figure 2.**
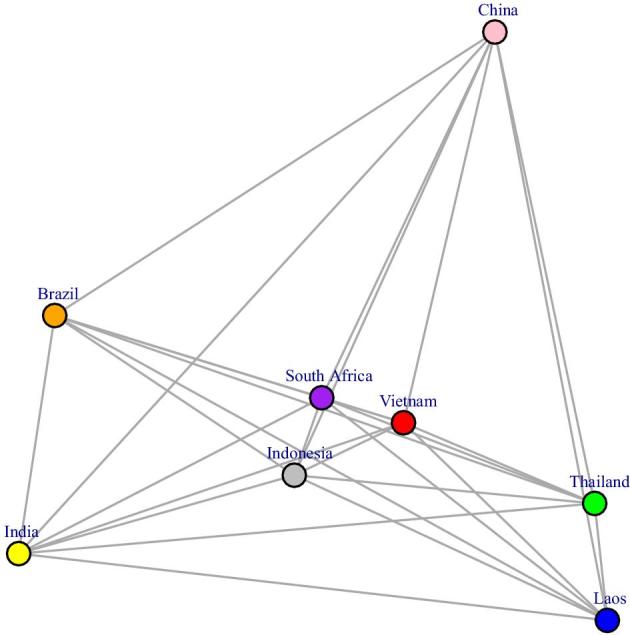
Mutidimensional scaling plot of genetic distances (Fst) of global *C. neoformans* var. *grubii* isolates from HIV-infected patients. Vietnam appeared to represent an intermediate population between that from Laos/Thailand and that from China. Among Southeast Asian populations, that of Indonesia is most similar with the Indian population, possibly due to the uniquely high proportion of ST93, a feature also shared by *C. neoformans* var. *grubii* from India and Brazil. This Figure is reproduced in color in the online version of *Medical Mycology*.

We employed the fixation index (*Fst*) to evaluate pairwise genetic differentiation between different geographical regions. Multidimensional scaling (MDS) was carried out to provide a 2-D visualization of the *Fst* distance matrix (Table 2 and Fig. 2). Botswana, being structurally highly diverse, is in stark contrast to other countries and therefore was removed from the analysis. Furthermore, due to the apparent propensity of ST5 towards HIV-uninfected patients, the *Fst* analysis only included isolates from HIV-infected patients from Laos, Thailand, Vietnam, Indonesia, China, India, South Africa. and Brazil. Very few isolates from Japan, Korea, Kuwait, or Qatar were known to be from HIV-infected patients. and thus isolates from these countries were also excluded from the analysis. In addition, isolates from any countries with fewer than 20 isolates were also excluded on the grounds that it was difficult to be sure they were representative of the country's wider *C. neoformans* var. *grubii* population.

**Table 2.** Genetic distance matrix (Fst) of *C. neoformans* var. *grubii* populations by country.

	Laos	Indonesia	Thailand	India	South Africa	Vietnam	Brazil	China
Laos	0	0.263	0.002	0.676	0.158	0.09	0.62	0.424
Indonesia		0	0.245	0.155	0.029	0.077	0.146	0.285
Thailand			0	0.605	0.205	0.095	0.632	0.28
India				0	0.112	0.304	0.016	0.635
South Africa					0	0.068	0.156	0.086
Vietnam						0	0.362	0.075
Brazil							0	0.495
China								0

The MDS plot revealed that the Indian *C. neoformans* var. *grubii* population was most closely related to the Brazilian population (*Fst* = 0.016), followed by the Indonesian population (*Fst* = 0.155) and was more distant to other Southeast Asian countries (Vietnam, Thailand, and Laos, *Fst* ranging from 0.304 to 0.676). Of the Southeast Asian populations, those from Laos and Thailand were most genetically close (*Fst* = 0.002); the Indonesian population was most closely related to the Vietnamese population (*Fst* = 0.077). The population from Vietnam, while being placed closer to Indonesia in the MDS analysis, appeared to be intermediate between China in East Asia (*Fst* = 0.075) and Laos/Thailand in Southeast Asia (*Fst* = 0.090 and *Fst* = 0.095, respectively). This is in spite of the fact that both Vietnam and Laos share northern borders with China (*Fst* = 0.424 between Laos and China). In fact, the Thai and Chinese populations appeared genetically closer than the Laos-China populations (*Fst* = 0.280).

## Discussion

The population structure of *C. neoformans* var. *grubii* has been described for a number of Asian countries,^17^ including Vietnam,^23^ Thailand,^10,21^ India,^37^ China,^36,38–40^ Japan,^14^ and Korea.^13^ However, data from Laos have been lacking, and Vietnamese data have not previously been placed into the global context. To address this, we typed 81 isolates from Laos and re-analyzed 136 previously published isolates from Vietnam using MLST. A strength of our data set is that it contains significant numbers of strains from both HIV-infected and uninfected patients. In contrast, reports from China and East Asian countries are dominated by clinical isolates from HIV-uninfected patients, while studies from Thailand and other Southeast Asian countries including Singapore^41^, Malaysia,^42^ and Indonesia^43^ present isolates predominantly from HIV-infected patients. The differences between the immune statuses of patients sampled in different countries complicate interpretation of the data but also mean that Vietnam is in a unique position to demonstrate the association between *C. neoformans* var. *grubii* ST5 and host immune phenotype.

In China, ST5 is the dominant sequence type, accounting for over 90% of infections.^8,19,31^ While it is present in both immunocompetent and immunocompromised patients, relatively few cases of HIV-associated disease have been reported.^31^ The reasons for this are unclear. While China has a relatively low prevalence of HIV (estimated at <0.1% of the adult population in 2015^44^), given the size of its population the few cases of HIV-associated disease reported may represent bias in study design, HIV testing or research interests. However, a recent paper has reported 35 cases of cryptococcosis in HIV infected people, of which 33 were ST5. Overall, they reported that 73 of 86 patients in their study were infected with ST5 isolates.^45^

In contrast to China, the *C. neoformans* var. *grubii* population from Thailand appears more diverse according to MLST, with 14 STs reported, among which ST4 and ST6 are most common, outnumbering ST5 infections 6 to 1.^10,17,21^ We found the population structure of isolates from Laos to be similar to that in Thailand, with ST4 and ST6 (both belonging to subgroup 4) accounting for the majority of disease. This dominance of a single sub-group in both Thailand/Laos and China contrasts with Vietnam, where ST5, ST6, and ST4, belonging to two separate sub-groups, are similarly prevalent. The Thai data are notable for the lack of cryptococcal meningitis cases reported from HIV-uninfected patients. It seems unlikely that this represents underreporting of non-HIV associated disease which is generally considered remarkable; rather, we hypothesize that this reflects a low prevalence of ST5 isolates in the environment, which is clearly associated with apparently immunocompetent patients elsewhere in Asia.^8,18,19,23,31^ If the ST5 lineage was common in Thailand and Laos, we would expect to detect this from reporting of disease in HIV infected patients; in Vietnam it is responsible for >35% of cases of cryptococcal meningitis in HIV-infected patients. Thus in Vietnam the *Cryptococcus* population seems to represents an intermediate population between Laos/Thailand to the West, and China to the Northeast, consistent with its geographical location.

Of note, Indonesia also appears to have two equally dominant burst groups among clinical isolates. ST4/6 isolates account for 44%, of human infections, with ST93, common in East Africa and also frequently reported from India, accounting for a further 44%.^17^

The differences in population structure we see moving from west to east are likely driven by ecological and/or human factors. Ecological differences between the regions could select for enrichment of particular STs if there are differences in the relative fitness of the STs in those niches. Southeast Asia is a geographically and ecologically diverse area known for its high species density.^46,47^ The south of the region has a hot tropical climate with dry and wet seasons; the north is temperate with hot summers and cold winters. The Annamite mountain range, covered in dense rain forests, rises to over 3000 meters and runs the length of Vietnam, forming a barrier separating Thailand, Laos, and Cambodia from Vietnam and southern China. There are also significant differences in elevation (for example, Chiang Mai, in Northern Thailand and the source of most published Thai data, and Vientiane in Northwestern Laos are at significantly higher elevation than Ho Chi Minh City and much of Southeastern China (170–300 meters versus 10–20 meters above sea level). Other than elevation and temperature, localized differences in soil biogeochemistry, environmental predators such as amoebae,^48^ and tree species may also be relevant. For example, Laos, Cambodia, Vietnam, and China are among Asian countries where there is significant contamination of groundwater with high levels of arsenite.^49,50^ Recently, it has been shown that ST5 *C. neoformans* var. *grubii* isolates possess tandem repeats of the arsenite efflux transporter (Arr3).^51^ The greater the number of these repeat elements the higher the resistance to toxic arsenic containing compounds.^51^ Differences in the distribution of this pollutant could correlate with the distribution of ST5. Other human activities that could influence ecology or human exposure to infection include irrigation, urbanization, and mining, and human migration and international trade links could influence dispersal of *C. neoformans* var. *grubii* lineages in Asia and elsewhere.

In addition to ecological factors, the varying prevalence of lineages in particular human populations could be a result of differential host susceptibility to particular lineages. Such human genetic factors have previously been associated with susceptibility to tuberculosis.^52^ However, the dominant ethnic group in Vietnam (Kinh, >80%) is genetically closer to ethnic groups in Northern Thailand than to the Han Chinese from Beijing or Southern China^53^ (also see Fig. S2). The differences between the Thai and Vietnamese *C. neoformans* var. *grubii* populations we demonstrated here suggest that the diversity of the pathogen population is not driven by host susceptibility. Nevertheless, it remains possible that subtle human genetic polymorphisms exist that result in differential susceptibility to different cryptococcal species/lineages.

Our observations augment the findings of Ferreira-Paim and co-workers who suggest that the populations of *C. neoformans* var*. grubii* found in many geographical regions across Asia and the Middle East arose from ST174.^29^ The ST174 expansion model is supported by goeBurst and maximum likelihood phylogeny analyses, which show it is the most parsimonious candidate as the group founder of subgroups 4, 5, and 31, which account for over 90% of the Southeast Asian isolates. We did not observe ST174 in either Vietnam or Laos, but it has been present in previously published datasets from South Asia and the Middle East, with three isolates from India and one isolate from Kuwait^17^ and has also been reported from Germany in a patient of Asian origin.^54^ The prevailing hypothesis with respect to the evolution of CN, suggests that the current circulating Asia population emerged “Out-of-Africa.”^5^ Our data neither confirm nor support this hypothesis, rather showing that the extant population of CN associated with human disease in Asia has descended from a single common ancestor. Given the age of *C. neoformans* as a species, and the fact that it is not primarily a human pathogen, nor is it spread from person to person, it seems somewhat unlikely that its global distribution is driven by migration of (latently or actively) infected humans. However, as described earlier, other human factors may be relevant. The emergence of the preponderance in most countries of strains of a single burst group on a background of greater diversity, suggests different fitness of different STs in particular ecological types.

Our study addressed the lack of knowledge of the molecular epidemiology of *C. neoformans* var. *grubii* from Laos and place previously generated data from Vietnam into a broader context. We found a change in predominant STs as one moves from western to eastern longitudes, that the Vietnamese *C. neoformans* var. *grubii* population appears intermediate between Thai and Chinese populations, and is unique in mainland Southeast Asia for having a high prevalence of two subgroups. Our data suggest that ST174 is the putative most recent common ancestor for the majority of *C. neoformans* var. *grubii* sequence types in Asia. The *C. neoformans* var*. grubii* population in Asia present in this study appeared mostly clonal, which is similar to previous reports.

Most studies focusing on VNI, including ours, are weakened in that while cryptococcosis results from the inhalation of infectious propagules from the environment, few environmentally sourced isolates have been typed and thus the true diversity of the species in any country may be underestimated. More efficient environmental isolation techniques and systematic sampling would allow us to understand the true species diversity by region and could improve understanding of the basis of cryptococcal virulence as well as bottleneck events leading to the rise of pathogenic lineages.

## Supplementary Material

Supplemental Files
