# Supplementary material for: Multilocus sequence typing of Cryptococcus neoformans var. grubii from Laos in a regional and global context
Source: Med Mycol. 2018 Oct 19;57(5):557–65. doi: 10.1093/mmy/myy105 (PMC6581559; doi:10.1093/mmy/myy105)
Supplement: Supplemental Files [file myy105_supplemental_files.zip › mm-2018-0146-File004.docx]

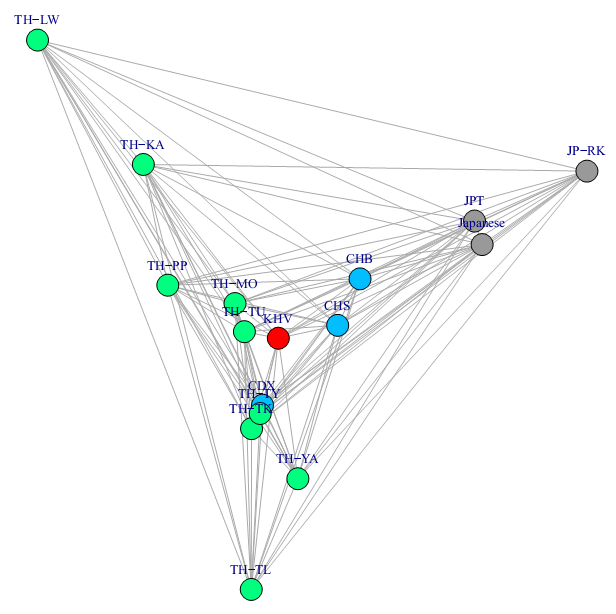


Thai

Vietnamese

Chinese

Japanese

**Figure S1 – Genetic distance between human populations from Japan, China, Vietnam and Thailand**

MDS plot showing SNPs data from WGS of different Asian populations from Vietnam, Japan, China and Thailand. Thai groups include TH-PP (Plang), TH-PL (Palong), TH-TK (Tai Kern), TH-TL (Tai Lue), TH-MO (Mon), TH-LW (Lawa), TH-KA (Karen), TH-YA (Yao), TH-TN (H’Tin), TH-MA (Mlabri), TH-TY (Tai Yong), TH-TU (Tai Yuan), TH-HM (Thai H’mong). Japanese groups include: JPT (Japanese from Tokyo), JP-RK (Ryukyuan) and Japanese (data from Human Genome Diversity Panel-CEPH). Chinese groups include Han Chinese from Beijing (CHB) and Southern China (CHS). Dai Chinese from Xishuangbanna was previously classied as a distant Southeast Asian population. Kinh Vietnamese from 2 most populous cities in Vietnam, Ho Chi Minh and Hanoi, were designated as KHV. Lu Dongsheng (Max Planck Independent Research Group on Population Genomics) compiled and provided human genome SNP data from the Human Genome Diversity Panel-CEPH, the HUGO Pan-Asian SNP Consortium and the 1000 Human Genome Project.

**Figure S2. Molecular Phylogenetic analysis by Maximum Likelihood method**

The evolutionary history was inferred by using the Maximum Likelihood method based on the Kimura 2-parameter model. The tree with the highest log likelihood (-6364.7916) is shown. The percentage of trees in which the associated taxa clustered together is shown above the branches. Bootstrap values over 50% were shown. ST174 was indicated by red arrow and ST4/ST5/ST6 by dark blue arrow. ST174 is suggested to be the most recent ancestor observable for ST4, ST5 and ST6 lineages.
