# Supplementary material for: Multilocus sequence typing of Cryptococcus neoformans var. grubii from Laos in a regional and global context
Source: Med Mycol. 2018 Oct 19;57(5):557–65. doi: 10.1093/mmy/myy105 (PMC6581559; doi:10.1093/mmy/myy105)
Supplement: Supplemental Files [file myy105_supplemental_files.zip › mm-2018-0146-File005.docx]

| **Table S1. Isolate details** | | | | | | | | | |
| --- | --- | --- | --- | --- | --- | --- | --- | --- | --- |
| **ID** | **Specimen** | **Specimen type** | **Location** | **Year** | **HIV** | **ST** | **goeBURST** | **Region** | **References** |
| 269 | Clinical | Clinical | Thailand | Unknown | Unknown | 6 | 4 | Southeast Asia | Khayhan et al., (2013) |
| 25_316 | CSF | Clinical | India | 2007 | Negative | 4 | 4 | South Asia | Khayhan et al., (2013) |
| CNS1553 | CSF | Clinical | Laos | 2015 | Negative | 4 | 4 | Southeast Asia | This study |
| FS4256/ CNS1444 | CSF | Clinical | Laos | Unknown | Negative | 4 | 4 | Southeast Asia | This study |
| UI_16721 | CSF | Clinical | Laos | Unknown | Negative | 4 | 4 | Southeast Asia | This study |
| CNS456 | CSF | Clinical | Laos | 2007 | Negative | 6 | 4 | Southeast Asia | This study |
| UI_16771 | CSF | Clinical | Laos | Unknown | Negative | 6 | 4 | Southeast Asia | This study |
| 1048 | Unknown | Clinical | Indonesia | Unknown | Unknown | 93 | 31 | Southeast Asia | Khayhan et al., (2013) |
| 1219 | Clinical | Clinical | Thailand | Unknown | Unknown | 6 | 4 | Southeast Asia | Khayhan et al., (2013) |
| 109A | Avian guano | Environmental | Thailand | Unknown | NA | 4 | 4 | Southeast Asia | Khayhan et al., (2013) |
| 109C | Avian guano | Environmental | Thailand | Unknown | NA | 4 | 4 | Southeast Asia | Khayhan et al., (2013) |
| 110C | Avian guano | Environmental | Thailand | Unknown | NA | 4 | 4 | Southeast Asia | Khayhan et al., (2013) |
| 110D | Avian guano | Environmental | Thailand | Unknown | NA | 4 | 4 | Southeast Asia | Khayhan et al., (2013) |
| 130C | Avian guano | Environmental | Thailand | Unknown | NA | 4 | 4 | Southeast Asia | Khayhan et al., (2013) |
| 3281 | Unknown | Clinical | Indonesia | Unknown | Unknown | 93 | 31 | Southeast Asia | Khayhan et al., (2013) |
| 9172 | CSF | Clinical | Japan | 1984 | Unknown | 5 | 5 | East Asia | Khayhan et al., (2013) |
| 9197 | Unknown | Clinical | Japan | 1989 | Unknown | 5 | 5 | East Asia | Khayhan et al., (2013) |
| 9198 | Unknown | Clinical | Japan | 1989 | Unknown | 5 | 5 | East Asia | Khayhan et al., (2013) |
| 96B | Avian guano | Environmental | Thailand | Unknown | NA | 4 | 4 | Southeast Asia | Khayhan et al., (2013) |
| 1111I_08 | Blood | Clinical | Thailand | Unknown | Negative | 4 | 4 | Southeast Asia | Khayhan et al., (2013) |
| D12 | Avian guano | Environmental | Thailand | 2000 | NA | 4 | 4 | Southeast Asia | Khayhan et al., (2013) |
| D14 | Avian guano | Environmental | Thailand | 2000 | NA | 4 | 4 | Southeast Asia | Khayhan et al., (2013) |
| D2 | Avian guano | Environmental | Thailand | 2000 | NA | 4 | 4 | Southeast Asia | Khayhan et al., (2013) |
| D27 | Avian guano | Environmental | Thailand | 2000 | NA | 4 | 4 | Southeast Asia | Khayhan et al., (2013) |
| D28 | Avian guano | Environmental | Thailand | 2000 | NA | 4 | 4 | Southeast Asia | Khayhan et al., (2013) |
| D3 | Avian guano | Environmental | Thailand | 2000 | NA | 4 | 4 | Southeast Asia | Khayhan et al., (2013) |
| D31 | Avian guano | Environmental | Thailand | 2000 | NA | 4 | 4 | Southeast Asia | Khayhan et al., (2013) |
| D43 | Avian guano | Environmental | Thailand | 2000 | NA | 4 | 4 | Southeast Asia | Khayhan et al., (2013) |
| D46 | Avian guano | Environmental | Thailand | 2000 | NA | 4 | 4 | Southeast Asia | Khayhan et al., (2013) |
| D69 | Avian guano | Environmental | Thailand | 2000 | NA | 4 | 4 | Southeast Asia | Khayhan et al., (2013) |
| D71 | Avian guano | Environmental | Thailand | 2000 | NA | 4 | 4 | Southeast Asia | Khayhan et al., (2013) |
| D73 | Avian guano | Environmental | Thailand | 2000 | NA | 4 | 4 | Southeast Asia | Khayhan et al., (2013) |
| 9199 | Unknown | Clinical | Japan | 1989 | Unknown | 5 | 5 | East Asia | Khayhan et al., (2013) |
| PG21 | Avian guano | Environmental | Thailand | 2000 | NA | 4 | 4 | Southeast Asia | Khayhan et al., (2013) |
| PG26 | Avian guano | Environmental | Thailand | 2000 | NA | 4 | 4 | Southeast Asia | Khayhan et al., (2013) |
| PG3 | Avian guano | Environmental | Thailand | 2000 | NA | 4 | 4 | Southeast Asia | Khayhan et al., (2013) |
| PG46 | Avian guano | Environmental | Thailand | 2000 | NA | 4 | 4 | Southeast Asia | Khayhan et al., (2013) |
| 2895I_08 | Blood | Clinical | Thailand | Unknown | Negative | 4 | 4 | Southeast Asia | Khayhan et al., (2013) |
| BK1 | CSF | Clinical | Vietnam |  | Positive | 4 | 4 | Southeast Asia | This study |
| BK111 | CSF | Clinical | Vietnam |  | Positive | 4 | 4 | Southeast Asia | This study |
| BK120 | CSF | Clinical | Vietnam |  | Positive | 4 | 4 | Southeast Asia | This study |
| 9204 | Unknown | Clinical | Japan | 1989 | Unknown | 5 | 5 | East Asia | Khayhan et al., (2013) |
| 05-0006 | Unknown | Clinical | Germany | 2005 | Positive | 4 | 4 | Europe | Sanchini et al. Med Microbiol Immunol. 2014,203(5):333-340 |
| 04-0059 | Unknown | Clinical | Germany | 2004 | Positive | 6 | 4 | Europe | Sanchini et al. Med Microbiol Immunol. 2014,203(5):333-340 |
| 25_372 | CSF | Clinical | India | 2009 | Positive | 6 | 4 | South Asia | Khayhan et al., (2013) |
| 25_373 | CSF | Clinical | India | 2009 | Positive | 6 | 4 | South Asia | Khayhan et al., (2013) |
| 268 | CSF | Clinical | Indonesia | 2006 | Positive | 4 | 4 | Southeast Asia | Khayhan et al., (2013) |
| 328 | CSF | Clinical | Indonesia | 2006 | Positive | 4 | 4 | Southeast Asia | Khayhan et al., (2013) |
| 544 | CSF | Clinical | Indonesia | 2006 | Positive | 4 | 4 | Southeast Asia | Khayhan et al., (2013) |
| 612 | CSF | Clinical | Indonesia | 2007 | Positive | 4 | 4 | Southeast Asia | Khayhan et al., (2013) |
| 778 | CSF | Clinical | Indonesia | 2007 | Positive | 4 | 4 | Southeast Asia | Khayhan et al., (2013) |
| 2597 | CSF | Clinical | Indonesia | 2006 | Positive | 4 | 4 | Southeast Asia | Khayhan et al., (2013) |
| 2606 | CSF | Clinical | Indonesia | 2005 | Positive | 4 | 4 | Southeast Asia | Khayhan et al., (2013) |
| Jakarta | CSF | Clinical | Indonesia | 2006 | Positive | 4 | 4 | Southeast Asia | Khayhan et al., (2013) |
| 267 | CSF | Clinical | Indonesia | 2006 | Positive | 6 | 4 | Southeast Asia | Khayhan et al., (2013) |
| 2339 | CSF | Clinical | Indonesia | 2006 | Positive | 6 | 4 | Southeast Asia | Khayhan et al., (2013) |
| 2594 | CSF | Clinical | Indonesia | 2006 | Positive | 6 | 4 | Southeast Asia | Khayhan et al., (2013) |
| 3187 | CSF | Clinical | Indonesia | 2006 | Positive | 6 | 4 | Southeast Asia | Khayhan et al., (2013) |
| Jakarta (H) | Blood | Clinical | Indonesia | 2006 | Positive | 6 | 4 | Southeast Asia | Khayhan et al., (2013) |
| Jakarta (KT) | Skin | Clinical | Indonesia | 2006 | Positive | 6 | 4 | Southeast Asia | Khayhan et al., (2013) |
| Jakarta (KT) | Skin | Clinical | Indonesia | 2006 | Positive | 6 | 4 | Southeast Asia | Khayhan et al., (2013) |
| Jakarta (P) | Blood | Clinical | Indonesia | 2006 | Positive | 6 | 4 | Southeast Asia | Khayhan et al., (2013) |
| Jakarta KLT | Skin | Clinical | Indonesia | 2006 | Positive | 6 | 4 | Southeast Asia | Khayhan et al., (2013) |
| Jakarta RTL | Skin | Clinical | Indonesia | 2006 | Positive | 6 | 4 | Southeast Asia | Khayhan et al., (2013) |
| 9205 | Unknown | Clinical | Japan | 1989 | Unknown | 5 | 5 | East Asia | Khayhan et al., (2013) |
| K30 | Blood | Clinical | Korea | #N/A | Positive | 4 | 4 | East Asia | Choi et al. FEMS Yeast Res. 2010,10(6):769–778 |
| 194_96 | Lym node biopsy | Clinical | Kuwait | 1996 | Positive | 4 | 4 | Middle East | Khayhan et al., (2013) |
| CNS1017 | CSF | Clinical | Laos | 2011 | Positive | 4 | 4 | Southeast Asia | This study |
| CNS1047 | CSF | Clinical | Laos | 2011 | Positive | 4 | 4 | Southeast Asia | This study |
| CNS1110 | CSF | Clinical | Laos | 2011 | Positive | 4 | 4 | Southeast Asia | This study |
| CNS1129 | CSF | Clinical | Laos | 2012 | Positive | 4 | 4 | Southeast Asia | This study |
| CNS1179 | CSF | Clinical | Laos | Unknown | Positive | 4 | 4 | Southeast Asia | This study |
| CNS1181 | CSF | Clinical | Laos | 2012 | Positive | 4 | 4 | Southeast Asia | This study |
| CNS133_2 | CSF | Clinical | Laos | 2003 | Positive | 4 | 4 | Southeast Asia | This study |
| CNS1379 | CSF | Clinical | Laos | 2013 | Positive | 4 | 4 | Southeast Asia | This study |
| CNS1394 | CSF | Clinical | Laos | 2013 | Positive | 4 | 4 | Southeast Asia | This study |
| CNS199 | CSF | Clinical | Laos | 2004 | Positive | 4 | 4 | Southeast Asia | This study |
| CNS289 | CSF | Clinical | Laos | 2005 | Positive | 4 | 4 | Southeast Asia | This study |
| CNS360 | CSF | Clinical | Laos | 2006 | Positive | 4 | 4 | Southeast Asia | This study |
| CNS365 | CSF | Clinical | Laos | 2005 | Positive | 4 | 4 | Southeast Asia | This study |
| CNS447 | CSF | Clinical | Laos | 2006 | Positive | 4 | 4 | Southeast Asia | This study |
| CNS625 | CSF | Clinical | Laos | 2008 | Positive | 4 | 4 | Southeast Asia | This study |
| CNS681 | CSF | Clinical | Laos | 2008 | Positive | 4 | 4 | Southeast Asia | This study |
| CNS806 | CSF | Clinical | Laos | 2009 | Positive | 4 | 4 | Southeast Asia | This study |
| CNS814 | CSF | Clinical | Laos | 2009 | Positive | 4 | 4 | Southeast Asia | This study |
| CNS897 | CSF | Clinical | Laos | 2010 | Positive | 4 | 4 | Southeast Asia | This study |
| CNS929 | CSF | Clinical | Laos | 2010 | Positive | 4 | 4 | Southeast Asia | This study |
| CNS955 | CSF | Clinical | Laos | 2010 | Positive | 4 | 4 | Southeast Asia | This study |
| CNS978 | CSF | Clinical | Laos | 2010 | Positive | 4 | 4 | Southeast Asia | This study |
| CNS999 | CSF | Clinical | Laos | 2011 | Positive | 4 | 4 | Southeast Asia | This study |
| HC_ST2296 | CSF | Clinical | Laos | Unknown | Positive | 4 | 4 | Southeast Asia | This study |
| HC_ST352 | CSF | Clinical | Laos | Unknown | Positive | 4 | 4 | Southeast Asia | This study |
| UI_11199 | CSF | Clinical | Laos | Unknown | Positive | 4 | 4 | Southeast Asia | This study |
| UI_29674 | CSF | Clinical | Laos | Unknown | Positive | 4 | 4 | Southeast Asia | This study |
| UI31647_2 | CSF | Clinical | Laos | Unknown | Positive | 4 | 4 | Southeast Asia | This study |
| UI32312 | CSF | Clinical | Laos | Unknown | Positive | 4 | 4 | Southeast Asia | This study |
| 9213 | Unknown | Clinical | Japan | 1989 | Unknown | 5 | 5 | East Asia | Khayhan et al., (2013) |
| 9217 | Unknown | Clinical | Japan | 1995 | Unknown | 5 | 5 | East Asia | Khayhan et al., (2013) |
| 44 | Avian guano | Environmental | Thailand | Unknown | NA | 6 | 4 | Southeast Asia | Khayhan et al., (2013) |
| 11109 | Clinical | Clinical | Thailand | Unknown | Unknown | 4 | 4 | Southeast Asia | Khayhan et al., (2013) |
| 11112 | Clinical | Clinical | Thailand | Unknown | Unknown | 4 | 4 | Southeast Asia | Khayhan et al., (2013) |
| 04-0106 | Unknown | Clinical | Germany | 2004 | Unknown | 5 | 5 | Europe | Sanchini et al. Med Microbiol Immunol. 2014,203(5):333-340 |
| 1_488 | Clinical | Clinical | Thailand | Unknown | Unknown | 5 | 5 | Southeast Asia | Khayhan et al., (2013) |
| 1_489 | Clinical | Clinical | Thailand | Unknown | Unknown | 5 | 5 | Southeast Asia | Khayhan et al., (2013) |
| 2551_07 | Avian guano | Environmental | Thailand | Unknown | NA | 6 | 4 | Southeast Asia | Khayhan et al., (2013) |
| 1_587 | Clinical | Clinical | Thailand | Unknown | Unknown | 6 | 4 | Southeast Asia | Khayhan et al., (2013) |
| 1_588 | Clinical | Clinical | Thailand | Unknown | Unknown | 6 | 4 | Southeast Asia | Khayhan et al., (2013) |
| 1_846 | Clinical | Clinical | Thailand | Unknown | Unknown | 6 | 4 | Southeast Asia | Khayhan et al., (2013) |
| 10-0484 | Unknown | Clinical | Germany | 2010 | Unknown | 58 | 63 | Europe | Sanchini et al. Med Microbiol Immunol. 2014,203(5):333-340 |
| D1 | Avian guano | Environmental | Thailand | 2000 | NA | 6 | 4 | Southeast Asia | Khayhan et al., (2013) |
| D18 | Avian guano | Environmental | Thailand | 2000 | NA | 6 | 4 | Southeast Asia | Khayhan et al., (2013) |
| D21 | Avian guano | Environmental | Thailand | 2000 | NA | 6 | 4 | Southeast Asia | Khayhan et al., (2013) |
| D22 | Avian guano | Environmental | Thailand | 2000 | NA | 6 | 4 | Southeast Asia | Khayhan et al., (2013) |
| D26 | Avian guano | Environmental | Thailand | 2000 | NA | 6 | 4 | Southeast Asia | Khayhan et al., (2013) |
| D30 | Avian guano | Environmental | Thailand | 2000 | NA | 6 | 4 | Southeast Asia | Khayhan et al., (2013) |
| D33 | Avian guano | Environmental | Thailand | 2000 | NA | 6 | 4 | Southeast Asia | Khayhan et al., (2013) |
| D34 | Avian guano | Environmental | Thailand | 2000 | NA | 6 | 4 | Southeast Asia | Khayhan et al., (2013) |
| D35 | Avian guano | Environmental | Thailand | 2000 | NA | 6 | 4 | Southeast Asia | Khayhan et al., (2013) |
| D36 | Avian guano | Environmental | Thailand | 2000 | NA | 6 | 4 | Southeast Asia | Khayhan et al., (2013) |
| D41 | Avian guano | Environmental | Thailand | 2000 | NA | 6 | 4 | Southeast Asia | Khayhan et al., (2013) |
| D64 | Avian guano | Environmental | Thailand | 2000 | NA | 6 | 4 | Southeast Asia | Khayhan et al., (2013) |
| D76 | Avian guano | Environmental | Thailand | 2000 | NA | 6 | 4 | Southeast Asia | Khayhan et al., (2013) |
| PG1 | Avian guano | Environmental | Thailand | 2000 | NA | 6 | 4 | Southeast Asia | Khayhan et al., (2013) |
| PG2 | Avian guano | Environmental | Thailand | 2000 | NA | 6 | 4 | Southeast Asia | Khayhan et al., (2013) |
| PG32 | Avian guano | Environmental | Thailand | 2000 | NA | 6 | 4 | Southeast Asia | Khayhan et al., (2013) |
| 04CN30_001/ UI24618/ CNS1285 | CSF | Clinical | Laos | Unknown | Positive | 6 | 4 | Southeast Asia | This study |
| 04CN30_008 / UI21923 / CNS1143_2 | CSF | Clinical | Laos | Unknown | Positive | 6 | 4 | Southeast Asia | This study |
| CNS_ST1930 | CSF | Clinical | Laos | Unknown | Positive | 6 | 4 | Southeast Asia | This study |
| CNS1026 | CSF | Clinical | Laos | 2011 | Positive | 6 | 4 | Southeast Asia | This study |
| CNS1085 | CSF | Clinical | Laos | 2011 | Positive | 6 | 4 | Southeast Asia | This study |
| CNS1087 | CSF | Clinical | Laos | 2011 | Positive | 6 | 4 | Southeast Asia | This study |
| CNS1089 | CSF | Clinical | Laos | 2011 | Positive | 6 | 4 | Southeast Asia | This study |
| CNS1125 | CSF | Clinical | Laos | 2012 | Positive | 6 | 4 | Southeast Asia | This study |
| CNS1156 | CSF | Clinical | Laos | 2012 | Positive | 6 | 4 | Southeast Asia | This study |
| CNS1285 | CSF | Clinical | Laos | 2013 | Positive | 6 | 4 | Southeast Asia | This study |
| CNS1302 | CSF | Clinical | Laos | 2013 | Positive | 6 | 4 | Southeast Asia | This study |
| CNS1385 | CSF | Clinical | Laos | 2013 | Positive | 6 | 4 | Southeast Asia | This study |
| CNS1403 | CSF | Clinical | Laos | 2014 | Positive | 6 | 4 | Southeast Asia | This study |
| CNS1413 | CSF | Clinical | Laos | 2014 | Positive | 6 | 4 | Southeast Asia | This study |
| CNS1419 | CSF | Clinical | Laos | Unknown | Positive | 6 | 4 | Southeast Asia | This study |
| CNS1420 | CSF | Clinical | Laos | 2014 | Positive | 6 | 4 | Southeast Asia | This study |
| CNS1453 | CSF | Clinical | Laos | 2014 | Positive | 6 | 4 | Southeast Asia | This study |
| CNS1474 | CSF | Clinical | Laos | 2014 | Positive | 6 | 4 | Southeast Asia | This study |
| CNS1589 | CSF | Clinical | Laos | 2015 | Positive | 6 | 4 | Southeast Asia | This study |
| CNS198 | CSF | Clinical | Laos | 2004 | Positive | 6 | 4 | Southeast Asia | This study |
| CNS445 | CSF | Clinical | Laos | 2006 | Positive | 6 | 4 | Southeast Asia | This study |
| CNS569 | CSF | Clinical | Laos | 2007 | Positive | 6 | 4 | Southeast Asia | This study |
| CNS573 | CSF | Clinical | Laos | 2007 | Positive | 6 | 4 | Southeast Asia | This study |
| CNS863 | CSF | Clinical | Laos | 2010 | Positive | 6 | 4 | Southeast Asia | This study |
| CNS984 | CSF | Clinical | Laos | 2010 | Positive | 6 | 4 | Southeast Asia | This study |
| ST1931 | CSF | Clinical | Laos | Unknown | Positive | 6 | 4 | Southeast Asia | This study |
| UI_12808 | CSF | Clinical | Laos | Unknown | Positive | 6 | 4 | Southeast Asia | This study |
| UI_14167 | CSF | Clinical | Laos | Unknown | Positive | 6 | 4 | Southeast Asia | This study |
| UI_14344 | CSF | Clinical | Laos | Unknown | Positive | 6 | 4 | Southeast Asia | This study |
| UI_21616 | CSF | Clinical | Laos | Unknown | Positive | 6 | 4 | Southeast Asia | This study |
| CNS626 | CSF | Clinical | Laos | 2008 | Positive | 306 | 4 | Southeast Asia | This study |
| mal 104 | Blood | Clinical | Malawi | #N/A | Positive | 4 | 4 | Africa | Litvintseva et al. Genetics. 2006,172(4):2223–2238 |
| 1608000894 | CSF | Clinical | Qatar | 2008 | Positive | 4 | 4 | Middle East | Khayhan et al., (2013) |
| 103 | CSF | Clinical | South Africa |  | Positive | 4 | 4 | Africa | Beale MA et al, Plos NTD,2015 |
| 113 | CSF | Clinical | South Africa |  | Positive | 4 | 4 | Africa | Beale MA et al, Plos NTD,2015 |
| 130 | CSF | Clinical | South Africa |  | Positive | 4 | 4 | Africa | Beale MA et al, Plos NTD,2015 |
| 134 | CSF | Clinical | South Africa |  | Positive | 4 | 4 | Africa | Beale MA et al, Plos NTD,2015 |
| 148 | CSF | Clinical | South Africa |  | Positive | 4 | 4 | Africa | Beale MA et al, Plos NTD,2015 |
| 206 | CSF | Clinical | South Africa |  | Positive | 4 | 4 | Africa | Beale MA et al, Plos NTD,2015 |
| 205 | CSF | Clinical | South Africa |  | Positive | 4 | 4 | Africa | Beale MA et al, Plos NTD,2015 |
| 264 | CSF | Clinical | South Africa |  | Positive | 4 | 4 | Africa | Beale MA et al, Plos NTD,2015 |
| BK14 | CSF | Clinical | Vietnam |  | Positive | 4 | 4 | Southeast Asia | This study |
| 313 | CSF | Clinical | South Africa |  | Positive | 4 | 4 | Africa | Beale MA et al, Plos NTD,2015 |
| 321 | CSF | Clinical | South Africa |  | Positive | 6 | 4 | Africa | Beale MA et al, Plos NTD,2015 |
| Tn148 | Blood | Clinical | Tanzania | #N/A | Positive | 4 | 4 | Africa | Litvintseva et al. Genetics. 2006,172(4):2223–2238 |
| 20662_07 | Blood | Clinical | Thailand | Unknown | Positive | 4 | 4 | Southeast Asia | Khayhan et al., (2013) |
| 28170_07 | CSF | Clinical | Thailand | 2007 | Positive | 4 | 4 | Southeast Asia | Khayhan et al., (2013) |
| 4500_07 | Blood | Clinical | Thailand | Unknown | Positive | 4 | 4 | Southeast Asia | Khayhan et al., (2013) |
| 50NC2 | CSF | Clinical | Thailand | 2003 | Positive | 4 | 4 | Southeast Asia | Khayhan et al., (2013) |
| 50NC5 | CSF | Clinical | Thailand | 2003 | Positive | 4 | 4 | Southeast Asia | Khayhan et al., (2013) |
| CM10 | CSF | Clinical | Thailand | 2014 | Positive | 4 | 4 | Southeast Asia | Khayhan et al., (2013) |
| CM11 | CSF | Clinical | Thailand | 2015 | Positive | 4 | 4 | Southeast Asia | Khayhan et al., (2013) |
| CM14 | CSF | Clinical | Thailand | Unknown | Positive | 4 | 4 | Southeast Asia | Khayhan et al., (2013) |
| CM15 | CSF | Clinical | Thailand |  | Positive | 4 | 4 | Southeast Asia | Khayhan et al., (2013) |
| CM16 | CSF | Clinical | Thailand |  | Positive | 4 | 4 | Southeast Asia | Khayhan et al., (2013) |
| CM2 | CSF | Clinical | Thailand |  | Positive | 4 | 4 | Southeast Asia | Khayhan et al., (2013) |
| CM20 | CSF | Clinical | Thailand |  | Positive | 4 | 4 | Southeast Asia | Khayhan et al., (2013) |
| CM24 | CSF | Clinical | Thailand |  | Positive | 4 | 4 | Southeast Asia | Khayhan et al., (2013) |
| CM27 | CSF | Clinical | Thailand |  | Positive | 4 | 4 | Southeast Asia | Khayhan et al., (2013) |
| CM28 | CSF | Clinical | Thailand |  | Positive | 4 | 4 | Southeast Asia | Khayhan et al., (2013) |
| CM29 | CSF | Clinical | Thailand |  | Positive | 4 | 4 | Southeast Asia | Khayhan et al., (2013) |
| CM3 | CSF | Clinical | Thailand |  | Positive | 4 | 4 | Southeast Asia | Khayhan et al., (2013) |
| CM32 | CSF | Clinical | Thailand |  | Positive | 4 | 4 | Southeast Asia | Khayhan et al., (2013) |
| CM34 | CSF | Clinical | Thailand |  | Positive | 4 | 4 | Southeast Asia | Khayhan et al., (2013) |
| CM36 | CSF | Clinical | Thailand |  | Positive | 4 | 4 | Southeast Asia | Khayhan et al., (2013) |
| CM4 | CSF | Clinical | Thailand |  | Positive | 4 | 4 | Southeast Asia | Khayhan et al., (2013) |
| CM45 | CSF | Clinical | Thailand |  | Positive | 4 | 4 | Southeast Asia | Khayhan et al., (2013) |
| CM5 | CSF | Clinical | Thailand |  | Positive | 4 | 4 | Southeast Asia | Khayhan et al., (2013) |
| CM50 | CSF | Clinical | Thailand |  | Positive | 4 | 4 | Southeast Asia | Khayhan et al., (2013) |
| CM52 | CSF | Clinical | Thailand |  | Positive | 4 | 4 | Southeast Asia | Khayhan et al., (2013) |
| CM60 | CSF | Clinical | Thailand |  | Positive | 4 | 4 | Southeast Asia | Khayhan et al., (2013) |
| CM64 | CSF | Clinical | Thailand |  | Positive | 4 | 4 | Southeast Asia | Khayhan et al., (2013) |
| CN49008 | CSF | Clinical | Thailand | 2007 | Positive | 4 | 4 | Southeast Asia | Khayhan et al., (2013) |
| CN4902 | CSF | Clinical | Thailand | 2006 | Positive | 4 | 4 | Southeast Asia | Khayhan et al., (2013) |
| CN4904 | CSF | Clinical | Thailand | 2006 | Positive | 4 | 4 | Southeast Asia | Khayhan et al., (2013) |
| CN4905 | CSF | Clinical | Thailand | 2006 | Positive | 4 | 4 | Southeast Asia | Khayhan et al., (2013) |
| CN4907 | CSF | Clinical | Thailand | 2006 | Positive | 4 | 4 | Southeast Asia | Khayhan et al., (2013) |
| CN4909 | CSF | Clinical | Thailand | 2006 | Positive | 4 | 4 | Southeast Asia | Khayhan et al., (2013) |
| CN4914 | CSF | Clinical | Thailand | 2006 | Positive | 4 | 4 | Southeast Asia | Khayhan et al., (2013) |
| CN4915 | CSF | Clinical | Thailand | 2006 | Positive | 4 | 4 | Southeast Asia | Khayhan et al., (2013) |
| CN4927 | CSF | Clinical | Thailand | 2006 | Positive | 4 | 4 | Southeast Asia | Khayhan et al., (2013) |
| CN4931 | CSF | Clinical | Thailand | 2006 | Positive | 4 | 4 | Southeast Asia | Khayhan et al., (2013) |
| CN4932 | CSF | Clinical | Thailand | 2006 | Positive | 4 | 4 | Southeast Asia | Khayhan et al., (2013) |
| CN4933 | CSF | Clinical | Thailand | 2006 | Positive | 4 | 4 | Southeast Asia | Khayhan et al., (2013) |
| CN4934 | CSF | Clinical | Thailand | 2006 | Positive | 4 | 4 | Southeast Asia | Khayhan et al., (2013) |
| CN4936 | CSF | Clinical | Thailand | 2006 | Positive | 4 | 4 | Southeast Asia | Khayhan et al., (2013) |
| CN4937 | CSF | Clinical | Thailand | 2006 | Positive | 4 | 4 | Southeast Asia | Khayhan et al., (2013) |
| CN4938 | CSF | Clinical | Thailand | 2006 | Positive | 4 | 4 | Southeast Asia | Khayhan et al., (2013) |
| CN4949 | CSF | Clinical | Thailand | 2007 | Positive | 4 | 4 | Southeast Asia | Khayhan et al., (2013) |
| CN4950 | CSF | Clinical | Thailand | 2007 | Positive | 4 | 4 | Southeast Asia | Khayhan et al., (2013) |
| 25_14 | Clinical | Clinical | India | Unknown | Unknown | 77 | 31 | South Asia | Khayhan et al., (2013) |
| CN4952 | CSF | Clinical | Thailand | 2010 | Positive | 4 | 4 | Southeast Asia | Khayhan et al., (2013) |
| CN4954 | CSF | Clinical | Thailand | 2007 | Positive | 4 | 4 | Southeast Asia | Khayhan et al., (2013) |
| CN4955 | Bronchoalveolar lavage | Clinical | Thailand | 2007 | Positive | 4 | 4 | Southeast Asia | Khayhan et al., (2013) |
| CN4956 | CSF | Clinical | Thailand | 2007 | Positive | 4 | 4 | Southeast Asia | Khayhan et al., (2013) |
| CN4957 | CSF | Clinical | Thailand | Unknown | Positive | 4 | 4 | Southeast Asia | Khayhan et al., (2013) |
| CN4968 | CSF | Clinical | Thailand | 2007 | Positive | 4 | 4 | Southeast Asia | Khayhan et al., (2013) |
| CN4970 | CSF | Clinical | Thailand | 2007 | Positive | 4 | 4 | Southeast Asia | Khayhan et al., (2013) |
| CN5001 | CSF | Clinical | Thailand | 2007 | Positive | 4 | 4 | Southeast Asia | Khayhan et al., (2013) |
| CN5002 | Blood | Clinical | Thailand | 2007 | Positive | 4 | 4 | Southeast Asia | Khayhan et al., (2013) |
| CN5003 | Blood | Clinical | Thailand | 2007 | Positive | 4 | 4 | Southeast Asia | Khayhan et al., (2013) |
| CN5005 | Blood | Clinical | Thailand | 2007 | Positive | 4 | 4 | Southeast Asia | Khayhan et al., (2013) |
| CN5009 | Blood | Clinical | Thailand | 2007 | Positive | 4 | 4 | Southeast Asia | Khayhan et al., (2013) |
| CN5011 | Blood | Clinical | Thailand | 2007 | Positive | 4 | 4 | Southeast Asia | Khayhan et al., (2013) |
| CN5013 | CSF | Clinical | Thailand | 2007 | Positive | 4 | 4 | Southeast Asia | Khayhan et al., (2013) |
| CN5014 | Blood | Clinical | Thailand | 2007 | Positive | 4 | 4 | Southeast Asia | Khayhan et al., (2013) |
| CN5017 | CSF | Clinical | Thailand | 2007 | Positive | 4 | 4 | Southeast Asia | Khayhan et al., (2013) |
| CN5019 | Blood | Clinical | Thailand | 2008 | Positive | 4 | 4 | Southeast Asia | Khayhan et al., (2013) |
| 2461_07 | CSF | Clinical | Thailand | Unknown | Positive | 6 | 4 | Southeast Asia | Khayhan et al., (2013) |
| 2550II_07 | Blood | Clinical | Thailand | Unknown | Positive | 6 | 4 | Southeast Asia | Khayhan et al., (2013) |
| 2551_07_CM | CSF | Clinical | Thailand | Unknown | Positive | 6 | 4 | Southeast Asia | Khayhan et al., (2013) |
| CM1 | CSF | Clinical | Thailand | 2002 | Positive | 6 | 4 | Southeast Asia | Khayhan et al., (2013) |
| CM12 | CSF | Clinical | Thailand | 2002 | Positive | 6 | 4 | Southeast Asia | Khayhan et al., (2013) |
| CM13 | CSF | Clinical | Thailand | 2002 | Positive | 6 | 4 | Southeast Asia | Khayhan et al., (2013) |
| CM17 | CSF | Clinical | Thailand | 2002 | Positive | 6 | 4 | Southeast Asia | Khayhan et al., (2013) |
| CM18 | CSF | Clinical | Thailand | 2002 | Positive | 6 | 4 | Southeast Asia | Khayhan et al., (2013) |
| CM22 | CSF | Clinical | Thailand | 2002 | Positive | 6 | 4 | Southeast Asia | Khayhan et al., (2013) |
| CM23 | CSF | Clinical | Thailand | 2002 | Positive | 6 | 4 | Southeast Asia | Khayhan et al., (2013) |
| CM25 | CSF | Clinical | Thailand | 2002 | Positive | 6 | 4 | Southeast Asia | Khayhan et al., (2013) |
| CM26 | CSF | Clinical | Thailand | 2002 | Positive | 6 | 4 | Southeast Asia | Khayhan et al., (2013) |
| CM33 | CSF | Clinical | Thailand | 2002 | Positive | 6 | 4 | Southeast Asia | Khayhan et al., (2013) |
| CM37 | CSF | Clinical | Thailand | 2002 | Positive | 6 | 4 | Southeast Asia | Khayhan et al., (2013) |
| CM38 | CSF | Clinical | Thailand | 2002 | Positive | 6 | 4 | Southeast Asia | Khayhan et al., (2013) |
| CM39 | CSF | Clinical | Thailand | 2002 | Positive | 6 | 4 | Southeast Asia | Khayhan et al., (2013) |
| CM40 | CSF | Clinical | Thailand | 2002 | Positive | 6 | 4 | Southeast Asia | Khayhan et al., (2013) |
| CM41 | CSF | Clinical | Thailand | 2002 | Positive | 6 | 4 | Southeast Asia | Khayhan et al., (2013) |
| CM42 | CSF | Clinical | Thailand | 2002 | Positive | 6 | 4 | Southeast Asia | Khayhan et al., (2013) |
| CM43 | CSF | Clinical | Thailand | 2002 | Positive | 6 | 4 | Southeast Asia | Khayhan et al., (2013) |
| CM44 | CSF | Clinical | Thailand | 2002 | Positive | 6 | 4 | Southeast Asia | Khayhan et al., (2013) |
| CM46 | CSF | Clinical | Thailand | 2002 | Positive | 6 | 4 | Southeast Asia | Khayhan et al., (2013) |
| CM47 | CSF | Clinical | Thailand | 2002 | Positive | 6 | 4 | Southeast Asia | Khayhan et al., (2013) |
| CM48 | CSF | Clinical | Thailand | 2002 | Positive | 6 | 4 | Southeast Asia | Khayhan et al., (2013) |
| CM49 | CSF | Clinical | Thailand | 2002 | Positive | 6 | 4 | Southeast Asia | Khayhan et al., (2013) |
| CM51 | CSF | Clinical | Thailand | 2002 | Positive | 6 | 4 | Southeast Asia | Khayhan et al., (2013) |
| CM55 | CSF | Clinical | Thailand | 2002 | Positive | 6 | 4 | Southeast Asia | Khayhan et al., (2013) |
| CM56 | CSF | Clinical | Thailand | 2002 | Positive | 6 | 4 | Southeast Asia | Khayhan et al., (2013) |
| CM57 | CSF | Clinical | Thailand | 2002 | Positive | 6 | 4 | Southeast Asia | Khayhan et al., (2013) |
| CM58 | CSF | Clinical | Thailand | 2002 | Positive | 6 | 4 | Southeast Asia | Khayhan et al., (2013) |
| CM59 | CSF | Clinical | Thailand | 2002 | Positive | 6 | 4 | Southeast Asia | Khayhan et al., (2013) |
| CM6 | CSF | Clinical | Thailand | 2002 | Positive | 6 | 4 | Southeast Asia | Khayhan et al., (2013) |
| CM61 | CSF | Clinical | Thailand | 2002 | Positive | 6 | 4 | Southeast Asia | Khayhan et al., (2013) |
| CM63 | CSF | Clinical | Thailand | 2002 | Positive | 6 | 4 | Southeast Asia | Khayhan et al., (2013) |
| CM7 | CSF | Clinical | Thailand | 2002 | Positive | 6 | 4 | Southeast Asia | Khayhan et al., (2013) |
| CM8 | CSF | Clinical | Thailand | 2002 | Positive | 6 | 4 | Southeast Asia | Khayhan et al., (2013) |
| CN49005 | CSF | Clinical | Thailand | 2007 | Positive | 6 | 4 | Southeast Asia | Khayhan et al., (2013) |
| CN4901 | CSF | Clinical | Thailand | 2006 | Positive | 6 | 4 | Southeast Asia | Khayhan et al., (2013) |
| CN4903 | CSF | Clinical | Thailand | 2006 | Positive | 6 | 4 | Southeast Asia | Khayhan et al., (2013) |
| CN4917 | CSF | Clinical | Thailand | 2006 | Positive | 6 | 4 | Southeast Asia | Khayhan et al., (2013) |
| CN4918 | CSF | Clinical | Thailand | 2006 | Positive | 6 | 4 | Southeast Asia | Khayhan et al., (2013) |
| CN4919 | CSF | Clinical | Thailand | 2006 | Positive | 6 | 4 | Southeast Asia | Khayhan et al., (2013) |
| CN4926 | CSF | Clinical | Thailand | 2006 | Positive | 6 | 4 | Southeast Asia | Khayhan et al., (2013) |
| CN4940 | CSF | Clinical | Thailand | 2006 | Positive | 6 | 4 | Southeast Asia | Khayhan et al., (2013) |
| CN4941 | CSF | Clinical | Thailand | 2006 | Positive | 6 | 4 | Southeast Asia | Khayhan et al., (2013) |
| CN4942 | CSF | Clinical | Thailand | 2006 | Positive | 6 | 4 | Southeast Asia | Khayhan et al., (2013) |
| CN4943 | CSF | Clinical | Thailand | 2006 | Positive | 6 | 4 | Southeast Asia | Khayhan et al., (2013) |
| CN4944 | CSF | Clinical | Thailand | 2006 | Positive | 6 | 4 | Southeast Asia | Khayhan et al., (2013) |
| CN4945 | CSF | Clinical | Thailand | 2006 | Positive | 6 | 4 | Southeast Asia | Khayhan et al., (2013) |
| CN4947 | CSF | Clinical | Thailand | 2006 | Positive | 6 | 4 | Southeast Asia | Khayhan et al., (2013) |
| CN4964 | CSF | Clinical | Thailand | 2007 | Positive | 6 | 4 | Southeast Asia | Khayhan et al., (2013) |
| CN4987 | CSF | Clinical | Thailand | 2007 | Positive | 6 | 4 | Southeast Asia | Khayhan et al., (2013) |
| CN4988 | CSF | Clinical | Thailand | 2007 | Positive | 6 | 4 | Southeast Asia | Khayhan et al., (2013) |
| CN4989 | CSF | Clinical | Thailand | 2007 | Positive | 6 | 4 | Southeast Asia | Khayhan et al., (2013) |
| CN4995 | CSF | Clinical | Thailand | 2007 | Positive | 6 | 4 | Southeast Asia | Khayhan et al., (2013) |
| CN4998 | CSF | Clinical | Thailand | 2007 | Positive | 6 | 4 | Southeast Asia | Khayhan et al., (2013) |
| CN5010 | Blood | Clinical | Thailand | 2007 | Positive | 6 | 4 | Southeast Asia | Khayhan et al., (2013) |
| UgCl018 | Cerebrospinal fluid | Clinical | Uganda | #N/A | Positive | 4 | 4 | Africa | Wiesner et al. mBio. 2012,3(5):e00196-12 |
| ug2463 | Cerebrospinal fluid | Clinical | Uganda | #N/A | Positive | 6 | 4 | Africa | Litvintseva et al. Genetics. 2006,172(4):2223–2238 |
| BK151 | CSF | Clinical | Vietnam |  | Positive | 4 | 4 | Southeast Asia | This study |
| BK156 | CSF | Clinical | Vietnam |  | Positive | 4 | 4 | Southeast Asia | This study |
| BK163 | CSF | Clinical | Vietnam |  | Positive | 4 | 4 | Southeast Asia | This study |
| BK182 | CSF | Clinical | Vietnam |  | Positive | 4 | 4 | Southeast Asia | This study |
| BK192 | CSF | Clinical | Vietnam |  | Positive | 4 | 4 | Southeast Asia | This study |
| BK193 | CSF | Clinical | Vietnam |  | Positive | 4 | 4 | Southeast Asia | This study |
| BK2 | CSF | Clinical | Vietnam |  | Positive | 4 | 4 | Southeast Asia | This study |
| BK224 | CSF | Clinical | Vietnam |  | Positive | 4 | 4 | Southeast Asia | This study |
| D9 | Avian guano | Environmental | Thailand | 2000 | NA | 141 | 4 | Southeast Asia | Khayhan et al., (2013) |
| BK225 | CSF | Clinical | Vietnam |  | Positive | 4 | 4 | Southeast Asia | This study |
| BK23 | CSF | Clinical | Vietnam |  | Positive | 4 | 4 | Southeast Asia | This study |
| BK30 | CSF | Clinical | Vietnam |  | Positive | 4 | 4 | Southeast Asia | This study |
| BK35 | CSF | Clinical | Vietnam |  | Positive | 4 | 4 | Southeast Asia | This study |
| D42 | Avian guano | Environmental | Thailand | 2000 | NA | 176 | 4 | Southeast Asia | Khayhan et al., (2013) |
| BK48 | CSF | Clinical | Vietnam |  | Positive | 4 | 4 | Southeast Asia | This study |
| BK56 | CSF | Clinical | Vietnam |  | Positive | 4 | 4 | Southeast Asia | This study |
| BK57 | CSF | Clinical | Vietnam |  | Positive | 4 | 4 | Southeast Asia | This study |
| BK59 | CSF | Clinical | Vietnam |  | Positive | 4 | 4 | Southeast Asia | This study |
| BK69 | CSF | Clinical | Vietnam |  | Positive | 4 | 4 | Southeast Asia | This study |
| 25_40 | Unknown | Clinical | India | Unknown | Unknown | 77 | 31 | South Asia | Khayhan et al., (2013) |
| BK73 | CSF | Clinical | Vietnam |  | Positive | 4 | 4 | Southeast Asia | This study |
| 110E | Avian guano | Environmental | Thailand | Unknown | NA | 188 | 4 | Southeast Asia | Khayhan et al., (2013) |
| BK74 | CSF | Clinical | Vietnam |  | Positive | 4 | 4 | Southeast Asia | This study |
| BK80 | CSF | Clinical | Vietnam |  | Positive | 4 | 4 | Southeast Asia | This study |
| 25_311 | CSF | Clinical | India | 2007 | Negative | 189 | 4 | South Asia | Khayhan et al., (2013) |
| 130D | Avian guano | Environmental | Thailand | Unknown | NA | 190 | 4 | Southeast Asia | Khayhan et al., (2013) |
| BK87 | CSF | Clinical | Vietnam |  | Positive | 4 | 4 | Southeast Asia | This study |
| BK88 | CSF | Clinical | Vietnam |  | Positive | 4 | 4 | Southeast Asia | This study |
| BK89 | CSF | Clinical | Vietnam |  | Positive | 4 | 4 | Southeast Asia | This study |
| BK90 | CSF | Clinical | Vietnam |  | Positive | 4 | 4 | Southeast Asia | This study |
| BMD1392 | CSF | Clinical | Vietnam |  | Negative | 4 | 4 | Southeast Asia | This study |
| BMD1415 | CSF | Clinical | Vietnam |  | Negative | 4 | 4 | Southeast Asia | This study |
| BMD1879 | CSF | Clinical | Vietnam |  | Positive | 4 | 4 | Southeast Asia | This study |
| BMD394 | CSF | Clinical | Vietnam |  | Negative | 4 | 4 | Southeast Asia | This study |
| BK115 | CSF | Clinical | Vietnam | Unknown | Positive | 5 | 5 | Southeast Asia | This study |
| 25_78 | Unknown | Clinical | India | 1998 | Unknown | 93 | 31 | South Asia | Khayhan et al., (2013) |
| BK116 | CSF | Clinical | Vietnam | Unknown | Positive | 5 | 5 | Southeast Asia | This study |
| BK117 | CSF | Clinical | Vietnam | Unknown | Positive | 5 | 5 | Southeast Asia | This study |
| BK119 | CSF | Clinical | Vietnam | Unknown | Positive | 5 | 5 | Southeast Asia | This study |
| BK124 | CSF | Clinical | Vietnam | Unknown | Positive | 5 | 5 | Southeast Asia | This study |
| BK139 | CSF | Clinical | Vietnam | Unknown | Positive | 5 | 5 | Southeast Asia | This study |
| BK147 | CSF | Clinical | Vietnam | Unknown | Positive | 5 | 5 | Southeast Asia | This study |
| BK15 | CSF | Clinical | Vietnam | Unknown | Positive | 5 | 5 | Southeast Asia | This study |
| BK160 | CSF | Clinical | Vietnam | Unknown | Positive | 5 | 5 | Southeast Asia | This study |
| BK169 | CSF | Clinical | Vietnam | Unknown | Positive | 5 | 5 | Southeast Asia | This study |
| BK171 | CSF | Clinical | Vietnam | Unknown | Positive | 5 | 5 | Southeast Asia | This study |
| BK175 | CSF | Clinical | Vietnam | Unknown | Positive | 5 | 5 | Southeast Asia | This study |
| BK185 | CSF | Clinical | Vietnam | Unknown | Positive | 5 | 5 | Southeast Asia | This study |
| BK190 | CSF | Clinical | Vietnam | Unknown | Positive | 5 | 5 | Southeast Asia | This study |
| BK20 | CSF | Clinical | Vietnam | Unknown | Positive | 5 | 5 | Southeast Asia | This study |
| BK241 | CSF | Clinical | Vietnam | Unknown | Positive | 5 | 5 | Southeast Asia | This study |
| NRHc5025.ENR.STOR | CSF | Clinical | Botswana | 2012 | Positive | 5 | 5 | Africa | Chen et al., (2016) |
| bt134 | Cerebrospinal fluid | Clinical | Botswana | 2001 | Positive | 25 | 5 | Africa | Litvintseva et al. Genetics. 2006,172(4):2223–2238 |
| CNS128 | CSF | Clinical | Laos | 2003 | Negative | 5 | 5 | Southeast Asia | This study |
| CNS1569 | CSF | Clinical | Laos | 2015 | Negative | 5 | 5 | Southeast Asia | This study |
| UFTM 14.48 | Urine | Clinical | Brazil | 2006 | Positive | 5 | 5 | South America | Ferreira-Paim et al, PlosNTD,2017 |
| UFTM 14.153 | Cerebrospinal fluid | Clinical | Brazil | 2004 | Positive | 5 | 5 | South America | Ferreira-Paim et al, PlosNTD,2017 |
| WH098 | CSF | Clinical | China | 2006 | Positive | 5 | 5 | East Asia | Dou et al., (2014) |
| WH121 | Blood | Clinical | China | 2007 | Positive | 5 | 5 | East Asia | Dou et al., (2014) |
| WH122 | CSF | Clinical | China | Unknown | Positive | 5 | 5 | East Asia | Dou et al., (2014) |
| WH124 | CSF | Clinical | China | Unknown | Positive | 5 | 5 | East Asia | Dou et al., (2014) |
| WH126 | CSF | Clinical | China | Unknown | Positive | 5 | 5 | East Asia | Dou et al., (2014) |
| PU27 | CSF | Clinical | China | Unknown | Positive | 5 | 5 | East Asia | Dou et al., (2014) |
| PU64 | CSF | Clinical | China | Unknown | Positive | 5 | 5 | East Asia | Dou et al., (2014) |
| PU65 | CSF | Clinical | China | Unknown | Positive | 5 | 5 | East Asia | Dou et al., (2014) |
| PU147 | CSF | Clinical | China | Unknown | Positive | 5 | 5 | East Asia | Dou et al., (2014) |
| PU148 | CSF | Clinical | China | Unknown | Positive | 5 | 5 | East Asia | Dou et al., (2014) |
| PU149 | CSF | Clinical | China | Unknown | Positive | 5 | 5 | East Asia | Dou et al., (2014) |
| PU150 | CSF | Clinical | China | Unknown | Positive | 5 | 5 | East Asia | Dou et al., (2014) |
| PU151 | CSF | Clinical | China | Unknown | Positive | 5 | 5 | East Asia | Dou et al., (2014) |
| PU152 | CSF | Clinical | China | Unknown | Positive | 5 | 5 | East Asia | Dou et al., (2014) |
| PU153 | CSF | Clinical | China | Unknown | Positive | 5 | 5 | East Asia | Dou et al., (2014) |
| PU154 | CSF | Clinical | China | Unknown | Positive | 5 | 5 | East Asia | Dou et al., (2014) |
| PU155 | CSF | Clinical | China | Unknown | Positive | 5 | 5 | East Asia | Dou et al., (2014) |
| PU156 | CSF | Clinical | China | Unknown | Positive | 5 | 5 | East Asia | Dou et al., (2014) |
| PU158 | CSF | Clinical | China | Unknown | Positive | 5 | 5 | East Asia | Dou et al., (2014) |
| PU159 | CSF | Clinical | China | Unknown | Positive | 5 | 5 | East Asia | Dou et al., (2014) |
| PU160 | CSF | Clinical | China | Unknown | Positive | 5 | 5 | East Asia | Dou et al., (2014) |
| PU163 | CSF | Clinical | China | Unknown | Positive | 5 | 5 | East Asia | Dou et al., (2014) |
| PU164 | CSF | Clinical | China | Unknown | Positive | 5 | 5 | East Asia | Dou et al., (2014) |
| PU167 | CSF | Clinical | China | Unknown | Positive | 5 | 5 | East Asia | Dou et al., (2014) |
| WH003 | Sputum | Clinical | China | 1998 | Negative | 5 | 5 | East Asia | Khayhan et al., (2013) |
| WH004 | CSF | Clinical | China | 2001 | Negative | 5 | 5 | East Asia | Khayhan et al., (2013) |
| WH005 | CSF | Clinical | China | Unknown | Negative | 5 | 5 | East Asia | Khayhan et al., (2013) |
| WH006 | CSF | Clinical | China | Unknown | Negative | 5 | 5 | East Asia | Khayhan et al., (2013) |
| WH007 | CSF | Clinical | China | Unknown | Negative | 5 | 5 | East Asia | Khayhan et al., (2013) |
| WH008 | CSF | Clinical | China | Unknown | Negative | 5 | 5 | East Asia | Khayhan et al., (2013) |
| WH011 | CSF | Clinical | China | Unknown | Negative | 5 | 5 | East Asia | Khayhan et al., (2013) |
| WH012 | CSF | Clinical | China | 1993 | Negative | 5 | 5 | East Asia | Khayhan et al., (2013) |
| WH013 | CSF | Clinical | China | 1998 | Negative | 5 | 5 | East Asia | Khayhan et al., (2013) |
| WH014 | CSF | Clinical | China | Unknown | Negative | 5 | 5 | East Asia | Khayhan et al., (2013) |
| WH015 | Sputum | Clinical | China | 2003 | Negative | 5 | 5 | East Asia | Khayhan et al., (2013) |
| WH016 | CSF | Clinical | China | Unknown | Negative | 5 | 5 | East Asia | Khayhan et al., (2013) |
| WH017 | CSF | Clinical | China | Unknown | Negative | 5 | 5 | East Asia | Khayhan et al., (2013) |
| WH019 | CSF | Clinical | China | Unknown | Negative | 5 | 5 | East Asia | Khayhan et al., (2013) |
| WH020 | Blood | Clinical | China | 2003 | Negative | 5 | 5 | East Asia | Khayhan et al., (2013) |
| WH022 | CSF | Clinical | China | Unknown | Negative | 5 | 5 | East Asia | Khayhan et al., (2013) |
| WH023 | CSF | Clinical | China | Unknown | Negative | 5 | 5 | East Asia | Khayhan et al., (2013) |
| WH025 | CSF | Clinical | China | 2001 | Negative | 5 | 5 | East Asia | Khayhan et al., (2013) |
| WH026 | CSF | Clinical | China | Unknown | Negative | 5 | 5 | East Asia | Khayhan et al., (2013) |
| WH027 | CSF | Clinical | China | 2005 | Negative | 5 | 5 | East Asia | Khayhan et al., (2013) |
| WH028 | CSF | Clinical | China | Unknown | Negative | 5 | 5 | East Asia | Khayhan et al., (2013) |
| WH030 | CSF | Clinical | China | Unknown | Negative | 5 | 5 | East Asia | Khayhan et al., (2013) |
| WH031 | Lung | Clinical | China | 2000 | Negative | 5 | 5 | East Asia | Khayhan et al., (2013) |
| WH033 | Skin | Clinical | China | 2000 | Negative | 5 | 5 | East Asia | Khayhan et al., (2013) |
| WH034 | CSF | Clinical | China | Unknown | Negative | 5 | 5 | East Asia | Khayhan et al., (2013) |
| WH035 | CSF | Clinical | China | Unknown | Negative | 5 | 5 | East Asia | Khayhan et al., (2013) |
| WH036 | CSF | Clinical | China | 2003 | Negative | 5 | 5 | East Asia | Khayhan et al., (2013) |
| WH040 | CSF | Clinical | China | 2003 | Negative | 5 | 5 | East Asia | Khayhan et al., (2013) |
| WH041 | CSF | Clinical | China | Unknown | Negative | 5 | 5 | East Asia | Khayhan et al., (2013) |
| WH042 | CSF | Clinical | China | 2004 | Negative | 5 | 5 | East Asia | Khayhan et al., (2013) |
| WH044 | CSF | Clinical | China | Unknown | Negative | 5 | 5 | East Asia | Khayhan et al., (2013) |
| WH047 | CSF | Clinical | China | Unknown | Negative | 5 | 5 | East Asia | Khayhan et al., (2013) |
| WH050 | CSF | Clinical | China | Unknown | Negative | 5 | 5 | East Asia | Khayhan et al., (2013) |
| WH051 | CSF | Clinical | China | Unknown | Negative | 5 | 5 | East Asia | Khayhan et al., (2013) |
| WH054 | CSF | Clinical | China | Unknown | Negative | 5 | 5 | East Asia | Khayhan et al., (2013) |
| WH055 | CSF | Clinical | China | Unknown | Negative | 5 | 5 | East Asia | Khayhan et al., (2013) |
| WH057 | CSF | Clinical | China | 2005 | Negative | 5 | 5 | East Asia | Khayhan et al., (2013) |
| WH058 | Skin | Clinical | China | 2004 | Negative | 5 | 5 | East Asia | Khayhan et al., (2013) |
| WH061 | CSF | Clinical | China | Unknown | Negative | 5 | 5 | East Asia | Khayhan et al., (2013) |
| WH062 | Skin | Clinical | China | 2001 | Negative | 5 | 5 | East Asia | Khayhan et al., (2013) |
| WH063 | CSF | Clinical | China | 2008 | Negative | 5 | 5 | East Asia | Khayhan et al., (2013) |
| WH066 | CSF | Clinical | China | Unknown | Negative | 5 | 5 | East Asia | Khayhan et al., (2013) |
| WH067 | CSF | Clinical | China | Unknown | Negative | 5 | 5 | East Asia | Khayhan et al., (2013) |
| WH068 | CSF | Clinical | China | 1997 | Negative | 5 | 5 | East Asia | Khayhan et al., (2013) |
| WH072 | CSF | Clinical | China | Unknown | Negative | 5 | 5 | East Asia | Khayhan et al., (2013) |
| WH075 | CSF | Clinical | China | Unknown | Negative | 5 | 5 | East Asia | Khayhan et al., (2013) |
| WH076 | CSF | Clinical | China | Unknown | Negative | 5 | 5 | East Asia | Khayhan et al., (2013) |
| WH077 | CSF | Clinical | China | 2001 | Negative | 5 | 5 | East Asia | Khayhan et al., (2013) |
| WH078 | CSF | Clinical | China | Unknown | Negative | 5 | 5 | East Asia | Khayhan et al., (2013) |
| WH079 | CSF | Clinical | China | Unknown | Negative | 5 | 5 | East Asia | Khayhan et al., (2013) |
| WH080 | CSF | Clinical | China | Unknown | Negative | 5 | 5 | East Asia | Khayhan et al., (2013) |
| WH082 | CSF | Clinical | China | Unknown | Negative | 5 | 5 | East Asia | Khayhan et al., (2013) |
| WH083 | Blood | Clinical | China | 2001 | Negative | 5 | 5 | East Asia | Khayhan et al., (2013) |
| WH091 | CSF | Clinical | China | Unknown | Negative | 5 | 5 | East Asia | Khayhan et al., (2013) |
| WH093 | CSF | Clinical | China | 1999 | Negative | 5 | 5 | East Asia | Khayhan et al., (2013) |
| WH094 | CSF | Clinical | China | Unknown | Negative | 5 | 5 | East Asia | Khayhan et al., (2013) |
| WH096 | CSF | Clinical | China | Unknown | Negative | 5 | 5 | East Asia | Khayhan et al., (2013) |
| WH101 | CSF | Clinical | China | 2007 | Negative | 5 | 5 | East Asia | Khayhan et al., (2013) |
| WH102 | CSF | Clinical | China | 2002 | Negative | 5 | 5 | East Asia | Khayhan et al., (2013) |
| WH104 | CSF | Clinical | China | 1996 | Negative | 5 | 5 | East Asia | Khayhan et al., (2013) |
| WH105 | CSF | Clinical | China | 2004 | Negative | 5 | 5 | East Asia | Khayhan et al., (2013) |
| WH106 | CSF | Clinical | China | Unknown | Negative | 5 | 5 | East Asia | Khayhan et al., (2013) |
| WH108 | CSF | Clinical | China | 2005 | Negative | 5 | 5 | East Asia | Khayhan et al., (2013) |
| WH114 | CSF | Clinical | China | 2000 | Negative | 5 | 5 | East Asia | Khayhan et al., (2013) |
| WH115 | CSF | Clinical | China | Unknown | Negative | 5 | 5 | East Asia | Khayhan et al., (2013) |
| WH117 | CSF | Clinical | China | Unknown | Negative | 5 | 5 | East Asia | Khayhan et al., (2013) |
| WH118 | CSF | Clinical | China | 1995 | Negative | 5 | 5 | East Asia | Khayhan et al., (2013) |
| WH119 | CSF | Clinical | China | 2001 | Negative | 5 | 5 | East Asia | Khayhan et al., (2013) |
| WH120 | CSF | Clinical | China | 2003 | Negative | 5 | 5 | East Asia | Khayhan et al., (2013) |
| PU1 | CSF | Clinical | China | Unknown | Negative | 5 | 5 | East Asia | Dou et al., (2014) |
| PU16 | CSF | Clinical | China | Unknown | Negative | 5 | 5 | East Asia | Dou et al., (2014) |
| PU18 | CSF | Clinical | China | Unknown | Negative | 5 | 5 | East Asia | Dou et al., (2014) |
| PU30 | CSF | Clinical | China | Unknown | Negative | 5 | 5 | East Asia | Dou et al., (2014) |
| PU33 | CSF | Clinical | China | Unknown | Negative | 5 | 5 | East Asia | Dou et al., (2014) |
| PU38 | CSF | Clinical | China | Unknown | Negative | 5 | 5 | East Asia | Dou et al., (2014) |
| PU44 | CSF | Clinical | China | Unknown | Negative | 5 | 5 | East Asia | Dou et al., (2014) |
| PU47 | CSF | Clinical | China | Unknown | Negative | 5 | 5 | East Asia | Dou et al., (2014) |
| PU49 | CSF | Clinical | China | Unknown | Negative | 5 | 5 | East Asia | Dou et al., (2014) |
| PU67 | CSF | Clinical | China | Unknown | Negative | 5 | 5 | East Asia | Dou et al., (2014) |
| PU145 | CSF | Clinical | China | Unknown | Negative | 5 | 5 | East Asia | Dou et al., (2014) |
| 4_187 | Clinical | Clinical | Thailand | Unknown | Unknown | 6 | 4 | Southeast Asia | Khayhan et al., (2013) |
| 4_202 | Clinical | Clinical | Thailand | Unknown | Unknown | 6 | 4 | Southeast Asia | Khayhan et al., (2013) |
| 4_231 | Clinical | Clinical | Thailand | Unknown | Unknown | 4 | 4 | Southeast Asia | Khayhan et al., (2013) |
| 4_253 | Clinical | Clinical | Thailand | Unknown | Unknown | 4 | 4 | Southeast Asia | Khayhan et al., (2013) |
| 4_315 | Clinical | Clinical | Thailand | Unknown | Unknown | 6 | 4 | Southeast Asia | Khayhan et al., (2013) |
| 4_319 | Clinical | Clinical | Thailand | Unknown | Unknown | 4 | 4 | Southeast Asia | Khayhan et al., (2013) |
| 4_381 | Clinical | Clinical | Thailand | Unknown | Unknown | 4 | 4 | Southeast Asia | Khayhan et al., (2013) |
| 4_83 | Clinical | Clinical | Thailand | Unknown | Unknown | 6 | 4 | Southeast Asia | Khayhan et al., (2013) |
| 4_9 | Clinical | Clinical | Thailand | Unknown | Unknown | 82 | 4 | Southeast Asia | Khayhan et al., (2013) |
| AD1-12 | Blood | Clinical | France | Unknown | Unknown | 324 | 63 | Europe | Desnos-Ollivier et al. mBio. 2010,1(1):e00091-10 |
| 10-0603 | Unknown | Clinical | Germany | 2010 | Organ transplant | 63 | 63 | Europe | Sanchini et al. Med Microbiol Immunol. 2014,203(5):333-340 |
| 08-0251 | Unknown | Clinical | Germany | 2008 | Liver disorder | 67 | 63 | Europe | Sanchini et al. Med Microbiol Immunol. 2014,203(5):333-340 |
| 9104 | Skin | Clinical | Japan | 1988 | Negative | 5 | 5 | East Asia | Khayhan et al., (2013) |
| 9106 | CSF | Clinical | Japan | 1988 | Negative | 5 | 5 | East Asia | Khayhan et al., (2013) |
| 9107 | Unknown | Clinical | Japan | 1988 | Negative | 5 | 5 | East Asia | Khayhan et al., (2013) |
| 9108 | Skin | Clinical | Japan | 1987 | Negative | 5 | 5 | East Asia | Khayhan et al., (2013) |
| 9111 | CSF | Clinical | Japan | 1986 | Negative | 5 | 5 | East Asia | Khayhan et al., (2013) |
| 9114 | Unknown | Clinical | Japan | Unknown | Negative | 5 | 5 | East Asia | Khayhan et al., (2013) |
| 9165 | CSF | Clinical | Japan | 1983 | Negative | 5 | 5 | East Asia | Khayhan et al., (2013) |
| 9166 | Skin | Clinical | Japan | 1983 | Negative | 5 | 5 | East Asia | Khayhan et al., (2013) |
| 9167 | CSF | Clinical | Japan | 1983 | Negative | 5 | 5 | East Asia | Khayhan et al., (2013) |
| 9170 | Skin | Clinical | Japan | 1984 | Negative | 5 | 5 | East Asia | Khayhan et al., (2013) |
| 09-0829 | Unknown | Clinical | Germany | 2009 | Organ transplant | 289 | 63 | Europe | Sanchini et al. Med Microbiol Immunol. 2014,203(5):333-340 |
| 9173 | CSF | Clinical | Japan | 1985 | Negative | 5 | 5 | East Asia | Khayhan et al., (2013) |
| 9174 | CSF | Clinical | Japan | 1985 | Negative | 5 | 5 | East Asia | Khayhan et al., (2013) |
| 9179 | Unknown | Clinical | Japan | 1987 | Negative | 5 | 5 | East Asia | Khayhan et al., (2013) |
| AD1-76 | CSF | Clinical | France | Unknown | Unknown | 5 | 5 | Europe | Desnos-Ollivier et al. mBio. 2010,1(1):e00091-10 |
| AD4-27A | CSF | Clinical | France | Unknown | Unknown | 75 | 31 | Europe | Desnos-Ollivier et al. mBio. 2010,1(1):e00091-10 |
| AD4-5 | CSF | Clinical | France | Unknown | Unknown | 23 | 63 | Europe | Desnos-Ollivier et al. mBio. 2010,1(1):e00091-10 |
| arg1366 | Unknown | Unknown | Argentina | Unknown | Unknown | 2 | 63 | South America | Litvintseva et al. Genetics. 2006,172(4):2223–2238 |
| br2362 | Unknown | Unknown | Brazil | Unknown | Unknown | 32 | 31 | South America | Litvintseva et al. Genetics. 2006,172(4):2223–2238 |
| 9211 | Avian guano | Environmental | Japan | 2005 | NA | 5 | 5 | East Asia | Khayhan et al., (2013) |
| 9212 | Avian guano | Environmental | Japan | 2005 | NA | 5 | 5 | East Asia | Khayhan et al., (2013) |
| C5 | CSF | Clinical | China | Unknown | Unknown | 31 | 31 | East Asia | Wu et al. Mycoses. 2015,58(5):280-287 |
| CN48 | Clinical | Clinical | Thailand | Unknown | Unknown | 5 | 5 | Southeast Asia | Khayhan et al., (2013) |
| 9237 | Skin | Clinical | Japan | 1994 | Negative | 5 | 5 | East Asia | Khayhan et al., (2013) |
| 9238 | CSF | Clinical | Japan | 1990 | Negative | 5 | 5 | East Asia | Khayhan et al., (2013) |
| 9239 | Skin | Clinical | Japan | 1990 | Negative | 5 | 5 | East Asia | Khayhan et al., (2013) |
| 9251 | Skin | Clinical | Japan | 2001 | Negative | 5 | 5 | East Asia | Khayhan et al., (2013) |
| 9254 | Avian guano | Environmental | Japan | 2005 | NA | 5 | 5 | East Asia | Khayhan et al., (2013) |
| 9255 | Avian guano | Environmental | Japan | 2005 | NA | 5 | 5 | East Asia | Khayhan et al., (2013) |
| 9258 | Avian guano | Environmental | Japan | 2005 | NA | 5 | 5 | East Asia | Khayhan et al., (2013) |
| 9259 | Avian guano | Environmental | Japan | 2005 | NA | 5 | 5 | East Asia | Khayhan et al., (2013) |
| 9260 | Avian guano | Environmental | Japan | 2005 | NA | 5 | 5 | East Asia | Khayhan et al., (2013) |
| 9261 | Avian guano | Environmental | Japan | 2005 | NA | 5 | 5 | East Asia | Khayhan et al., (2013) |
| 9263 | Skin | Clinical | Japan | 2003 | Negative | 5 | 5 | East Asia | Khayhan et al., (2013) |
| 9264 | Skin | Clinical | Japan | 2002 | Negative | 5 | 5 | East Asia | Khayhan et al., (2013) |
| 9265 | Skin | Clinical | Japan | 2001 | Negative | 5 | 5 | East Asia | Khayhan et al., (2013) |
| 1185_04 | Endotracheal secretion | Clinical | Kuwait | 2004 | Negative | 5 | 5 | Middle East | Khayhan et al., (2013) |
| 201_95 | Lumbar swellg aspirate | Clinical | Kuwait | 1995 | Negative | 5 | 5 | Middle East | Khayhan et al., (2013) |
| WH123 | CSF | Clinical | China | 2007 | Positive | 194 | 5 | East Asia | Khayhan et al., (2013) |
| PU161 | CSF | Clinical | China | Unknown | Positive | 296 | 5 | East Asia | Khayhan et al., (2013) |
| 1609290340 | CSF | Clinical | Qatar |  | Negative | 5 | 5 | Middle East | Khayhan et al., (2013) |
| 09-0007 | Unknown | Clinical | Germany | 2009 | Positive | 288 | 5 | Europe | Sanchini et al. Med Microbiol Immunol. 2014,203(5):333-340 |
| 40 | Avian guano | Environmental | Thailand | Unknown | NA | 5 | 5 | Southeast Asia | Khayhan et al., (2013) |
| CNS245 | CSF | Clinical | Laos | 2005 | Unknown | 4 | 4 | Southeast Asia | This study |
| CNS430 | CSF | Clinical | Laos | 2006 | Unknown | 6 | 4 | Southeast Asia | This study |
| 189E | Avian guano | Environmental | Thailand | Unknown | NA | 5 | 5 | Southeast Asia | Khayhan et al., (2013) |
| CNS57 | CSF | Clinical | Laos | 2003 | Unknown | 4 | 4 | Southeast Asia | This study |
| D16 | Avian guano | Environmental | Thailand | 2000 | NA | 5 | 5 | Southeast Asia | Khayhan et al., (2013) |
| D4 | Avian guano | Environmental | Thailand | 2000 | NA | 5 | 5 | Southeast Asia | Khayhan et al., (2013) |
| D6 | Avian guano | Environmental | Thailand | 2000 | NA | 5 | 5 | Southeast Asia | Khayhan et al., (2013) |
| PG37 | Avian guano | Environmental | Thailand | 2000 | NA | 5 | 5 | Southeast Asia | Khayhan et al., (2013) |
| BK25 | CSF | Clinical | Vietnam | Unknown | Positive | 5 | 5 | Southeast Asia | This study |
| BK26 | CSF | Clinical | Vietnam | Unknown | Positive | 5 | 5 | Southeast Asia | This study |
| BK28 | CSF | Clinical | Vietnam | Unknown | Positive | 5 | 5 | Southeast Asia | This study |
| BK34 | CSF | Clinical | Vietnam | Unknown | Positive | 5 | 5 | Southeast Asia | This study |
| BK38 | CSF | Clinical | Vietnam | Unknown | Positive | 5 | 5 | Southeast Asia | This study |
| BK4 | CSF | Clinical | Vietnam | Unknown | Positive | 5 | 5 | Southeast Asia | This study |
| BK41 | CSF | Clinical | Vietnam | Unknown | Positive | 5 | 5 | Southeast Asia | This study |
| BK42 | CSF | Clinical | Vietnam | Unknown | Positive | 5 | 5 | Southeast Asia | This study |
| BK44 | CSF | Clinical | Vietnam | Unknown | Positive | 5 | 5 | Southeast Asia | This study |
| BK45 | CSF | Clinical | Vietnam | Unknown | Positive | 5 | 5 | Southeast Asia | This study |
| BK49 | CSF | Clinical | Vietnam | Unknown | Positive | 5 | 5 | Southeast Asia | This study |
| BK50 | CSF | Clinical | Vietnam | Unknown | Positive | 5 | 5 | Southeast Asia | This study |
| BK54 | CSF | Clinical | Vietnam | Unknown | Positive | 5 | 5 | Southeast Asia | This study |
| BK58 | CSF | Clinical | Vietnam | Unknown | Positive | 5 | 5 | Southeast Asia | This study |
| BK62 | CSF | Clinical | Vietnam | Unknown | Positive | 5 | 5 | Southeast Asia | This study |
| BK63 | CSF | Clinical | Vietnam | Unknown | Positive | 5 | 5 | Southeast Asia | This study |
| BK76 | CSF | Clinical | Vietnam | Unknown | Positive | 5 | 5 | Southeast Asia | This study |
| BK78 | CSF | Clinical | Vietnam | Unknown | Positive | 5 | 5 | Southeast Asia | This study |
| BK94 | CSF | Clinical | Vietnam | Unknown | Positive | 5 | 5 | Southeast Asia | This study |
| BMD101 | CSF | Clinical | Vietnam | Unknown | Negative | 5 | 5 | Southeast Asia | This study |
| BMD1198 | CSF | Clinical | Vietnam | Unknown | Negative | 5 | 5 | Southeast Asia | This study |
| BMD1228 | CSF | Clinical | Vietnam | Unknown | Negative | 5 | 5 | Southeast Asia | This study |
| BMD1232 | CSF | Clinical | Vietnam | Unknown | Negative | 5 | 5 | Southeast Asia | This study |
| BMD1291 | CSF | Clinical | Vietnam | Unknown | Negative | 5 | 5 | Southeast Asia | This study |
| BMD1338 | CSF | Clinical | Vietnam | Unknown | Negative | 5 | 5 | Southeast Asia | This study |
| BMD1353 | CSF | Clinical | Vietnam | Unknown | Negative | 5 | 5 | Southeast Asia | This study |
| BMD1452 | CSF | Clinical | Vietnam | Unknown | Negative | 5 | 5 | Southeast Asia | This study |
| BMD1465 | CSF | Clinical | Vietnam | Unknown | Negative | 5 | 5 | Southeast Asia | This study |
| BMD1534 | CSF | Clinical | Vietnam | Unknown | Negative | 5 | 5 | Southeast Asia | This study |
| BMD1592 | CSF | Clinical | Vietnam | Unknown | Negative | 5 | 5 | Southeast Asia | This study |
| BMD1646 | CSF | Clinical | Vietnam | Unknown | Negative | 5 | 5 | Southeast Asia | This study |
| CNS595 | CSF | Clinical | Laos | 2008 | Unknown | 4 | 4 | Southeast Asia | This study |
| CNS811 | CSF | Clinical | Laos | 2009 | Unknown | 93 | 31 | Southeast Asia | This study |
| A5 35-17 | Avian guano | Enviromental | USA | Unknown | NA | 5 | 5 | North America | Litvintseva et al. Genetics. 2006,172(4):2223–2238; Litvintseva et al. PlosOne. 2011, 6(5):e19688 |
| blg7 | Avian guano | Enviromental | Belgium | Unknown | NA | 5 | 5 | Europe | Litvintseva et al. Genetics. 2006,172(4):2223–2238 |
| CNS936 | CSF | Clinical | Laos | 2010 | Unknown | 5 | 5 | Southeast Asia | This study |
| UFTM 14.32 | Enviromental | Enviromental | Brazil | Unknown | NA | 5 | 5 | South America | Ferreira-Paim et al, PlosNTD,2017 |
| IUM 01-2006 | Blood | Clinical | Italy | #N/A | Positive | 84 | 5 | Europe | Cogliati et al. Medical Mycology. 2013,51:499–506 |
| 04CN30_002 / UI24734/ CNS1292_3 | CSF | Clinical | Laos | Unknown | Positive | 5 | 5 | Southeast Asia | This study |
| 04CN30_016/ UI14893/ CNS605_2 | CSF | Clinical | Laos | Unknown | Positive | 5 | 5 | Southeast Asia | This study |
| 04CN30_019/ UI29069/ CNS1415_2 | CSF | Clinical | Laos | Unknown | Positive | 5 | 5 | Southeast Asia | This study |
| CNS121 | CSF | Clinical | Laos | 2003 | Positive | 5 | 5 | Southeast Asia | This study |
| CNS1396_2 | CSF | Clinical | Laos | 2014 | Positive | 5 | 5 | Southeast Asia | This study |
| CNS517 | CSF | Clinical | Laos | 2007 | Positive | 5 | 5 | Southeast Asia | This study |
| 1607001262 | CSF | Clinical | Qatar |  | Positive | 5 | 5 | Middle East | Khayhan et al., (2013) |
| 107 | CSF | Clinical | South Africa |  | Positive | 5 | 5 | Africa | Beale MA et al, Plos NTD,2015 |
| 101 | CSF | Clinical | South Africa |  | Positive | 5 | 5 | Africa | Beale MA et al, Plos NTD,2015 |
| 118 | CSF | Clinical | South Africa |  | Positive | 5 | 5 | Africa | Beale MA et al, Plos NTD,2015 |
| 133 | CSF | Clinical | South Africa |  | Positive | 5 | 5 | Africa | Beale MA et al, Plos NTD,2015 |
| 145 | CSF | Clinical | South Africa |  | Positive | 5 | 5 | Africa | Beale MA et al, Plos NTD,2015 |
| 229 | CSF | Clinical | South Africa |  | Positive | 5 | 5 | Africa | Beale MA et al, Plos NTD,2015 |
| 337 | CSF | Clinical | South Africa |  | Positive | 5 | 5 | Africa | Beale MA et al, Plos NTD,2015 |
| 204 | CSF | Clinical | South Africa |  | Positive | 5 | 5 | Africa | Beale MA et al, Plos NTD,2015 |
| 326 | CSF | Clinical | South Africa |  | Positive | 5 | 5 | Africa | Beale MA et al, Plos NTD,2015 |
| 339 | CSF | Clinical | South Africa |  | Positive | 5 | 5 | Africa | Beale MA et al, Plos NTD,2015 |
| 341 | CSF | Clinical | South Africa |  | Positive | 5 | 5 | Africa | Beale MA et al, Plos NTD,2015 |
| 401 | CSF | Clinical | South Africa |  | Positive | 5 | 5 | Africa | Beale MA et al, Plos NTD,2015 |
| 428 | CSF | Clinical | South Africa |  | Positive | 5 | 5 | Africa | Beale MA et al, Plos NTD,2015 |
| 437 | CSF | Clinical | South Africa |  | Positive | 5 | 5 | Africa | Beale MA et al, Plos NTD,2015 |
| 468 | CSF | Clinical | South Africa |  | Positive | 5 | 5 | Africa | Beale MA et al, Plos NTD,2015 |
| 478 | CSF | Clinical | South Africa |  | Positive | 5 | 5 | Africa | Beale MA et al, Plos NTD,2015 |
| 314 | CSF | Clinical | South Africa |  | Positive | 5 | 5 | Africa | Beale MA et al, Plos NTD,2015 |
| 343 | CSF | Clinical | South Africa |  | Positive | 5 | 5 | Africa | Beale MA et al, Plos NTD,2015 |
| 425 | CSF | Clinical | South Africa |  | Positive | 5 | 5 | Africa | Beale MA et al, Plos NTD,2015 |
| 457 | CSF | Clinical | South Africa |  | Positive | 5 | 5 | Africa | Beale MA et al, Plos NTD,2015 |
| 470 | CSF | Clinical | South Africa |  | Positive | 5 | 5 | Africa | Beale MA et al, Plos NTD,2015 |
| 420 | CSF | Clinical | South Africa |  | Positive | 5 | 5 | Africa | Beale MA et al, Plos NTD,2015 |
| 424 | CSF | Clinical | South Africa |  | Positive | 5 | 5 | Africa | Beale MA et al, Plos NTD,2015 |
| 443 | CSF | Clinical | South Africa |  | Positive | 5 | 5 | Africa | Beale MA et al, Plos NTD,2015 |
| 447 | CSF | Clinical | South Africa |  | Positive | 5 | 5 | Africa | Beale MA et al, Plos NTD,2015 |
| 465 | CSF | Clinical | South Africa |  | Positive | 5 | 5 | Africa | Beale MA et al, Plos NTD,2015 |
| 475 | CSF | Clinical | South Africa |  | Positive | 5 | 5 | Africa | Beale MA et al, Plos NTD,2015 |
| 218 | CSF | Clinical | South Africa |  | Positive | 202 | 5 | Africa | Beale MA et al, Plos NTD,2015 |
| 219 | CSF | Clinical | South Africa |  | Positive | 202 | 5 | Africa | Beale MA et al, Plos NTD,2015 |
| 263 | CSF | Clinical | South Africa |  | Positive | 202 | 5 | Africa | Beale MA et al, Plos NTD,2015 |
| HK_01 | Clinical | Clinical | China | Unknown | Unknown | 6 | 4 | East Asia | Khayhan et al., (2013) |
| CM30 | CSF | Clinical | Thailand | 2002 | Positive | 5 | 5 | Southeast Asia | Khayhan et al., (2013) |
| CN49004 | CSF | Clinical | Thailand |  | Positive | 5 | 5 | Southeast Asia | Khayhan et al., (2013) |
| CN49006 | CSF | Clinical | Thailand |  | Positive | 5 | 5 | Southeast Asia | Khayhan et al., (2013) |
| CN4906 | CSF | Clinical | Thailand |  | Positive | 5 | 5 | Southeast Asia | Khayhan et al., (2013) |
| CN4916 | CSF | Clinical | Thailand |  | Positive | 5 | 5 | Southeast Asia | Khayhan et al., (2013) |
| CN4920 | CSF | Clinical | Thailand |  | Positive | 5 | 5 | Southeast Asia | Khayhan et al., (2013) |
| CN4921 | CSF | Clinical | Thailand | 2006 | Positive | 5 | 5 | Southeast Asia | Khayhan et al., (2013) |
| CN4924 | CSF | Clinical | Thailand |  | Positive | 5 | 5 | Southeast Asia | Khayhan et al., (2013) |
| CN4946 | CSF | Clinical | Thailand |  | Positive | 5 | 5 | Southeast Asia | Khayhan et al., (2013) |
| CN4948 | CSF | Clinical | Thailand |  | Positive | 5 | 5 | Southeast Asia | Khayhan et al., (2013) |
| CN4960 | CSF | Clinical | Thailand |  | Positive | 5 | 5 | Southeast Asia | Khayhan et al., (2013) |
| CN4967 | CSF | Clinical | Thailand |  | Positive | 5 | 5 | Southeast Asia | Khayhan et al., (2013) |
| CN4977 | CSF | Clinical | Thailand |  | Positive | 5 | 5 | Southeast Asia | Khayhan et al., (2013) |
| CN4980 | CSF | Clinical | Thailand |  | Positive | 5 | 5 | Southeast Asia | Khayhan et al., (2013) |
| CN4983 | CSF | Clinical | Thailand |  | Positive | 5 | 5 | Southeast Asia | Khayhan et al., (2013) |
| CN4993 | CSF | Clinical | Thailand |  | Positive | 5 | 5 | Southeast Asia | Khayhan et al., (2013) |
| CN5008 | CSF | Clinical | Thailand |  | Positive | 5 | 5 | Southeast Asia | Khayhan et al., (2013) |
| CN5012 | CSF | Clinical | Thailand |  | Positive | 5 | 5 | Southeast Asia | Khayhan et al., (2013) |
| CN5015 | CSF | Clinical | Thailand |  | Positive | 5 | 5 | Southeast Asia | Khayhan et al., (2013) |
| CN5018 | Blood | Clinical | Thailand | 2008 | Positive | 5 | 5 | Southeast Asia | Khayhan et al., (2013) |
| UgCl021 | Cerebrospinal fluid | Clinical | Uganda | #N/A | Positive | 5 | 5 | Africa | Wiesner et al. mBio. 2012,3(5):e00196-12 |
| HK_02 | Clinical | Clinical | China | Unknown | Unknown | 4 | 4 | East Asia | Khayhan et al., (2013) |
| WH095 | Ure | Clinical | China | 2001 | Negative | 186 | 5 | East Asia | Khayhan et al., (2013) |
| D44 | Avian guano | Environmental | Thailand | 2000 | NA | 193 | 5 | Southeast Asia | Khayhan et al., (2013) |
| WH071 | CSF | Clinical | China | 1998 | Negative | 194 | 5 | East Asia | Khayhan et al., (2013) |
| BMD1713 | CSF | Clinical | Vietnam | Unknown | Negative | 5 | 5 | Southeast Asia | This study |
| BMD1716 | CSF | Clinical | Vietnam | Unknown | Negative | 5 | 5 | Southeast Asia | This study |
| BMD1828 | CSF | Clinical | Vietnam | Unknown | Negative | 5 | 5 | Southeast Asia | This study |
| BMD367 | CSF | Clinical | Vietnam | Unknown | Negative | 5 | 5 | Southeast Asia | This study |
| BMD368 | CSF | Clinical | Vietnam | Unknown | Negative | 5 | 5 | Southeast Asia | This study |
| BMD494 | CSF | Clinical | Vietnam | Unknown | Negative | 5 | 5 | Southeast Asia | This study |
| BMD534 | CSF | Clinical | Vietnam | Unknown | Negative | 5 | 5 | Southeast Asia | This study |
| BMD673 | CSF | Clinical | Vietnam | Unknown | Negative | 5 | 5 | Southeast Asia | This study |
| BMD700 | CSF | Clinical | Vietnam | Unknown | Negative | 5 | 5 | Southeast Asia | This study |
| BMD732 | CSF | Clinical | Vietnam | Unknown | Negative | 5 | 5 | Southeast Asia | This study |
| BMD761 | CSF | Clinical | Vietnam | Unknown | Negative | 5 | 5 | Southeast Asia | This study |
| BMD852 | CSF | Clinical | Vietnam | Unknown | Negative | 5 | 5 | Southeast Asia | This study |
| BMD854 | CSF | Clinical | Vietnam | Unknown | Negative | 5 | 5 | Southeast Asia | This study |
| BMD865 | CSF | Clinical | Vietnam | Unknown | Negative | 5 | 5 | Southeast Asia | This study |
| BMD894 | CSF | Clinical | Vietnam | Unknown | Negative | 5 | 5 | Southeast Asia | This study |
| BMD899 | CSF | Clinical | Vietnam | Unknown | Negative | 5 | 5 | Southeast Asia | This study |
| BMD903 | CSF | Clinical | Vietnam | Unknown | Negative | 5 | 5 | Southeast Asia | This study |
| BMD910 | CSF | Clinical | Vietnam | Unknown | Negative | 5 | 5 | Southeast Asia | This study |
| BMD973 | CSF | Clinical | Vietnam | Unknown | Negative | 5 | 5 | Southeast Asia | This study |
| BK18 | CSF | Clinical | Vietnam | Unknown | Positive | 6 | 4 | Southeast Asia | This study |
| BK188 | CSF | Clinical | Vietnam | Unknown | Positive | 6 | 4 | Southeast Asia | This study |
| BK189 | CSF | Clinical | Vietnam | Unknown | Positive | 6 | 4 | Southeast Asia | This study |
| BK205 | CSF | Clinical | Vietnam | Unknown | Positive | 6 | 4 | Southeast Asia | This study |
| BK218 | CSF | Clinical | Vietnam | Unknown | Positive | 6 | 4 | Southeast Asia | This study |
| BK219 | CSF | Clinical | Vietnam | Unknown | Positive | 6 | 4 | Southeast Asia | This study |
| BK234 | CSF | Clinical | Vietnam | Unknown | Positive | 6 | 4 | Southeast Asia | This study |
| BK52 | CSF | Clinical | Vietnam | Unknown | Positive | 6 | 4 | Southeast Asia | This study |
| BK71 | CSF | Clinical | Vietnam | Unknown | Positive | 6 | 4 | Southeast Asia | This study |
| BK75 | CSF | Clinical | Vietnam | Unknown | Positive | 6 | 4 | Southeast Asia | This study |
| BK96 | CSF | Clinical | Vietnam | Unknown | Positive | 6 | 4 | Southeast Asia | This study |
| BMD745 | CSF | Clinical | Vietnam | Unknown | Negative | 6 | 4 | Southeast Asia | This study |
| BK11 | CSF | Clinical | Vietnam | Unknown | Positive | 32 | 31 | Southeast Asia | This study |
| BK17 | CSF | Clinical | Vietnam | Unknown | Positive | 32 | 31 | Southeast Asia | This study |
| BK27 | CSF | Clinical | Vietnam | Unknown | Positive | 32 | 31 | Southeast Asia | This study |
| BK46 | CSF | Clinical | Vietnam | Unknown | Positive | 32 | 31 | Southeast Asia | This study |
| NRHc5028.ENR.STOR | CSF | Clinical | Botswana | 2012 | Positive | 3 | 31 | Africa | Chen et al., (2016) |
| bt130 | Cerebrospinal fluid | Clinical | Botswana | 2001 | Positive | 13 | 31 | Africa | Litvintseva et al. Genetics. 2006,172(4):2223–2238 |
| bt104 | Cerebrospinal fluid | Clinical | Botswana | 2001 | Positive | 22 | 31 | Africa | Litvintseva et al. Genetics. 2006,172(4):2223–2238 |
| NRHc5036.ENR | CSF | Clinical | Botswana | 2012 | Positive | 77 | 31 | Africa | Chen et al., (2016) |
| NRHc5044.REL.INI.CLIN.ISO | CSF | Clinical | Botswana | 2012 | Positive | 77 | 31 | Africa | Chen et al., (2016) |
| PMHc1039.ENR.STOR | CSF | Clinical | Botswana | 2012 | Positive | 77 | 31 | Africa | Chen et al., (2016) |
| PMHc1018.CLIN.1 | CSF | Clinical | Botswana | 2012 | Positive | 32 | 31 | Africa | Chen et al., (2016) |
| HK_03 | Clinical | Clinical | China | Unknown | Unknown | 5 | 5 | East Asia | Khayhan et al., (2013) |
| CNS487 | CSF | Clinical | Laos | Unknown | Negative | 93 | 31 | Southeast Asia | This study |
| UFTM 14.132 | Cerebrospinal fluid | Clinical | Brazil | 2007 | Positive | 31 | 31 | South America | Ferreira-Paim et al, PlosNTD,2017 |
| UFTM 14.133 | Cerebrospinal fluid | Clinical | Brazil | 2007 | Positive | 31 | 31 | South America | Ferreira-Paim et al, PlosNTD,2017 |
| UFTM 14.113 | Cerebrospinal fluid | Clinical | Brazil | 2003 | Positive | 39 | 31 | South America | Ferreira-Paim et al, PlosNTD,2017 |
| UFTM 14.96 | Cerebrospinal fluid | Clinical | Brazil | 2002 | Positive | 32 | 31 | South America | Ferreira-Paim et al, PlosNTD,2017 |
| UFTM 14.140 | Cerebrospinal fluid | Clinical | Brazil | 2008 | Positive | 32 | 31 | South America | Ferreira-Paim et al, PlosNTD,2017 |
| UFTM 14.46 | Urine | Clinical | Brazil | 2006 | Positive | 93 | 31 | South America | Ferreira-Paim et al, PlosNTD,2017 |
| UFTM 14.47 | Urine | Clinical | Brazil | 2006 | Positive | 93 | 31 | South America | Ferreira-Paim et al, PlosNTD,2017 |
| UFTM 14.49 | Urine | Clinical | Brazil | 2006 | Positive | 93 | 31 | South America | Ferreira-Paim et al, PlosNTD,2017 |
| UFTM 14.50 | Urine | Clinical | Brazil | 2007 | Positive | 93 | 31 | South America | Ferreira-Paim et al, PlosNTD,2017 |
| UFTM 14.51 | Urine | Clinical | Brazil | 2005 | Positive | 93 | 31 | South America | Ferreira-Paim et al, PlosNTD,2017 |
| UFTM 14.52 | Urine | Clinical | Brazil | 2006 | Positive | 93 | 31 | South America | Ferreira-Paim et al, PlosNTD,2017 |
| UFTM 14.55 | Urine | Clinical | Brazil | 2012 | Positive | 93 | 31 | South America | Ferreira-Paim et al, PlosNTD,2017 |
| UFTM 14.61 | Blood | Clinical | Brazil | 2010 | Positive | 93 | 31 | South America | Ferreira-Paim et al, PlosNTD,2017 |
| UFTM 14.62 | Blood | Clinical | Brazil | 2009 | Positive | 93 | 31 | South America | Ferreira-Paim et al, PlosNTD,2017 |
| UFTM 14.64 | Blood | Clinical | Brazil | 2005 | Positive | 93 | 31 | South America | Ferreira-Paim et al, PlosNTD,2017 |
| UFTM 14.66 | Blood | Clinical | Brazil | 2012 | Positive | 93 | 31 | South America | Ferreira-Paim et al, PlosNTD,2017 |
| UFTM 14.67 | Blood | Clinical | Brazil | 2012 | Positive | 93 | 31 | South America | Ferreira-Paim et al, PlosNTD,2017 |
| UFTM 14.68 | Blood | Clinical | Brazil | 2012 | Positive | 93 | 31 | South America | Ferreira-Paim et al, PlosNTD,2017 |
| UFTM 14.69 | Blood | Clinical | Brazil | 2012 | Positive | 93 | 31 | South America | Ferreira-Paim et al, PlosNTD,2017 |
| UFTM 14.71 | Blood | Clinical | Brazil | 2013 | Positive | 93 | 31 | South America | Ferreira-Paim et al, PlosNTD,2017 |
| UFTM 14.73 | Blood | Clinical | Brazil | 2005 | Positive | 93 | 31 | South America | Ferreira-Paim et al, PlosNTD,2017 |
| UFTM 14.77 | Cerebrospinal fluid | Clinical | Brazil | 2011 | Positive | 93 | 31 | South America | Ferreira-Paim et al, PlosNTD,2017 |
| UFTM 14.80 | Cerebrospinal fluid | Clinical | Brazil | 2007 | Positive | 93 | 31 | South America | Ferreira-Paim et al, PlosNTD,2017 |
| UFTM 14.81 | Cerebrospinal fluid | Clinical | Brazil | 2011 | Positive | 93 | 31 | South America | Ferreira-Paim et al, PlosNTD,2017 |
| UFTM 14.84 | Cerebrospinal fluid | Clinical | Brazil | 2012 | Positive | 93 | 31 | South America | Ferreira-Paim et al, PlosNTD,2017 |
| UFTM 14.85 | Cerebrospinal fluid | Clinical | Brazil | 2012 | Positive | 93 | 31 | South America | Ferreira-Paim et al, PlosNTD,2017 |
| UFTM 14.89 | Cerebrospinal fluid | Clinical | Brazil | 2013 | Positive | 93 | 31 | South America | Ferreira-Paim et al, PlosNTD,2017 |
| UFTM 14.92 | Cerebrospinal fluid | Clinical | Brazil | 2014 | Positive | 93 | 31 | South America | Ferreira-Paim et al, PlosNTD,2017 |
| UFTM 14.93 | Cerebrospinal fluid | Clinical | Brazil | 2014 | Positive | 93 | 31 | South America | Ferreira-Paim et al, PlosNTD,2017 |
| UFTM 14.95 | Cerebrospinal fluid | Clinical | Brazil | 2014 | Positive | 93 | 31 | South America | Ferreira-Paim et al, PlosNTD,2017 |
| UFTM 14.97 | Cerebrospinal fluid | Clinical | Brazil | 2001 | Positive | 93 | 31 | South America | Ferreira-Paim et al, PlosNTD,2017 |
| UFTM 14.99 | Cerebrospinal fluid | Clinical | Brazil | 2002 | Positive | 93 | 31 | South America | Ferreira-Paim et al, PlosNTD,2017 |
| UFTM 14.100 | Cerebrospinal fluid | Clinical | Brazil | 2001 | Positive | 93 | 31 | South America | Ferreira-Paim et al, PlosNTD,2017 |
| UFTM 14.102 | Cerebrospinal fluid | Clinical | Brazil | 2003 | Positive | 93 | 31 | South America | Ferreira-Paim et al, PlosNTD,2017 |
| UFTM 14.104 | Cerebrospinal fluid | Clinical | Brazil | 1999 | Positive | 93 | 31 | South America | Ferreira-Paim et al, PlosNTD,2017 |
| UFTM 14.106 | Cerebrospinal fluid | Clinical | Brazil | 2003 | Positive | 93 | 31 | South America | Ferreira-Paim et al, PlosNTD,2017 |
| UFTM 14.107 | Cerebrospinal fluid | Clinical | Brazil | 2002 | Positive | 93 | 31 | South America | Ferreira-Paim et al, PlosNTD,2017 |
| UFTM 14.108 | Cerebrospinal fluid | Clinical | Brazil | 2001 | Positive | 93 | 31 | South America | Ferreira-Paim et al, PlosNTD,2017 |
| UFTM 14.115 | Cerebrospinal fluid | Clinical | Brazil | 2004 | Positive | 93 | 31 | South America | Ferreira-Paim et al, PlosNTD,2017 |
| UFTM 14.116 | Cerebrospinal fluid | Clinical | Brazil | 2003 | Positive | 93 | 31 | South America | Ferreira-Paim et al, PlosNTD,2017 |
| UFTM 14.119 | Cerebrospinal fluid | Clinical | Brazil | 1999 | Positive | 93 | 31 | South America | Ferreira-Paim et al, PlosNTD,2017 |
| UFTM 14.122 | Cerebrospinal fluid | Clinical | Brazil | 2006 | Positive | 93 | 31 | South America | Ferreira-Paim et al, PlosNTD,2017 |
| UFTM 14.123 | Cerebrospinal fluid | Clinical | Brazil | 2006 | Positive | 93 | 31 | South America | Ferreira-Paim et al, PlosNTD,2017 |
| UFTM 14.125 | Cerebrospinal fluid | Clinical | Brazil | 2006 | Positive | 93 | 31 | South America | Ferreira-Paim et al, PlosNTD,2017 |
| UFTM 14.126 | Cerebrospinal fluid | Clinical | Brazil | 2007 | Positive | 93 | 31 | South America | Ferreira-Paim et al, PlosNTD,2017 |
| UFTM 14.127 | Cerebrospinal fluid | Clinical | Brazil | 2007 | Positive | 93 | 31 | South America | Ferreira-Paim et al, PlosNTD,2017 |
| UFTM 14.128 | Cerebrospinal fluid | Clinical | Brazil | 2007 | Positive | 93 | 31 | South America | Ferreira-Paim et al, PlosNTD,2017 |
| UFTM 14.129 | Cerebrospinal fluid | Clinical | Brazil | 2007 | Positive | 93 | 31 | South America | Ferreira-Paim et al, PlosNTD,2017 |
| UFTM 14.131 | Cerebrospinal fluid | Clinical | Brazil | 2007 | Positive | 93 | 31 | South America | Ferreira-Paim et al, PlosNTD,2017 |
| UFTM 14.137 | Cerebrospinal fluid | Clinical | Brazil | 2007 | Positive | 93 | 31 | South America | Ferreira-Paim et al, PlosNTD,2017 |
| UFTM 14.138 | Cerebrospinal fluid | Clinical | Brazil | 2011 | Positive | 93 | 31 | South America | Ferreira-Paim et al, PlosNTD,2017 |
| UFTM 14.139 | Cerebrospinal fluid | Clinical | Brazil | 2009 | Positive | 93 | 31 | South America | Ferreira-Paim et al, PlosNTD,2017 |
| UFTM 14.142 | Cerebrospinal fluid | Clinical | Brazil | 2000 | Positive | 93 | 31 | South America | Ferreira-Paim et al, PlosNTD,2017 |
| UFTM 14.143 | Cerebrospinal fluid | Clinical | Brazil | 2009 | Positive | 93 | 31 | South America | Ferreira-Paim et al, PlosNTD,2017 |
| UFTM 14.144 | Cerebrospinal fluid | Clinical | Brazil | 2010 | Positive | 93 | 31 | South America | Ferreira-Paim et al, PlosNTD,2017 |
| UFTM 14.145 | Cerebrospinal fluid | Clinical | Brazil | 2009 | Positive | 93 | 31 | South America | Ferreira-Paim et al, PlosNTD,2017 |
| UFTM 14.146 | Cerebrospinal fluid | Clinical | Brazil | 2010 | Positive | 93 | 31 | South America | Ferreira-Paim et al, PlosNTD,2017 |
| UFTM 14.148 | Cerebrospinal fluid | Clinical | Brazil | 2000 | Positive | 93 | 31 | South America | Ferreira-Paim et al, PlosNTD,2017 |
| UFTM 14.149 | Cerebrospinal fluid | Clinical | Brazil | 2007 | Positive | 93 | 31 | South America | Ferreira-Paim et al, PlosNTD,2017 |
| UFTM 14.150 | Cerebrospinal fluid | Clinical | Brazil | 2003 | Positive | 93 | 31 | South America | Ferreira-Paim et al, PlosNTD,2017 |
| UFTM 14.151 | Cerebrospinal fluid | Clinical | Brazil | 2000 | Positive | 93 | 31 | South America | Ferreira-Paim et al, PlosNTD,2017 |
| UFTM 14.152 | Cerebrospinal fluid | Clinical | Brazil | 2001 | Positive | 93 | 31 | South America | Ferreira-Paim et al, PlosNTD,2017 |
| UFTM 14.156 | Cerebrospinal fluid | Clinical | Brazil | 2011 | Positive | 93 | 31 | South America | Ferreira-Paim et al, PlosNTD,2017 |
| UFTM 14.157 | Cerebrospinal fluid | Clinical | Brazil | 2010 | Positive | 93 | 31 | South America | Ferreira-Paim et al, PlosNTD,2017 |
| UFTM 14.158 | Cerebrospinal fluid | Clinical | Brazil | 2011 | Positive | 93 | 31 | South America | Ferreira-Paim et al, PlosNTD,2017 |
| UFTM 14.160 | Cerebrospinal fluid | Clinical | Brazil | 2011 | Positive | 93 | 31 | South America | Ferreira-Paim et al, PlosNTD,2017 |
| UFTM 14.162 | Cerebrospinal fluid | Clinical | Brazil | 2010 | Positive | 93 | 31 | South America | Ferreira-Paim et al, PlosNTD,2017 |
| UFTM 14.76 | Cerebrospinal fluid | Clinical | Brazil | 2003 | Positive | 540 | 31 | South America | Ferreira-Paim et al, PlosNTD,2017 |
| 09-0552 | Unknown | Clinical | Germany | 2009 | Positive | 32 | 31 | Europe | Sanchini et al. Med Microbiol Immunol. 2014,203(5):333-340 |
| 25_104 | CSF | Clinical | India | 1999 | Positive | 77 | 31 | South Asia | Khayhan et al., (2013) |
| 25_244 | CSF | Clinical | India | 2005 | Positive | 77 | 31 | South Asia | Khayhan et al., (2013) |
| 25_365 | CSF | Clinical | India | 2009 | Positive | 77 | 31 | South Asia | Khayhan et al., (2013) |
| 25_369 | CSF | Clinical | India | 2009 | Positive | 77 | 31 | South Asia | Khayhan et al., (2013) |
| 25_61 | CSF | Clinical | India | 1996 | Positive | 77 | 31 | South Asia | Khayhan et al., (2013) |
| 25_63 | CSF | Clinical | India | 1996 | Positive | 77 | 31 | South Asia | Khayhan et al., (2013) |
| 25_229 | CSF | Clinical | India | 2005 | Positive | 31 | 31 | South Asia | Khayhan et al., (2013) |
| 25_357 | CSF | Clinical | India | 2009 | Positive | 31 | 31 | South Asia | Khayhan et al., (2013) |
| 25_370 | CSF | Clinical | India | 2009 | Positive | 31 | 31 | South Asia | Khayhan et al., (2013) |
| in2629 | Cerebrospinal fluid | Clinical | India | 2001 | Positive | 38 | 31 | South Asia | Litvintseva et al. Genetics. 2006,172(4):2223–2238 |
| 25_228 | CSF | Clinical | India | 2005 | Positive | 93 | 31 | South Asia | Khayhan et al., (2013) |
| 25_237 | CSF | Clinical | India | 2005 | Positive | 93 | 31 | South Asia | Khayhan et al., (2013) |
| 25_239 | CSF | Clinical | India | 1999 | Positive | 93 | 31 | South Asia | Khayhan et al., (2013) |
| 25_261 | CSF | Clinical | India | 2005 | Positive | 93 | 31 | South Asia | Khayhan et al., (2013) |
| 25_272 | Bronchoalveolar lavage | Clinical | India | 2006 | Positive | 93 | 31 | South Asia | Khayhan et al., (2013) |
| 25_277 | CSF | Clinical | India | 2006 | Positive | 93 | 31 | South Asia | Khayhan et al., (2013) |
| 25_290 | CSF | Clinical | India | 2006 | Positive | 93 | 31 | South Asia | Khayhan et al., (2013) |
| 25_292 | CSF | Clinical | India | 2006 | Positive | 93 | 31 | South Asia | Khayhan et al., (2013) |
| 25_302 | CSF | Clinical | India | 2007 | Positive | 93 | 31 | South Asia | Khayhan et al., (2013) |
| 25_304 | CSF | Clinical | India | 2007 | Positive | 93 | 31 | South Asia | Khayhan et al., (2013) |
| 25_308 | CSF | Clinical | India | 2007 | Positive | 93 | 31 | South Asia | Khayhan et al., (2013) |
| 25_312 | CSF | Clinical | India | 2007 | Positive | 93 | 31 | South Asia | Khayhan et al., (2013) |
| 25_313 | CSF | Clinical | India | 2007 | Positive | 93 | 31 | South Asia | Khayhan et al., (2013) |
| 25_334 | CSF | Clinical | India | 2008 | Positive | 93 | 31 | South Asia | Khayhan et al., (2013) |
| 25_336 | CSF | Clinical | India | 2008 | Positive | 93 | 31 | South Asia | Khayhan et al., (2013) |
| 25_337 | CSF | Clinical | India | 2008 | Positive | 93 | 31 | South Asia | Khayhan et al., (2013) |
| 25_341 | CSF | Clinical | India | 2008 | Positive | 93 | 31 | South Asia | Khayhan et al., (2013) |
| 25_344 | Bronchoalveolar lavage | Clinical | India | 2008 | Positive | 93 | 31 | South Asia | Khayhan et al., (2013) |
| 25_356 | CSF | Clinical | India | 2009 | Positive | 93 | 31 | South Asia | Khayhan et al., (2013) |
| 25_358 | CSF | Clinical | India | 2005 | Positive | 93 | 31 | South Asia | Khayhan et al., (2013) |
| 25_367 | CSF | Clinical | India | 2009 | Positive | 93 | 31 | South Asia | Khayhan et al., (2013) |
| 25_368 | CSF | Clinical | India | 2009 | Positive | 93 | 31 | South Asia | Khayhan et al., (2013) |
| 132 | CSF | Clinical | Indonesia | 2007 | Positive | 93 | 31 | Southeast Asia | Khayhan et al., (2013) |
| 597 | CSF | Clinical | Indonesia | 2006 | Positive | 93 | 31 | Southeast Asia | Khayhan et al., (2013) |
| 676 | CSF | Clinical | Indonesia | 2006 | Positive | 93 | 31 | Southeast Asia | Khayhan et al., (2013) |
| 1019 | CSF | Clinical | Indonesia | 2005 | Positive | 93 | 31 | Southeast Asia | Khayhan et al., (2013) |
| 1051 | CSF | Clinical | Indonesia | 2006 | Positive | 93 | 31 | Southeast Asia | Khayhan et al., (2013) |
| 1116 | CSF | Clinical | Indonesia | 2006 | Positive | 93 | 31 | Southeast Asia | Khayhan et al., (2013) |
| 1200 | CSF | Clinical | Indonesia | Unknown | Positive | 93 | 31 | Southeast Asia | Khayhan et al., (2013) |
| 1206 | CSF | Clinical | Indonesia | 2006 | Positive | 93 | 31 | Southeast Asia | Khayhan et al., (2013) |
| 1336 | CSF | Clinical | Indonesia | 2006 | Positive | 93 | 31 | Southeast Asia | Khayhan et al., (2013) |
| 1462 | CSF | Clinical | Indonesia | 2006 | Positive | 93 | 31 | Southeast Asia | Khayhan et al., (2013) |
| 1571 | CSF | Clinical | Indonesia | 2006 | Positive | 93 | 31 | Southeast Asia | Khayhan et al., (2013) |
| 2126 | CSF | Clinical | Indonesia | Unknown | Positive | 93 | 31 | Southeast Asia | Khayhan et al., (2013) |
| 3400 | CSF | Clinical | Indonesia | 2005 | Positive | 93 | 31 | Southeast Asia | Khayhan et al., (2013) |
| 3594 | CSF | Clinical | Indonesia | 2006 | Positive | 93 | 31 | Southeast Asia | Khayhan et al., (2013) |
| 3634 | CSF | Clinical | Indonesia | 2006 | Positive | 93 | 31 | Southeast Asia | Khayhan et al., (2013) |
| Jakarta 1051 | CSF | Clinical | Indonesia | 2006 | Positive | 93 | 31 | Southeast Asia | Khayhan et al., (2013) |
| CR2231 | CSF | Clinical | Indonesia | 2004 | Positive | 177 | 31 | Southeast Asia | Khayhan et al., (2013) |
| CR755 | CSF | Clinical | Indonesia | 2004 | Positive | 177 | 31 | Southeast Asia | Khayhan et al., (2013) |
| IUM 99-5690 | Cerebrospinal fluid | Clinical | Italy | #N/A | Positive | 59 | 31 | Europe | Cogliati et al. Medical Mycology. 2013,51:499–506 |
| IUM 02-2304 | Cerebrospinal fluid | Clinical | Italy | #N/A | Positive | 75 | 31 | Europe | Cogliati et al. Medical Mycology. 2013,51:499–506 |
| K54 | Cerebrospinal fluid | Clinical | Korea | #N/A | Positive | 312 | 31 | East Asia | Choi et al. FEMS Yeast Res. 2010,10(6):769–778 |
| 481_03 | CSF | Clinical | Kuwait | 2003 | Positive | 93 | 31 | Middle East | Khayhan et al., (2013) |
| CNS1465 | CSF | Clinical | Laos | 2014 | Positive | 187 | 31 | Southeast Asia | This study |
| mal 9 | Blood | Clinical | Malawi | #N/A | Positive | 30 | 31 | Africa | Litvintseva et al. Genetics. 2006,172(4):2223–2238 |
| 257 | CSF | Clinical | South Africa |  | Positive | 3 | 31 | Africa | Beale MA et al, Plos NTD,2015 |
| 370 | CSF | Clinical | South Africa |  | Positive | 31 | 31 | Africa | Beale MA et al, Plos NTD,2015 |
| 469 | CSF | Clinical | South Africa |  | Positive | 31 | 31 | Africa | Beale MA et al, Plos NTD,2015 |
| 471 | CSF | Clinical | South Africa |  | Positive | 31 | 31 | Africa | Beale MA et al, Plos NTD,2015 |
| 106 | CSF | Clinical | South Africa |  | Positive | 32 | 31 | Africa | Beale MA et al, Plos NTD,2015 |
| 109 | CSF | Clinical | South Africa |  | Positive | 32 | 31 | Africa | Beale MA et al, Plos NTD,2015 |
| 116 | CSF | Clinical | South Africa |  | Positive | 32 | 31 | Africa | Beale MA et al, Plos NTD,2015 |
| 121 | CSF | Clinical | South Africa |  | Positive | 32 | 31 | Africa | Beale MA et al, Plos NTD,2015 |
| 122 | CSF | Clinical | South Africa |  | Positive | 32 | 31 | Africa | Beale MA et al, Plos NTD,2015 |
| 123 | CSF | Clinical | South Africa |  | Positive | 32 | 31 | Africa | Beale MA et al, Plos NTD,2015 |
| 124 | CSF | Clinical | South Africa |  | Positive | 32 | 31 | Africa | Beale MA et al, Plos NTD,2015 |
| 136 | CSF | Clinical | South Africa |  | Positive | 32 | 31 | Africa | Beale MA et al, Plos NTD,2015 |
| 137 | CSF | Clinical | South Africa |  | Positive | 32 | 31 | Africa | Beale MA et al, Plos NTD,2015 |
| 143 | CSF | Clinical | South Africa |  | Positive | 32 | 31 | Africa | Beale MA et al, Plos NTD,2015 |
| 243 | CSF | Clinical | South Africa |  | Positive | 32 | 31 | Africa | Beale MA et al, Plos NTD,2015 |
| 238 | CSF | Clinical | South Africa |  | Positive | 32 | 31 | Africa | Beale MA et al, Plos NTD,2015 |
| 247 | CSF | Clinical | South Africa |  | Positive | 32 | 31 | Africa | Beale MA et al, Plos NTD,2015 |
| 252 | CSF | Clinical | South Africa |  | Positive | 32 | 31 | Africa | Beale MA et al, Plos NTD,2015 |
| 369 | CSF | Clinical | South Africa |  | Positive | 32 | 31 | Africa | Beale MA et al, Plos NTD,2015 |
| 346 | CSF | Clinical | South Africa |  | Positive | 32 | 31 | Africa | Beale MA et al, Plos NTD,2015 |
| 463 | CSF | Clinical | South Africa |  | Positive | 32 | 31 | Africa | Beale MA et al, Plos NTD,2015 |
| 360 | CSF | Clinical | South Africa |  | Positive | 93 | 31 | Africa | Beale MA et al, Plos NTD,2015 |
| 301 | CSF | Clinical | South Africa |  | Positive | 93 | 31 | Africa | Beale MA et al, Plos NTD,2015 |
| 328 | CSF | Clinical | South Africa |  | Positive | 93 | 31 | Africa | Beale MA et al, Plos NTD,2015 |
| 333 | CSF | Clinical | South Africa |  | Positive | 93 | 31 | Africa | Beale MA et al, Plos NTD,2015 |
| 367 | CSF | Clinical | South Africa |  | Positive | 93 | 31 | Africa | Beale MA et al, Plos NTD,2015 |
| 410 | CSF | Clinical | South Africa |  | Positive | 93 | 31 | Africa | Beale MA et al, Plos NTD,2015 |
| 419 | CSF | Clinical | South Africa |  | Positive | 93 | 31 | Africa | Beale MA et al, Plos NTD,2015 |
| 312 | CSF | Clinical | South Africa |  | Positive | 93 | 31 | Africa | Beale MA et al, Plos NTD,2015 |
| 319 | CSF | Clinical | South Africa |  | Positive | 93 | 31 | Africa | Beale MA et al, Plos NTD,2015 |
| 324 | CSF | Clinical | South Africa |  | Positive | 93 | 31 | Africa | Beale MA et al, Plos NTD,2015 |
| 330 | CSF | Clinical | South Africa |  | Positive | 93 | 31 | Africa | Beale MA et al, Plos NTD,2015 |
| 344 | CSF | Clinical | South Africa |  | Positive | 93 | 31 | Africa | Beale MA et al, Plos NTD,2015 |
| 345 | CSF | Clinical | South Africa |  | Positive | 93 | 31 | Africa | Beale MA et al, Plos NTD,2015 |
| 348 | CSF | Clinical | South Africa |  | Positive | 93 | 31 | Africa | Beale MA et al, Plos NTD,2015 |
| 358 | CSF | Clinical | South Africa |  | Positive | 93 | 31 | Africa | Beale MA et al, Plos NTD,2015 |
| 373 | CSF | Clinical | South Africa |  | Positive | 93 | 31 | Africa | Beale MA et al, Plos NTD,2015 |
| 304 | CSF | Clinical | South Africa |  | Positive | 93 | 31 | Africa | Beale MA et al, Plos NTD,2015 |
| 322 | CSF | Clinical | South Africa |  | Positive | 93 | 31 | Africa | Beale MA et al, Plos NTD,2015 |
| 327 | CSF | Clinical | South Africa |  | Positive | 93 | 31 | Africa | Beale MA et al, Plos NTD,2015 |
| 338 | CSF | Clinical | South Africa |  | Positive | 93 | 31 | Africa | Beale MA et al, Plos NTD,2015 |
| 342 | CSF | Clinical | South Africa |  | Positive | 93 | 31 | Africa | Beale MA et al, Plos NTD,2015 |
| 355 | CSF | Clinical | South Africa |  | Positive | 93 | 31 | Africa | Beale MA et al, Plos NTD,2015 |
| 359 | CSF | Clinical | South Africa |  | Positive | 93 | 31 | Africa | Beale MA et al, Plos NTD,2015 |
| 366 | CSF | Clinical | South Africa |  | Positive | 93 | 31 | Africa | Beale MA et al, Plos NTD,2015 |
| 368 | CSF | Clinical | South Africa |  | Positive | 93 | 31 | Africa | Beale MA et al, Plos NTD,2015 |
| 380 | CSF | Clinical | South Africa |  | Positive | 93 | 31 | Africa | Beale MA et al, Plos NTD,2015 |
| 320 | CSF | Clinical | South Africa |  | Positive | 93 | 31 | Africa | Beale MA et al, Plos NTD,2015 |
| 363 | CSF | Clinical | South Africa |  | Positive | 93 | 31 | Africa | Beale MA et al, Plos NTD,2015 |
| 375 | CSF | Clinical | South Africa |  | Positive | 93 | 31 | Africa | Beale MA et al, Plos NTD,2015 |
| 417 | CSF | Clinical | South Africa |  | Positive | 93 | 31 | Africa | Beale MA et al, Plos NTD,2015 |
| 459 | CSF | Clinical | South Africa |  | Positive | 93 | 31 | Africa | Beale MA et al, Plos NTD,2015 |
| 477 | CSF | Clinical | South Africa |  | Positive | 93 | 31 | Africa | Beale MA et al, Plos NTD,2015 |
| 486 | CSF | Clinical | South Africa |  | Positive | 93 | 31 | Africa | Beale MA et al, Plos NTD,2015 |
| 444 | CSF | Clinical | South Africa |  | Positive | 185 | 31 | Africa | Beale MA et al, Plos NTD,2015 |
| 217 | CSF | Clinical | South Africa |  | Positive | 199 | 31 | Africa | Beale MA et al, Plos NTD,2015 |
| 261 | CSF | Clinical | South Africa |  | Positive | 199 | 31 | Africa | Beale MA et al, Plos NTD,2015 |
| 244 | CSF | Clinical | South Africa |  | Positive | 199 | 31 | Africa | Beale MA et al, Plos NTD,2015 |
| 141 | CSF | Clinical | South Africa |  | Positive | 200 | 31 | Africa | Beale MA et al, Plos NTD,2015 |
| 329 | CSF | Clinical | South Africa |  | Positive | 236 | 31 | Africa | Beale MA et al, Plos NTD,2015 |
| 110 | CSF | Clinical | South Africa |  | Positive | 237 | 31 | Africa | Beale MA et al, Plos NTD,2015 |
| 354 | CSF | Clinical | South Africa |  | Positive | 238 | 31 | Africa | Beale MA et al, Plos NTD,2015 |
| 406 | CSF | Clinical | South Africa |  | Positive | 239 | 31 | Africa | Beale MA et al, Plos NTD,2015 |
| 140 | CSF | Clinical | South Africa |  | Positive | 240 | 31 | Africa | Beale MA et al, Plos NTD,2015 |
| 336 | CSF | Clinical | South Africa |  | Positive | 241 | 31 | Africa | Beale MA et al, Plos NTD,2015 |
| Tn10 | Blood | Clinical | Tanzania | #N/A | Positive | 32 | 31 | Africa | Litvintseva et al. Genetics. 2006,172(4):2223–2238 |
| WH037 | CSF | Clinical | China | 2003 | Negative | 31 | 31 | East Asia | Khayhan et al., (2013) |
| 9256 | Avian guano | Environmental | Japan | 2005 | NA | 31 | 31 | East Asia | Khayhan et al., (2013) |
| HK_04 | Clinical | Clinical | China | Unknown | Unknown | 5 | 5 | East Asia | Khayhan et al., (2013) |
| WH001 | CSF | Clinical | China | 2000 | Negative | 93 | 31 | East Asia | Khayhan et al., (2013) |
| WH069 | CSF | Clinical | China | 2001 | Negative | 191 | 31 | East Asia | Khayhan et al., (2013) |
| HK_05 | Clinical | Clinical | China | Unknown | Unknown | 5 | 5 | East Asia | Khayhan et al., (2013) |
| blg11 | Avian guano | Enviromental | Belgium | Unknown | NA | 32 | 31 | Europe | Litvintseva et al. Genetics. 2006,172(4):2223–2238 |
| HK_06 | Clinical | Clinical | China | Unknown | Unknown | 5 | 5 | East Asia | Khayhan et al., (2013) |
| 1605202443 | CSF | Clinical | Qatar | 2005 | Negative | 31 | 31 | Middle East | Khayhan et al., (2013) |
| 1608000352 | CSF | Clinical | Qatar | 2008 | Negative | 31 | 31 | Middle East | Khayhan et al., (2013) |
| 177_02 | Wound swab | Clinical | Kuwait | 2002 | Negative | 185 | 31 | Middle East | Khayhan et al., (2013) |
| A2 102-5 | Avian guano | Enviromental | USA | 2003 | NA | 15 | 31 | North America | Litvintseva et al. Genetics. 2006,172(4):2223–2238; Litvintseva et al. PlosOne. 2011, 6(5):e19688 |
| JS18 | Veterinay | Veterinary | USA | #N/A | NA | 39 | 31 | North America | Singer et al. J Clin Microbiol. 2014, 52(6):2061-2070 |
| JS71 | Veterinay | Veterinary | USA | #N/A | NA | 32 | 31 | North America | Singer et al. J Clin Microbiol. 2014, 52(6):2061-2070 |
| UFTM 14.09 | Enviromental | Enviromental | Brazil | #N/A | NA | 15 | 31 | South America | Ferreira-paim et. al. Mycoses. 2011, 54(5):e294-300 |
| UFTM 14.44 | Enviromental | Enviromental | Brazil | Unknown | NA | 15 | 31 | South America | Ferreira-Paim et al, PlosNTD,2017 |
| UFTM 14.10 | Enviromental | Enviromental | Brazil | #N/A | NA | 31 | 31 | South America | Ferreira-paim et. al. Mycoses. 2011, 54(5):e294-300 |
| UFTM 14.94 | Cerebrospinal fluid | Clinical | Brazil | 2013 | Kidney transplant/Diabets mellitus | 39 | 31 | South America | Ferreira-Paim et al, PlosNTD,2017 |
| UFTM 14.130 | Cerebrospinal fluid | Clinical | Brazil | 2007 | Crohn's disease | 39 | 31 | South America | Ferreira-Paim et al, PlosNTD,2017 |
| HK_07 | Clinical | Clinical | China | Unknown | Unknown | 5 | 5 | East Asia | Khayhan et al., (2013) |
| UFTM 14.02 | Enviromental | Enviromental | Brazil | #N/A | NA | 93 | 31 | South America | Ferreira-paim et. al. Mycoses. 2011, 54(5):e294-300 |
| UFTM 14.03 | Enviromental | Enviromental | Brazil | #N/A | NA | 93 | 31 | South America | Ferreira-paim et. al. Mycoses. 2011, 54(5):e294-300 |
| UFTM 14.04 | Enviromental | Enviromental | Brazil | #N/A | NA | 93 | 31 | South America | Ferreira-paim et. al. Mycoses. 2011, 54(5):e294-300 |
| UFTM 14.07 | Enviromental | Enviromental | Brazil | #N/A | NA | 93 | 31 | South America | Ferreira-paim et. al. Mycoses. 2011, 54(5):e294-300 |
| UFTM 14.15 | Enviromental | Enviromental | Brazil | #N/A | NA | 93 | 31 | South America | Ferreira-Paim et al, PlosNTD,2017 |
| UFTM 14.16 | Enviromental | Enviromental | Brazil | #N/A | NA | 93 | 31 | South America | Ferreira-Paim et al, PlosNTD,2017 |
| UFTM 14.25 | Enviromental | Enviromental | Brazil | #N/A | NA | 93 | 31 | South America | Ferreira-Paim et al, PlosNTD,2017 |
| UFTM 14.27 | Enviromental | Enviromental | Brazil | #N/A | NA | 93 | 31 | South America | Ferreira-Paim et al, PlosNTD,2017 |
| UFTM 14.29 | Enviromental | Enviromental | Brazil | #N/A | NA | 93 | 31 | South America | Ferreira-Paim et al, PlosNTD,2017 |
| UFTM 14.39 | Enviromental | Enviromental | Brazil | #N/A | NA | 93 | 31 | South America | Ferreira-Paim et al, PlosNTD,2017 |
| UFTM 14.58 | Skin | Clinical | Brazil | 2013 | Kidney transplant | 93 | 31 | South America | Ferreira-Paim et al, PlosNTD,2017 |
| UFTM 14.65 | Blood | Clinical | Brazil | 2006 | Diabets mellitus | 93 | 31 | South America | Ferreira-Paim et al, PlosNTD,2017 |
| HK_08 | Clinical | Clinical | China | Unknown | Unknown | 5 | 5 | East Asia | Khayhan et al., (2013) |
| UFTM 14.101 | Cerebrospinal fluid | Clinical | Brazil | 2002 | Systemic Lupus Erythematosus | 93 | 31 | South America | Ferreira-Paim et al, PlosNTD,2017 |
| HK_09 | Clinical | Clinical | China | Unknown | Unknown | 5 | 5 | East Asia | Khayhan et al., (2013) |
| UFTM 14.111 | Cerebrospinal fluid | Clinical | Brazil | 2004 | Diabets mellitus/Nefritis | 93 | 31 | South America | Ferreira-Paim et al, PlosNTD,2017 |
| HK_10 | Clinical | Clinical | China | Unknown | Unknown | 5 | 5 | East Asia | Khayhan et al., (2013) |
| HK_11 | Clinical | Clinical | China | Unknown | Unknown | 5 | 5 | East Asia | Khayhan et al., (2013) |
| HK_12 | Clinical | Clinical | China | Unknown | Unknown | 5 | 5 | East Asia | Khayhan et al., (2013) |
| UFTM 14.06 | Enviromental | Enviromental | Brazil | #N/A | NA | 77 | 31 | South America | Ferreira-paim et. al. Mycoses. 2011, 54(5):e294-300 |
| UFTM 14.08 | Enviromental | Enviromental | Brazil | #N/A | NA | 77 | 31 | South America | Ferreira-paim et. al. Mycoses. 2011, 54(5):e294-300 |
| UFTM 14.11 | Enviromental | Enviromental | Brazil | #N/A | NA | 77 | 31 | South America | Ferreira-paim et. al. Mycoses. 2011, 54(5):e294-300 |
| UFTM 14.13 | Enviromental | Enviromental | Brazil | #N/A | NA | 77 | 31 | South America | Ferreira-paim et. al. Mycoses. 2011, 54(5):e294-300 |
| UFTM 14.17 | Enviromental | Enviromental | Brazil | #N/A | NA | 77 | 31 | South America | Ferreira-Paim et al, PlosNTD,2017 |
| UFTM 14.18 | Enviromental | Enviromental | Brazil | #N/A | NA | 77 | 31 | South America | Ferreira-Paim et al, PlosNTD,2017 |
| UFTM 14.19 | Enviromental | Enviromental | Brazil | #N/A | NA | 77 | 31 | South America | Ferreira-Paim et al, PlosNTD,2017 |
| UFTM 14.22 | Enviromental | Enviromental | Brazil | #N/A | NA | 77 | 31 | South America | Ferreira-Paim et al, PlosNTD,2017 |
| UFTM 14.23 | Enviromental | Enviromental | Brazil | #N/A | NA | 77 | 31 | South America | Ferreira-Paim et al, PlosNTD,2017 |
| UFTM 14.24 | Enviromental | Enviromental | Brazil | #N/A | NA | 77 | 31 | South America | Ferreira-Paim et al, PlosNTD,2017 |
| UFTM 14.26 | Enviromental | Enviromental | Brazil | #N/A | NA | 77 | 31 | South America | Ferreira-Paim et al, PlosNTD,2017 |
| UFTM 14.30 | Enviromental | Enviromental | Brazil | #N/A | NA | 77 | 31 | South America | Ferreira-Paim et al, PlosNTD,2017 |
| UFTM 14.31 | Enviromental | Enviromental | Brazil | #N/A | NA | 77 | 31 | South America | Ferreira-Paim et al, PlosNTD,2017 |
| UFTM 14.33 | Enviromental | Enviromental | Brazil | #N/A | NA | 77 | 31 | South America | Ferreira-Paim et al, PlosNTD,2017 |
| UFTM 14.34 | Enviromental | Enviromental | Brazil | #N/A | NA | 77 | 31 | South America | Ferreira-Paim et al, PlosNTD,2017 |
| UFTM 14.36 | Enviromental | Enviromental | Brazil | #N/A | NA | 77 | 31 | South America | Ferreira-Paim et al, PlosNTD,2017 |
| UFTM 14.37 | Enviromental | Enviromental | Brazil | #N/A | NA | 77 | 31 | South America | Ferreira-Paim et al, PlosNTD,2017 |
| 25_105 | CSF | Clinical | India | 2000 | Negative | 77 | 31 | South Asia | Khayhan et al., (2013) |
| 25_110 | CSF | Clinical | India | 1987 | Negative | 77 | 31 | South Asia | Khayhan et al., (2013) |
| 25_52 | CSF | Clinical | India | 1996 | Negative | 77 | 31 | South Asia | Khayhan et al., (2013) |
| 25_53 | CSF | Clinical | India | 1996 | Negative | 77 | 31 | South Asia | Khayhan et al., (2013) |
| 25_62 | CSF | Clinical | India | 1996 | Negative | 77 | 31 | South Asia | Khayhan et al., (2013) |
| 25_84 | Blood | Clinical | India | 1998 | Negative | 77 | 31 | South Asia | Khayhan et al., (2013) |
| HK_13 | Clinical | Clinical | China | Unknown | Unknown | 5 | 5 | East Asia | Khayhan et al., (2013) |
| HK_14 | Clinical | Clinical | China | Unknown | Unknown | 5 | 5 | East Asia | Khayhan et al., (2013) |
| 25_18 | CSF | Clinical | India | 1989 | Negative | 31 | 31 | South Asia | Khayhan et al., (2013) |
| 25_266 | CSF | Clinical | India | 2006 | Negative | 31 | 31 | South Asia | Khayhan et al., (2013) |
| 25_298 | CSF | Clinical | India | 2007 | Negative | 31 | 31 | South Asia | Khayhan et al., (2013) |
| 25_355 | CSF | Clinical | India | 2009 | Negative | 31 | 31 | South Asia | Khayhan et al., (2013) |
| 25_299 | CSF | Clinical | India | 2007 | Negative | 93 | 31 | South Asia | Khayhan et al., (2013) |
| 25_339 | CSF | Clinical | India | 2008 | Negative | 93 | 31 | South Asia | Khayhan et al., (2013) |
| 25_371 | CSF | Clinical | India | 2009 | Negative | 93 | 31 | South Asia | Khayhan et al., (2013) |
| 25_49 | CSF | Clinical | India | 1995 | Negative | 93 | 31 | South Asia | Khayhan et al., (2013) |
| CM35 | CSF | Clinical | Thailand | 2002 | Positive | 93 | 31 | Southeast Asia | Khayhan et al., (2013) |
| CN5007 | CSF | Clinical | Thailand | 2007 | Positive | 93 | 31 | Southeast Asia | Khayhan et al., (2013) |
| 25_50 | CSF | Clinical | India | 1995 | Negative | 93 | 31 | South Asia | Khayhan et al., (2013) |
| 25_86 | CSF | Clinical | India | 1998 | Negative | 93 | 31 | South Asia | Khayhan et al., (2013) |
| HLJ2 | Blood | Clinical | China | Unknown | Unknown | 63 | 63 | East Asia | Wu et al. Mycoses. 2015,58(5):280-287 |
| 25_33 | CSF | Clinical | India | 1991 | Negative | 177 | 31 | South Asia | Khayhan et al., (2013) |
| 25_340 | CSF | Clinical | India | 2008 | Negative | 187 | 31 | South Asia | Khayhan et al., (2013) |
| D15 | Avian guano | Environmental | Thailand | 2000 | NA | 31 | 31 | Southeast Asia | Khayhan et al., (2013) |
| D17 | Avian guano | Environmental | Thailand | 2000 | NA | 31 | 31 | Southeast Asia | Khayhan et al., (2013) |
| D19 | Avian guano | Environmental | Thailand | 2000 | NA | 31 | 31 | Southeast Asia | Khayhan et al., (2013) |
| UgCl076 | Cerebrospinal fluid | Clinical | Uganda | #N/A | Positive | 31 | 31 | Africa | Wiesner et al. mBio. 2012,3(5):e00196-12 |
| UgCl037 | Cerebrospinal fluid | Clinical | Uganda | #N/A | Positive | 39 | 31 | Africa | Wiesner et al. mBio. 2012,3(5):e00196-12 |
| ug2458 | Cerebrospinal fluid | Clinical | Uganda | #N/A | Positive | 32 | 31 | Africa | Litvintseva et al. Genetics. 2006,172(4):2223–2238 |
| UgCl040 | Cerebrospinal fluid | Clinical | Uganda | #N/A | Positive | 78 | 31 | Africa | Wiesner et al. mBio. 2012,3(5):e00196-12 |
| BK68 | CSF | Clinical | Vietnam | Unknown | Positive | 32 | 31 | Southeast Asia | This study |
| BMD915 | CSF | Clinical | Vietnam | Unknown | Negative | 32 | 31 | Southeast Asia | This study |
| UgCl107 | Cerebrospinal fluid | Clinical | Uganda | #N/A | Positive | 91 | 31 | Africa | Wiesner et al. mBio. 2012,3(5):e00196-12 |
| UgCl122 | Cerebrospinal fluid | Clinical | Uganda | #N/A | Positive | 92 | 31 | Africa | Wiesner et al. mBio. 2012,3(5):e00196-12 |
| UgCl001 | Cerebrospinal fluid | Clinical | Uganda | #N/A | Positive | 93 | 31 | Africa | Wiesner et al. mBio. 2012,3(5):e00196-12 |
| UgCl011 | Cerebrospinal fluid | Clinical | Uganda | #N/A | Positive | 77 | 31 | Africa | Wiesner et al. mBio. 2012,3(5):e00196-12 |
| UgCl036 | Cerebrospinal fluid | Clinical | Uganda | #N/A | Positive | 94 | 31 | Africa | Wiesner et al. mBio. 2012,3(5):e00196-12 |
| UgCl074 | Cerebrospinal fluid | Clinical | Uganda | #N/A | Positive | 95 | 31 | Africa | Wiesner et al. mBio. 2012,3(5):e00196-12 |
| Isolate1 | Unknown | Unknown | France | Unknown | Unknown | 320 | 31 | Europe | http://mlst.mycologylab.org/ |
| Isolate2 | Unknown | Unknown | France | Unknown | Unknown | 321 | 63 | Europe | http://mlst.mycologylab.org/ |
| 1291_09 | Blood | Clinical | Thailand | Unknown | Negative | 93 | 31 | Southeast Asia | Khayhan et al., (2013) |
| CR0E | CSF | Clinical | Indonesia | Unknown | Negative | 177 | 31 | Southeast Asia | Khayhan et al., (2013) |
| 110A | Avian guano | Environmental | Thailand | Unknown | NA | 185 | 31 | Southeast Asia | Khayhan et al., (2013) |
| Isolate3 | Unknown | Unknown | France | Unknown | Unknown | 322 | 63 | Europe | http://mlst.mycologylab.org/ |
| BMD942 | CSF | Clinical | Vietnam | Unknown | Negative | 32 | 31 | Southeast Asia | This study |
| BK154 | CSF | Clinical | Vietnam | Unknown | Positive | 39 | 31 | Southeast Asia | This study |
| BK167 | CSF | Clinical | Vietnam | Unknown | Positive | 39 | 31 | Southeast Asia | This study |
| BK209 | CSF | Clinical | Vietnam | Unknown | Positive | 39 | 31 | Southeast Asia | This study |
| BK12 | CSF | Clinical | Vietnam | Unknown | Positive | 93 | 31 | Southeast Asia | This study |
| BK157 | CSF | Clinical | Vietnam | Unknown | Positive | 93 | 31 | Southeast Asia | This study |
| BK179 | CSF | Clinical | Vietnam | Unknown | Positive | 93 | 31 | Southeast Asia | This study |
| BK213 | CSF | Clinical | Vietnam | Unknown | Positive | 93 | 31 | Southeast Asia | This study |
| BK228 | CSF | Clinical | Vietnam | Unknown | Positive | 93 | 31 | Southeast Asia | This study |
| BK24 | CSF | Clinical | Vietnam | Unknown | Positive | 93 | 31 | Southeast Asia | This study |
| BK33 | CSF | Clinical | Vietnam | Unknown | Positive | 93 | 31 | Southeast Asia | This study |
| BK85 | CSF | Clinical | Vietnam | Unknown | Positive | 93 | 31 | Southeast Asia | This study |
| BK109 | CSF | Clinical | Vietnam | Unknown | Positive | 137 | 4 | Southeast Asia | This study |
| BK172 | CSF | Clinical | Vietnam | Unknown | Positive | 188 | 4 | Southeast Asia | This study |
| BK64 | CSF | Clinical | Vietnam | Unknown | Positive | 188 | 4 | Southeast Asia | This study |
| BK129 | CSF | Clinical | Vietnam | Unknown | Positive | 195 | N/A | Southeast Asia | This study |
| BMD1367 | CSF | Clinical | Vietnam | Unknown | Negative | 306 | 4 | Southeast Asia | This study |
| BK150 | CSF | Clinical | Vietnam | Unknown | Positive | 338 | 31 | Southeast Asia | This study |
| BK153 | CSF | Clinical | Vietnam | Unknown | Positive | 339 | 31 | Southeast Asia | This study |
| za1346 | Cerebrospinal fluid | Clinical | Zaire | #N/A | Positive | 32 | 31 | Africa | Litvintseva et al. Genetics. 2006,172(4):2223–2238 |
| UFTM 14.63 | Cerebrospinal fluid | Clinical | Brazil | 2001 | Positive | 23 | 63 | South America | Ferreira-Paim et al, PlosNTD,2017 |
| UFTM 14.70 | Blood | Clinical | Brazil | 2013 | Positive | 23 | 63 | South America | Ferreira-Paim et al, PlosNTD,2017 |
| UFTM 14.72 | Blood | Clinical | Brazil | 2014 | Positive | 23 | 63 | South America | Ferreira-Paim et al, PlosNTD,2017 |
| UFTM 14.87 | Cerebrospinal fluid | Clinical | Brazil | 2008 | Positive | 23 | 63 | South America | Ferreira-Paim et al, PlosNTD,2017 |
| UFTM 14.103 | Cerebrospinal fluid | Clinical | Brazil | 2002 | Positive | 23 | 63 | South America | Ferreira-Paim et al, PlosNTD,2017 |
| UFTM 14.117 | Cerebrospinal fluid | Clinical | Brazil | 2004 | Positive | 23 | 63 | South America | Ferreira-Paim et al, PlosNTD,2017 |
| UFTM 14.45 | Urine | Clinical | Brazil | 2005 | Positive | 63 | 63 | South America | Ferreira-Paim et al, PlosNTD,2017 |
| UFTM 14.57 | Bronchoalveolar lavage | Clinical | Brazil | 2005 | Positive | 63 | 63 | South America | Ferreira-Paim et al, PlosNTD,2017 |
| UFTM 14.79 | Cerebrospinal fluid | Clinical | Brazil | 2009 | Positive | 63 | 63 | South America | Ferreira-Paim et al, PlosNTD,2017 |
| UFTM 14.83 | Cerebrospinal fluid | Clinical | Brazil | 2013 | Positive | 63 | 63 | South America | Ferreira-Paim et al, PlosNTD,2017 |
| UFTM 14.121 | Cerebrospinal fluid | Clinical | Brazil | 2003 | Positive | 63 | 63 | South America | Ferreira-Paim et al, PlosNTD,2017 |
| UFTM 14.159 | Cerebrospinal fluid | Clinical | Brazil | 2010 | Positive | 63 | 63 | South America | Ferreira-Paim et al, PlosNTD,2017 |
| UFTM 14.53 | Urine | Clinical | Brazil | 2006 | Positive | 71 | 63 | South America | Ferreira-Paim et al, PlosNTD,2017 |
| UFTM 14.98 | Cerebrospinal fluid | Clinical | Brazil | 2001 | Positive | 71 | 63 | South America | Ferreira-Paim et al, PlosNTD,2017 |
| UFTM 14.161 | Cerebrospinal fluid | Clinical | Brazil | 2006 | Positive | 71 | 63 | South America | Ferreira-Paim et al, PlosNTD,2017 |
| UFTM 14.147 | Cerebrospinal fluid | Clinical | Brazil | 2000 | Positive | 289 | 63 | South America | Ferreira-Paim et al, PlosNTD,2017 |
| PU162 | CSF | Clinical | China | Unknown | Positive | 63 | 63 | East Asia | Khayhan et al., (2013) |
| 10-0493 | Unknown | Clinical | Germany | 2010 | Positive | 23 | 63 | Europe | Sanchini et al. Med Microbiol Immunol. 2014,203(5):333-340 |
| 10-0457 | Unknown | Clinical | Germany | 2010 | Positive | 69 | 63 | Europe | Sanchini et al. Med Microbiol Immunol. 2014,203(5):333-340 |
| 07-0325 | Unknown | Clinical | Germany | 2007 | Positive | 71 | 63 | Europe | Sanchini et al. Med Microbiol Immunol. 2014,203(5):333-340 |
| 25_328 | CSF | Clinical | India | 2007 | Positive | 71 | 63 | South Asia | Khayhan et al., (2013) |
| 264 | CSF | Clinical | Indonesia | 2006 | Positive | 69 | 63 | Southeast Asia | Khayhan et al., (2013) |
| 2478 | CSF | Clinical | Indonesia | 2006 | Positive | 69 | 63 | Southeast Asia | Khayhan et al., (2013) |
| IUM 01-3463 | Blood | Clinical | Italy | #N/A | Positive | 56 | 63 | Europe | Cogliati et al. Medical Mycology. 2013,51:499–506 |
| IUM 99-5716 | Cerebrospinal fluid | Clinical | Italy | #N/A | Positive | 61 | 63 | Europe | Cogliati et al. Medical Mycology. 2013,51:499–506 |
| IUM 98-3890 | Cerebrospinal fluid | Clinical | Italy | #N/A | Positive | 62 | 63 | Europe | Cogliati et al. Medical Mycology. 2013,51:499–506 |
| IUM 97-4634 | Blood | Clinical | Italy | #N/A | Positive | 65 | 63 | Europe | Cogliati et al. Medical Mycology. 2013,51:499–506 |
| IUM 97-4874 | Cerebrospinal fluid | Clinical | Italy | #N/A | Positive | 68 | 63 | Europe | Cogliati et al. Medical Mycology. 2013,51:499–506 |
| IUM 99-5673 | Cerebrospinal fluid | Clinical | Italy | #N/A | Positive | 69 | 63 | Europe | Cogliati et al. Medical Mycology. 2013,51:499–506 |
| IUM 99-5719 | Cerebrospinal fluid | Clinical | Italy | #N/A | Positive | 138 | 63 | Europe | Cogliati et al. Medical Mycology. 2013,51:499–506 |
| K37 | Cerebrospinal fluid | Clinical | Korea | #N/A | Positive | 23 | 63 | East Asia | Choi et al. FEMS Yeast Res. 2010,10(6):769–778 |
| mal 120 | Blood | Clinical | Malawi | #N/A | Positive | 2 | 63 | Africa | Litvintseva et al. Genetics. 2006,172(4):2223–2238 |
| 246 | CSF | Clinical | South Africa |  | Positive | 2 | 63 | Africa | Beale MA et al, Plos NTD,2015 |
| 308 | CSF | Clinical | South Africa |  | Positive | 2 | 63 | Africa | Beale MA et al, Plos NTD,2015 |
| 472 | CSF | Clinical | South Africa |  | Positive | 2 | 63 | Africa | Beale MA et al, Plos NTD,2015 |
| 310 | CSF | Clinical | South Africa |  | Positive | 2 | 63 | Africa | Beale MA et al, Plos NTD,2015 |
| 102 | CSF | Clinical | South Africa |  | Positive | 58 | 63 | Africa | Beale MA et al, Plos NTD,2015 |
| 256 | CSF | Clinical | South Africa |  | Positive | 58 | 63 | Africa | Beale MA et al, Plos NTD,2015 |
| 250 | CSF | Clinical | South Africa |  | Positive | 67 | 63 | Africa | Beale MA et al, Plos NTD,2015 |
| 127 | CSF | Clinical | South Africa |  | Positive | 234 | 63 | Africa | Beale MA et al, Plos NTD,2015 |
| 119 | CSF | Clinical | South Africa |  | Positive | 235 | 63 | Africa | Beale MA et al, Plos NTD,2015 |
| 260 | CSF | Clinical | South Africa |  | Positive | 246 | 63 | Africa | Beale MA et al, Plos NTD,2015 |
| 9257 | Avian guano | Environmental | Japan | 2005 | NA | 23 | 63 | East Asia | Khayhan et al., (2013) |
| 8_92 | CSF | Clinical | Kuwait | Unknown | Negative | 23 | 63 | Middle East | Khayhan et al., (2013) |
| 154 | CSF | Clinical | South Africa |  | Positive | 23 | 63 | Africa | Beale MA et al, Plos NTD,2015 |
| 210 | CSF | Clinical | South Africa |  | Positive | 23 | 63 | Africa | Beale MA et al, Plos NTD,2015 |
| 258 | CSF | Clinical | South Africa |  | Positive | 23 | 63 | Africa | Beale MA et al, Plos NTD,2015 |
| 212 | CSF | Clinical | South Africa |  | Positive | 23 | 63 | Africa | Beale MA et al, Plos NTD,2015 |
| 223 | CSF | Clinical | South Africa |  | Positive | 23 | 63 | Africa | Beale MA et al, Plos NTD,2015 |
| 251 | CSF | Clinical | South Africa |  | Positive | 23 | 63 | Africa | Beale MA et al, Plos NTD,2015 |
| 412 | CSF | Clinical | South Africa |  | Positive | 23 | 63 | Africa | Beale MA et al, Plos NTD,2015 |
| 316 | CSF | Clinical | South Africa |  | Positive | 23 | 63 | Africa | Beale MA et al, Plos NTD,2015 |
| 357 | CSF | Clinical | South Africa |  | Positive | 23 | 63 | Africa | Beale MA et al, Plos NTD,2015 |
| Isolate4 | Unknown | Unknown | France | Unknown | Unknown | 323 | 63 | Europe | http://mlst.mycologylab.org/ |
| A3 1-1 | Avian guano | Enviromental | USA | 2002 | NA | 23 | 63 | North America | Litvintseva et al. Genetics. 2006,172(4):2223–2238; Litvintseva et al. PlosOne. 2011, 6(5):e19688 |
| blg12 | Air in zoo | Enviromental | Belgium | Unknown | NA | 23 | 63 | Europe | Litvintseva et al. Genetics. 2006,172(4):2223–2238 |
| 362 | CSF | Clinical | South Africa |  | Positive | 23 | 63 | Africa | Beale MA et al, Plos NTD,2015 |
| 352 | CSF | Clinical | South Africa |  | Positive | 23 | 63 | Africa | Beale MA et al, Plos NTD,2015 |
| 403 | CSF | Clinical | South Africa |  | Positive | 23 | 63 | Africa | Beale MA et al, Plos NTD,2015 |
| 415 | CSF | Clinical | South Africa |  | Positive | 23 | 63 | Africa | Beale MA et al, Plos NTD,2015 |
| 461 | CSF | Clinical | South Africa |  | Positive | 23 | 63 | Africa | Beale MA et al, Plos NTD,2015 |
| 433 | CSF | Clinical | South Africa |  | Positive | 23 | 63 | Africa | Beale MA et al, Plos NTD,2015 |
| UFTM 14.90 | Cerebrospinal fluid | Clinical | Brazil | 2013 | Kidney transplant | 23 | 63 | South America | Ferreira-Paim et al, PlosNTD,2017 |
| 442 | CSF | Clinical | South Africa |  | Positive | 23 | 63 | Africa | Beale MA et al, Plos NTD,2015 |
| 481 | CSF | Clinical | South Africa |  | Positive | 23 | 63 | Africa | Beale MA et al, Plos NTD,2015 |
| 483 | CSF | Clinical | South Africa |  | Positive | 23 | 63 | Africa | Beale MA et al, Plos NTD,2015 |
| 147 | CSF | Clinical | South Africa |  | Positive | 247 | 63 | Africa | Beale MA et al, Plos NTD,2015 |
| 254 | CSF | Clinical | South Africa |  | Positive | 248 | 63 | Africa | Beale MA et al, Plos NTD,2015 |
| 216 | CSF | Clinical | South Africa |  | Positive | 63 | 63 | Africa | Beale MA et al, Plos NTD,2015 |
| 224 | CSF | Clinical | South Africa |  | Positive | 63 | 63 | Africa | Beale MA et al, Plos NTD,2015 |
| 249 | CSF | Clinical | South Africa |  | Positive | 63 | 63 | Africa | Beale MA et al, Plos NTD,2015 |
| 104 | CSF | Clinical | South Africa |  | Positive | 69 | 63 | Africa | Beale MA et al, Plos NTD,2015 |
| 146 | CSF | Clinical | South Africa |  | Positive | 69 | 63 | Africa | Beale MA et al, Plos NTD,2015 |
| 125 | CSF | Clinical | South Africa |  | Positive | 69 | 63 | Africa | Beale MA et al, Plos NTD,2015 |
| 126 | CSF | Clinical | South Africa |  | Positive | 69 | 63 | Africa | Beale MA et al, Plos NTD,2015 |
| 129 | CSF | Clinical | South Africa |  | Positive | 69 | 63 | Africa | Beale MA et al, Plos NTD,2015 |
| 135 | CSF | Clinical | South Africa |  | Positive | 69 | 63 | Africa | Beale MA et al, Plos NTD,2015 |
| 139 | CSF | Clinical | South Africa |  | Positive | 69 | 63 | Africa | Beale MA et al, Plos NTD,2015 |
| 207 | CSF | Clinical | South Africa |  | Positive | 69 | 63 | Africa | Beale MA et al, Plos NTD,2015 |
| 208 | CSF | Clinical | South Africa |  | Positive | 69 | 63 | Africa | Beale MA et al, Plos NTD,2015 |
| 235 | CSF | Clinical | South Africa |  | Positive | 69 | 63 | Africa | Beale MA et al, Plos NTD,2015 |
| 241 | CSF | Clinical | South Africa |  | Positive | 69 | 63 | Africa | Beale MA et al, Plos NTD,2015 |
| 253 | CSF | Clinical | South Africa |  | Positive | 69 | 63 | Africa | Beale MA et al, Plos NTD,2015 |
| 255 | CSF | Clinical | South Africa |  | Positive | 69 | 63 | Africa | Beale MA et al, Plos NTD,2015 |
| 202 | CSF | Clinical | South Africa |  | Positive | 69 | 63 | Africa | Beale MA et al, Plos NTD,2015 |
| 203 | CSF | Clinical | South Africa |  | Positive | 69 | 63 | Africa | Beale MA et al, Plos NTD,2015 |
| 225 | CSF | Clinical | South Africa |  | Positive | 69 | 63 | Africa | Beale MA et al, Plos NTD,2015 |
| 231 | CSF | Clinical | South Africa |  | Positive | 69 | 63 | Africa | Beale MA et al, Plos NTD,2015 |
| JH125.91 | Clinical | Clinical | Tanzania | Unknown | Unknown | 3 | 31 | Africa | Litvintseva et al. Genetics. 2006,172(4):2223–2238 |
| fr1 | Avian guano | Environmental | France | #N/A | NA | 1 | 63 | Europe | Litvintseva et al. Genetics. 2006,172(4):2223–2238 |
| 04-0202 | Unknown | Clinical | Germany | 2004 | Negative | 2 | 63 | Europe | Sanchini et al. Med Microbiol Immunol. 2014,203(5):333-340 |
| 232 | CSF | Clinical | South Africa |  | Positive | 69 | 63 | Africa | Beale MA et al, Plos NTD,2015 |
| 233 | CSF | Clinical | South Africa |  | Positive | 69 | 63 | Africa | Beale MA et al, Plos NTD,2015 |
| K1 | Urine | Clinical | Korea | Unknown | Unknown | 5 | 5 | East Asia | Choi et al. FEMS Yeast Res. 2010,10(6):769–778 |
| 245 | CSF | Clinical | South Africa |  | Positive | 69 | 63 | Africa | Beale MA et al, Plos NTD,2015 |
| 262 | CSF | Clinical | South Africa |  | Positive | 69 | 63 | Africa | Beale MA et al, Plos NTD,2015 |
| 365 | CSF | Clinical | South Africa |  | Positive | 69 | 63 | Africa | Beale MA et al, Plos NTD,2015 |
| 371 | CSF | Clinical | South Africa |  | Positive | 69 | 63 | Africa | Beale MA et al, Plos NTD,2015 |
| NIIDCr0012 | Clinical | Clinical | Japan | Unknown | Unknown | 85 | 5 | East Asia | Umeyama et al. Jpn. J. Infect. Dis. 2013,66(1):51-55 |
| NIIDCr0013 | Clinical | Clinical | Japan | Unknown | Unknown | 4 | 4 | East Asia | Umeyama et al. Jpn. J. Infect. Dis. 2013,66(1):51-55 |
| 402 | CSF | Clinical | South Africa |  | Positive | 69 | 63 | Africa | Beale MA et al, Plos NTD,2015 |
| 436 | CSF | Clinical | South Africa |  | Positive | 69 | 63 | Africa | Beale MA et al, Plos NTD,2015 |
| 439 | CSF | Clinical | South Africa |  | Positive | 69 | 63 | Africa | Beale MA et al, Plos NTD,2015 |
| 318 | CSF | Clinical | South Africa |  | Positive | 69 | 63 | Africa | Beale MA et al, Plos NTD,2015 |
| 413 | CSF | Clinical | South Africa |  | Positive | 69 | 63 | Africa | Beale MA et al, Plos NTD,2015 |
| 416 | CSF | Clinical | South Africa |  | Positive | 69 | 63 | Africa | Beale MA et al, Plos NTD,2015 |
| NIIDCr0029 | Clinical | Clinical | Japan | Unknown | Unknown | 63 | 63 | East Asia | Umeyama et al. Jpn. J. Infect. Dis. 2013,66(1):51-55 |
| 464 | CSF | Clinical | South Africa |  | Positive | 69 | 63 | Africa | Beale MA et al, Plos NTD,2015 |
| NIIDCr0036 | Clinical | Clinical | Japan | Unknown | Unknown | 230 | 63 | East Asia | Umeyama et al. Jpn. J. Infect. Dis. 2013,66(1):51-55 |
| 405 | CSF | Clinical | South Africa |  | Positive | 69 | 63 | Africa | Beale MA et al, Plos NTD,2015 |
| 414 | CSF | Clinical | South Africa |  | Positive | 69 | 63 | Africa | Beale MA et al, Plos NTD,2015 |
| 418 | CSF | Clinical | South Africa |  | Positive | 69 | 63 | Africa | Beale MA et al, Plos NTD,2015 |
| P6 | Clinical | Clinical | Thailand | Unknown | Unknown | 4 | 4 | Southeast Asia | Khayhan et al., (2013) |
| 110_99 | Blood | Clinical | Kuwait | 1999 | Negative | 69 | 63 | Middle East | Khayhan et al., (2013) |
| 112 | CSF | Clinical | South Africa |  | Positive | 212 | 63 | Africa | Beale MA et al, Plos NTD,2015 |
| 213 | CSF | Clinical | South Africa |  | Positive | 218 | 63 | Africa | Beale MA et al, Plos NTD,2015 |
| 227 | CSF | Clinical | South Africa |  | Positive | 218 | 63 | Africa | Beale MA et al, Plos NTD,2015 |
| 215 | CSF | Clinical | South Africa |  | Positive | 218 | 63 | Africa | Beale MA et al, Plos NTD,2015 |
| Tn470 | Blood | Clinical | Tanzania | #N/A | Positive | 2 | 63 | Africa | Litvintseva et al. Genetics. 2006,172(4):2223–2238 |
| UFTM 14.43 | Enviromental | Enviromental | Brazil | #N/A | NA | 71 | 63 | South America | Ferreira-Paim et al, PlosNTD,2017 |
| ug2459 | Cerebrospinal fluid | Clinical | Uganda | #N/A | Positive | 23 | 63 | Africa | Litvintseva et al. Genetics. 2006,172(4):2223–2238 |
| UgCl057 | Cerebrospinal fluid | Clinical | Uganda | #N/A | Positive | 63 | 63 | Africa | Wiesner et al. mBio. 2012,3(5):e00196-12 |
| UgCl030 | Cerebrospinal fluid | Clinical | Uganda | #N/A | Positive | 69 | 63 | Africa | Wiesner et al. mBio. 2012,3(5):e00196-12 |
| UFTM 14.110 | CSF | Clinical | Brazil | 2002 | Unknown | 93 | 31 | South America | Ferreira-Paim et al, PlosNTD,2017 |
| 08-0445 | Unknown | Clinical | Germany | 2008 | Negative | 290 | 63 | Europe | Sanchini et al. Med Microbiol Immunol. 2014,203(5):333-340 |
| UFTM 14.134 | CSF | Clinical | Brazil | 2003 | Unknown | 93 | 31 | South America | Ferreira-Paim et al, PlosNTD,2017 |
| UFTM 14.135 | CSF | Clinical | Brazil | 2008 | Unknown | 93 | 31 | South America | Ferreira-Paim et al, PlosNTD,2017 |
| UFTM 14.154 | CSF | Clinical | Brazil | Unknown | Unknown | 93 | 31 | South America | Ferreira-Paim et al, PlosNTD,2017 |
| A3 38-20 | Avian guano | Environmental | USA | 2002 | NA | 1 | 63 | North America | Litvintseva et al. Genetics. 2006,172(4):2223–2238 |
| A1 | Avian guano | Environmental | USA | 2002 | NA | 2 | 63 | North America | Litvintseva et al. Genetics. 2006,172(4):2223–2238; Simwami et al. PLoS Pathogens. 2011,7(4):e1001343 |
| A4 1-12 | Avian guano | Enviromental | USA | 2002 | NA | 58 | 63 | North America | Litvintseva et al. Genetics. 2006,172(4):2223–2238; Litvintseva et al. PlosOne. 2011, 6(5):e19688 |
| JS25 | Veterinay | Veterinary | USA | #N/A | NA | 64 | 63 | North America | Singer et al. J Clin Microbiol. 2014, 52(6):2061-2070 |
| UFTM 14.91 | CSF | Clinical | Brazil | Unknown | Unknown | 93 | 31 | South America | Ferreira-Paim et al, PlosNTD,2017 |
| WM 148 | CSF | Clinical | Australia | 1995 | Unknown | 63 | 63 | Australasia | Litvintseva et al. Genetics. 2006,172(4):2223–2238 |
| UFTM 14.20 | Enviromental | Environmental | Brazil | Unknown | NA | 2 | 63 | South America | Ferreira-Paim et al, PlosNTD,2017 |
| WH125 | CSF | Clinical | China | 2007 | Positive | 53 | 174 | East Asia | Khayhan et al., (2013) |
| 05-0070 | Unknown | Clinical | Germany | 2005 | Positive | 174 | 174 | Europe | Sanchini et al. Med Microbiol Immunol. 2014,203(5):333-340 |
| 25_296 | CSF | Clinical | India | 2007 | Positive | 174 | 174 | South Asia | Khayhan et al., (2013) |
| IUM 97-4877 | Cerebrospinal fluid | Clinical | Italy | #N/A | Positive | 55 | 174 | Europe | Cogliati et al. Medical Mycology. 2013,51:499–506 |
| IUM 00-1072 | Cerebrospinal fluid | Clinical | Italy | #N/A | Positive | 81 | 174 | Europe | Cogliati et al. Medical Mycology. 2013,51:499–506 |
| 200_16 | Lym node biopsy | Clinical | Kuwait | 1996 | Positive | 175 | 174 | Middle East | Khayhan et al., (2013) |
| WH018 | CSF | Clinical | China | 2003 | Negative | 53 | 174 | East Asia | Khayhan et al., (2013) |
| WH070 | CSF | Clinical | China | 1993 | Negative | 53 | 174 | East Asia | Khayhan et al., (2013) |
| WH073 | CSF | Clinical | China | 1999 | Negative | 53 | 174 | East Asia | Khayhan et al., (2013) |
| WH113 | CSF | Clinical | China | 2007 | Negative | 53 | 174 | East Asia | Khayhan et al., (2013) |
| 34 | Avian guano | Environmental | Thailand | Unknown | NA | 53 | 174 | Southeast Asia | Khayhan et al., (2013) |
| 25_17 | Blood | Clinical | India | 1989 | Negative | 174 | 174 | South Asia | Khayhan et al., (2013) |
| 25_240 | CSF | Clinical | India | 2005 | Negative | 174 | 174 | South Asia | Khayhan et al., (2013) |
| 2365_08 | CSF | Clinical | Kuwait | 2008 | Negative | 174 | 174 | Middle East | Khayhan et al., (2013) |
| D45 | Avian guano | Environmental | Thailand | 2000 | NA | 175 | 174 | Southeast Asia | Khayhan et al., (2013) |
| 1589_04 | CSF | Clinical | Kuwait | 2004 | Negative | 192 | 174 | Middle East | Khayhan et al., (2013) |
| PMHc1052.ENR.STOR | CSF | Clinical | Botswana | 2012 | Positive | 9 | N/A | Africa | Chen et al., (2016) |
| bt150 | Cerebrospinal fluid | Clinical | Botswana | 2001 | Positive | 21 | N/A | Africa | Litvintseva et al. Genetics. 2006,172(4):2223–2238; Litvintseva et al. PlosOne. 2011, 6(5):e19688 |
| PMHc1023.ENR | CSF | Clinical | Botswana | 2012 | Positive | 40 | N/A | Africa | Chen et al., (2016) |
| NRHc5048.ENR.ISO | CSF | Clinical | Botswana | 2012 | Positive | 80 | N/A | Africa | Chen et al., (2016) |
| PMHc1043.ENR.STOR | CSF | Clinical | Botswana | 2012 | Positive | 80 | N/A | Africa | Chen et al., (2016) |
| PMHc1034.ENR.STOR | CSF | Clinical | Botswana | 2012 | Positive | 89 | N/A | Africa | Chen et al., (2016) |
| NRHc5031.ENR.CLIN.ISO | CSF | Clinical | Botswana | 2012 | Positive | 143 | N/A | Africa | Chen et al., (2016) |
| NRHc5017.ENR | CSF | Clinical | Botswana | 2012 | Positive | 317 | N/A | Africa | Chen et al., (2016) |
| NRHc5001.ENR | CSF | Clinical | Botswana | 2012 | Positive | 378 | N/A | Africa | Chen et al., (2016) |
| PMHc1031A.ENR.INI.LP | CSF | Clinical | Botswana | 2012 | Positive | 379 | N/A | Africa | Chen et al., (2016) |
| PMHc1020.CLIN.1 | CSF | Clinical | Botswana | 2012 | Positive | 380 | N/A | Africa | Chen et al., (2016) |
| NRHc5041.ENR.CLIN.ISO | CSF | Clinical | Botswana | 2012 | Positive | 384 | N/A | Africa | Chen et al., (2016) |
| PMHc1038.ENR.CLIN1 | CSF | Clinical | Botswana | 2012 | Positive | 385 | N/A | Africa | Chen et al., (2016) |
| NRHc5030.ENR.CLIN.ISO | CSF | Clinical | Botswana | 2012 | Positive | 386 | N/A | Africa | Chen et al., (2016) |
| NRHc5013.ENR | CSF | Clinical | Botswana | 2012 | Positive | 387 | N/A | Africa | Chen et al., (2016) |
| PMHc1001.ENR | CSF | Clinical | Botswana | 2012 | Positive | 389 | N/A | Africa | Chen et al., (2016) |
| NRHc5009.ENR | CSF | Clinical | Botswana | 2012 | Positive | 392 | N/A | Africa | Chen et al., (2016) |
| NRHc5037.ENR.CLIN1 | CSF | Clinical | Botswana | 2012 | Positive | 394 | N/A | Africa | Chen et al., (2016) |
| PMHc1014.ENR | CSF | Clinical | Botswana | 2012 | Positive | 396 | N/A | Africa | Chen et al., (2016) |
| NRHc5005.ENR | CSF | Clinical | Botswana | 2012 | Positive | 398 | N/A | Africa | Chen et al., (2016) |
| PMHc1030.ENR.CLIN.ISO | CSF | Clinical | Botswana | 2012 | Positive | 402 | N/A | Africa | Chen et al., (2016) |
| NRHc5006.CLIN.1 | CSF | Clinical | Botswana | 2012 | Positive | 403 | N/A | Africa | Chen et al., (2016) |
| NRHc5008.ENR | CSF | Clinical | Botswana | 2012 | Positive | 407 | N/A | Africa | Chen et al., (2016) |
| PMHc1047.ENR.CLIN1 | CSF | Clinical | Botswana | 2012 | Positive | 408 | N/A | Africa | Chen et al., (2016) |
| PMHc1024.CLIN.1 | CSF | Clinical | Botswana | 2012 | Positive | 409 | N/A | Africa | Chen et al., (2016) |
| PMHc1033.ENR | CSF | Clinical | Botswana | 2012 | Positive | 410 | N/A | Africa | Chen et al., (2016) |
| PMHc1035.ENR.STOR | CSF | Clinical | Botswana | 2012 | Positive | 411 | N/A | Africa | Chen et al., (2016) |
| NRHc5040.ENR.CLIN.ISO | CSF | Clinical | Botswana | 2012 | Positive | 415 | N/A | Africa | Chen et al., (2016) |
| NRHc5026.ENR.CLIN.ISO | CSF | Clinical | Botswana | 2012 | Positive | 416 | N/A | Africa | Chen et al., (2016) |
| PMHc1050.ENR.CLIN1 | CSF | Clinical | Botswana | 2012 | Positive | 417 | N/A | Africa | Chen et al., (2016) |
| NRHc5019.ENR | CSF | Clinical | Botswana | 2012 | Positive | 419 | N/A | Africa | Chen et al., (2016) |
| NRHc5023.ENR | CSF | Clinical | Botswana | 2012 | Positive | 421 | N/A | Africa | Chen et al., (2016) |
| PMHc1026.ENR | CSF | Clinical | Botswana | 2012 | Positive | 422 | N/A | Africa | Chen et al., (2016) |
| NRHc5020.CLIN.1 | CSF | Clinical | Botswana | 2012 | Positive | 424 | N/A | Africa | Chen et al., (2016) |
| NRHc5011.ENR | CSF | Clinical | Botswana | 2012 | Positive | 427 | N/A | Africa | Chen et al., (2016) |
| NRHc5032.ENR.CLIN.ISO | CSF | Clinical | Botswana | 2012 | Positive | 428 | N/A | Africa | Chen et al., (2016) |
| NRHc5021.CLIN.1 | CSF | Clinical | Botswana | 2012 | Positive | 429 | N/A | Africa | Chen et al., (2016) |
| NRHc5029.ENR.CLIN.ISO | CSF | Clinical | Botswana | 2012 | Positive | 432 | N/A | Africa | Chen et al., (2016) |
| NRHc5039.ENR.CLIN.ISO | CSF | Clinical | Botswana | 2012 | Positive | 433 | N/A | Africa | Chen et al., (2016) |
| NRHc5024.ENR.CLIN.1 | CSF | Clinical | Botswana | 2012 | Positive | 434 | N/A | Africa | Chen et al., (2016) |
| NRHc5004.ENR | CSF | Clinical | Botswana | 2012 | Positive | 435 | N/A | Africa | Chen et al., (2016) |
| NRHc5045.ENR.CLIN.ISO | CSF | Clinical | Botswana | 2012 | Positive | 436 | N/A | Africa | Chen et al., (2016) |
| NRHc5035.ENR.CLIN.ISO | CSF | Clinical | Botswana | 2012 | Positive | 438 | N/A | Africa | Chen et al., (2016) |
| NRHc5042.ENR.STOR | CSF | Clinical | Botswana | 2012 | Positive | 447 | N/A | Africa | Chen et al., (2016) |
| NRHc5015.ENR | CSF | Clinical | Botswana | 2012 | Positive | 449 | N/A | Africa | Chen et al., (2016) |
| PMHc1022.ENR | CSF | Clinical | Botswana | 2012 | Positive | 449 | N/A | Africa | Chen et al., (2016) |
| PMHc1032.ENR | CSF | Clinical | Botswana | 2012 | Positive | 449 | N/A | Africa | Chen et al., (2016) |
| NRHc5012.CLIN.1 | CSF | Clinical | Botswana | 2012 | Positive | 450 | N/A | Africa | Chen et al., (2016) |
| NRHc5014.ENR | CSF | Clinical | Botswana | 2012 | Positive | 451 | N/A | Africa | Chen et al., (2016) |
| PMHc1049.THER1.STOR | CSF | Clinical | Botswana | 2012 | Positive | 460 | N/A | Africa | Chen et al., (2016) |
| PMHc1011.ENR | CSF | Clinical | Botswana | 2012 | Positive | 464 | N/A | Africa | Chen et al., (2016) |
| NRHc5027.ENR.CLIN1 | CSF | Clinical | Botswana | 2012 | Positive | 465 | N/A | Africa | Chen et al., (2016) |
| PMHc1045.ENR.STOR | CSF | Clinical | Botswana | 2012 | Positive | 467 | N/A | Africa | Chen et al., (2016) |
| PMHc1002.ENR | CSF | Clinical | Botswana | 2012 | Positive | 468 | N/A | Africa | Chen et al., (2016) |
| PMHc1009.ENR | CSF | Clinical | Botswana | 2012 | Positive | 469 | N/A | Africa | Chen et al., (2016) |
| NRHc5022.ENR | CSF | Clinical | Botswana | 2012 | Positive | 472 | N/A | Africa | Chen et al., (2016) |
| PMHc1029.ENR.STOR | CSF | Clinical | Botswana | 2012 | Positive | 478 | N/A | Africa | Chen et al., (2016) |
| PMHc1040.ENR.STOR | CSF | Clinical | Botswana | 2012 | Positive | 483 | N/A | Africa | Chen et al., (2016) |
| NRHc5010.ENR | CSF | Clinical | Botswana | 2012 | Positive | 484 | N/A | Africa | Chen et al., (2016) |
| WH132 | CSF | Clinical | China | 2007 | Positive | 195 | N/A | East Asia | Khayhan et al., (2013) |
| 25_291 | CSF | Clinical | India | 2006 | Negative | 40 | N/A | South Asia | Khayhan et al., (2013) |
| Gbc16-1 | dead tree | Environmental | Botswana | 2012 | NA | 80 | N/A | Africa | Chen et al., (2016) |
| Ftc95-3 | Mopane tree | Environmental | Botswana | 2012 | NA | 89 | N/A | Africa | Chen et al., (2016) |
| Ftc98-1 | Mopane tree | Environmental | Botswana | 2012 | NA | 377 | N/A | Africa | Chen et al., (2016) |
| Gbc51-2 | Acacia sp. | Environmental | Botswana | 2012 | NA | 378 | N/A | Africa | Chen et al., (2016) |
| Gbc42-2 | Acacia sp. | Environmental | Botswana | 2012 | NA | 381 | N/A | Africa | Chen et al., (2016) |
| Ftc321-1 | Mopane tree | Environmental | Botswana | 2012 | NA | 382 | N/A | Africa | Chen et al., (2016) |
| Gbc39-1 | Acacia sp. | Environmental | Botswana | 2012 | NA | 383 | N/A | Africa | Chen et al., (2016) |
| Ftc103-1 | Mopane tree | Environmental | Botswana | 2012 | NA | 388 | N/A | Africa | Chen et al., (2016) |
| Ftc170-1 | Mopane tree | Environmental | Botswana | 2012 | NA | 390 | N/A | Africa | Chen et al., (2016) |
| Ftc102-1 | Mopane tree | Environmental | Botswana | 2012 | NA | 391 | N/A | Africa | Chen et al., (2016) |
| Ftc322-1 | Mopane tree | Environmental | Botswana | 2012 | NA | 393 | N/A | Africa | Chen et al., (2016) |
| Ftc327-1 | Mopane tree | Environmental | Botswana | 2012 | NA | 393 | N/A | Africa | Chen et al., (2016) |
| Ftc195-1 | Mopane tree | Environmental | Botswana | 2012 | NA | 395 | N/A | Africa | Chen et al., (2016) |
| Muc525-1 | soil | Environmental | Botswana | 2012 | NA | 397 | N/A | Africa | Chen et al., (2016) |
| Ftc239-1 | Mopane tree | Environmental | Botswana | 2012 | NA | 399 | N/A | Africa | Chen et al., (2016) |
| Ftc214-1 | Mopane tree | Environmental | Botswana | 2012 | NA | 400 | N/A | Africa | Chen et al., (2016) |
| Ftc209-1 | Mopane tree | Environmental | Botswana | 2012 | NA | 401 | N/A | Africa | Chen et al., (2016) |
| Ftc241-1 | Mopane tree | Environmental | Botswana | 2012 | NA | 404 | N/A | Africa | Chen et al., (2016) |
| Ftc217-1 | Mopane tree | Environmental | Botswana | 2012 | NA | 405 | N/A | Africa | Chen et al., (2016) |
| Ftc211-1 | Mopane tree | Environmental | Botswana | 2012 | NA | 406 | N/A | Africa | Chen et al., (2016) |
| Ftc158-1 | Avian guano | Environmental | Botswana | 2012 | NA | 412 | N/A | Africa | Chen et al., (2016) |
| Ftc167-1 | Mopane tree | Environmental | Botswana | 2012 | NA | 413 | N/A | Africa | Chen et al., (2016) |
| Ftc267-1 | Mopane tree | Environmental | Botswana | 2012 | NA | 413 | N/A | Africa | Chen et al., (2016) |
| Muc402-1 | Mopane tree | Environmental | Botswana | 2012 | NA | 414 | N/A | Africa | Chen et al., (2016) |
| Gbc574-1 | Ziziphus mucronata | Environmental | Botswana | 2012 | NA | 418 | N/A | Africa | Chen et al., (2016) |
| Muc470-1 | Mopane tree | Environmental | Botswana | 2012 | NA | 418 | N/A | Africa | Chen et al., (2016) |
| Muc479-1 | Mopane tree | Environmental | Botswana | 2012 | NA | 420 | N/A | Africa | Chen et al., (2016) |
| Muc416-1 | Acacia sp. | Environmental | Botswana | 2012 | NA | 423 | N/A | Africa | Chen et al., (2016) |
| Muc468-1 | Mopane tree | Environmental | Botswana | 2012 | NA | 423 | N/A | Africa | Chen et al., (2016) |
| Ftc200-1 | Mopane tree | Environmental | Botswana | 2012 | NA | 425 | N/A | Africa | Chen et al., (2016) |
| Ftc192-1 | Mopane tree | Environmental | Botswana | 2012 | NA | 426 | N/A | Africa | Chen et al., (2016) |
| Ftc222-1 | Mopane tree | Environmental | Botswana | 2012 | NA | 430 | N/A | Africa | Chen et al., (2016) |
| Ftc257-1 | Mopane tree | Environmental | Botswana | 2012 | NA | 431 | N/A | Africa | Chen et al., (2016) |
| Gbc44-1 | Acacia sp. | Environmental | Botswana | 2012 | NA | 437 | N/A | Africa | Chen et al., (2016) |
| Ftc168-1 | Mopane tree | Environmental | Botswana | 2012 | NA | 439 | N/A | Africa | Chen et al., (2016) |
| Gbc573-1 | Ziziphus mucronata | Environmental | Botswana | 2012 | NA | 440 | N/A | Africa | Chen et al., (2016) |
| Muc449-1 | Ziziphus mucronata | Environmental | Botswana | 2012 | NA | 441 | N/A | Africa | Chen et al., (2016) |
| Muc498-1 | Mopane tree | Environmental | Botswana | 2012 | NA | 441 | N/A | Africa | Chen et al., (2016) |
| Muc499-1 | Mopane tree | Environmental | Botswana | 2012 | NA | 441 | N/A | Africa | Chen et al., (2016) |
| Ftc555-1 | Mopane tree | Environmental | Botswana | 2012 | NA | 442 | N/A | Africa | Chen et al., (2016) |
| Muc418-1 | Acacia sp. | Environmental | Botswana | 2012 | NA | 443 | N/A | Africa | Chen et al., (2016) |
| Muc421-3 | Acacia sp. | Environmental | Botswana | 2012 | NA | 443 | N/A | Africa | Chen et al., (2016) |
| Muc460-1 | Mopane tree | Environmental | Botswana | 2012 | NA | 443 | N/A | Africa | Chen et al., (2016) |
| Ftc146-1 | Mopane tree | Environmental | Botswana | 2012 | NA | 444 | N/A | Africa | Chen et al., (2016) |
| Muc364-1 | Mopane tree | Environmental | Botswana | 2012 | NA | 445 | N/A | Africa | Chen et al., (2016) |
| Ftc109-1 | Mopane tree | Environmental | Botswana | 2012 | NA | 446 | N/A | Africa | Chen et al., (2016) |
| Ftc260-1 | Mopane tree | Environmental | Botswana | 2012 | NA | 448 | N/A | Africa | Chen et al., (2016) |
| Ftc151-1 | Mopane tree | Environmental | Botswana | 2012 | NA | 452 | N/A | Africa | Chen et al., (2016) |
| Ftc153-1 | Mopane tree | Environmental | Botswana | 2012 | NA | 453 | N/A | Africa | Chen et al., (2016) |
| Ftc132-1 | Mopane tree | Environmental | Botswana | 2012 | NA | 454 | N/A | Africa | Chen et al., (2016) |
| Ftc134-1 | Mopane tree | Environmental | Botswana | 2012 | NA | 454 | N/A | Africa | Chen et al., (2016) |
| Ftc225-1 | Mopane tree | Environmental | Botswana | 2012 | NA | 455 | N/A | Africa | Chen et al., (2016) |
| Muc451-1 | Mopane tree | Environmental | Botswana | 2012 | NA | 456 | N/A | Africa | Chen et al., (2016) |
| Ftc137-1 | Mopane tree | Environmental | Botswana | 2012 | NA | 457 | N/A | Africa | Chen et al., (2016) |
| Ftc152-2 | Mopane tree | Environmental | Botswana | 2012 | NA | 457 | N/A | Africa | Chen et al., (2016) |
| Ftc111-1 | Mopane tree | Environmental | Botswana | 2012 | NA | 458 | N/A | Africa | Chen et al., (2016) |
| Ftc173-1 | Mopane tree | Environmental | Botswana | 2012 | NA | 459 | N/A | Africa | Chen et al., (2016) |
| Muc387-1 | Mopane tree | Environmental | Botswana | 2012 | NA | 461 | N/A | Africa | Chen et al., (2016) |
| Muc437-1 | Ziziphus mucronata | Environmental | Botswana | 2012 | NA | 462 | N/A | Africa | Chen et al., (2016) |
| Muc367-1 | Mopane tree | Environmental | Botswana | 2012 | NA | 463 | N/A | Africa | Chen et al., (2016) |
| Muc466-1 | Mopane tree | Environmental | Botswana | 2012 | NA | 466 | N/A | Africa | Chen et al., (2016) |
| Muc415-1 | Mopane tree | Environmental | Botswana | 2012 | NA | 470 | N/A | Africa | Chen et al., (2016) |
| Muc450-1 | Mopane tree | Environmental | Botswana | 2012 | NA | 471 | N/A | Africa | Chen et al., (2016) |
| Muc489-1 | Mopane tree | Environmental | Botswana | 2012 | NA | 471 | N/A | Africa | Chen et al., (2016) |
| Muc504-1 | Mopane tree | Environmental | Botswana | 2012 | NA | 471 | N/A | Africa | Chen et al., (2016) |
| Muc529-1 | Mopane tree | Environmental | Botswana | 2012 | NA | 471 | N/A | Africa | Chen et al., (2016) |
| BK55 | CSF | Clinical | Vietnam | Unknown | Positive | 340 | 31 | Southeast Asia | This study |
| Ftc207-1 | Mopane tree | Environmental | Botswana | 2012 | NA | 473 | N/A | Africa | Chen et al., (2016) |
| Ftc236-1 | Mopane tree | Environmental | Botswana | 2012 | NA | 474 | N/A | Africa | Chen et al., (2016) |
| Muc463-2 | Mopane tree | Environmental | Botswana | 2012 | NA | 475 | N/A | Africa | Chen et al., (2016) |
| Muc507-1 | Mopane tree | Environmental | Botswana | 2012 | NA | 476 | N/A | Africa | Chen et al., (2016) |
| Ftc154-1 | Mopane tree | Environmental | Botswana | 2012 | NA | 477 | N/A | Africa | Chen et al., (2016) |
| Muc503-1 | Mopane tree | Environmental | Botswana | 2012 | NA | 479 | N/A | Africa | Chen et al., (2016) |
| Muc433-1 | Acacia sp. | Environmental | Botswana | 2012 | NA | 480 | N/A | Africa | Chen et al., (2016) |
| Muc457-1 | Mopane tree | Environmental | Botswana | 2012 | NA | 480 | N/A | Africa | Chen et al., (2016) |
| Muc458-1 | Mopane tree | Environmental | Botswana | 2012 | NA | 480 | N/A | Africa | Chen et al., (2016) |
| Muc516-1 | Mopane tree | Environmental | Botswana | 2012 | NA | 481 | N/A | Africa | Chen et al., (2016) |
| Ftc202-1 | Mopane tree | Environmental | Botswana | 2012 | NA | 482 | N/A | Africa | Chen et al., (2016) |
| bt121 | Cerebrospinal fluid | Clinical | Botswana | 2001 | Positive | 34 | N/A | Africa | Litvintseva et al. Genetics. 2006,172(4):2223–2238; Litvintseva et al. PlosOne. 2011, 6(5):e19688 |
| bt9 | Cerebrospinal fluid | Clinical | Botswana | 1999 | Positive | 36 | N/A | Africa | Litvintseva et al. Genetics. 2006,172(4):2223–2238; Litvintseva et al. PlosOne. 2011, 6(5):e19688 |
